# Mitochondrial phylogeny and taxonomic revision of Italian and Slovenian fluvio-lacustrine barbels, *Barbus* sp. (Cypriniformes, Cyprinidae)

**DOI:** 10.1186/s40850-021-00073-x

**Published:** 2021-04-21

**Authors:** Giovanni Rossi, Federico Plazzi, Gianluca Zuffi, Andrea Marchi, Salvatore De Bonis, Marco Valli, Petra Marinšek, Rosanna Falconi

**Affiliations:** 1 Department of Biological, Geological and Environmental Sciences, University of Bologna, Bologna, Italy; 2 Hydrosynergy SC, San Lazzaro di Savena, Italy; 3 Present Address: Sezione di Bologna, Arpae Emilia-Romagna, Bologna, Italy; 4 Servizio Monitoraggio Risorse Idriche, Arpa Lazio, Rome, Italy; 5 Present Address: Regione Emilia-Romagna, Servizio Attività Faunistico Venatorie e Pesca, Bologna, Italy; 6 Faculty of Natural Science and Mathematics, University of Maribor, Maribor, Slovenia; 7 Present Address: Hochdorf Swiss Nutrition AG, Hochdorf, Switzerland

**Keywords:** Phylogeography, Fluvio-lacustrine barbels, Mitochondrial markers, Ichthyogeographic districts, Taxonomy

## Abstract

**Background:**

Barbels are ray finned cyprinid fishes of the Old-World with partially unresolved, intricate taxonomy. Within the *Barbus* sensu *lato* paraphyletic assemblage, *Barbus* sensu stricto is a monophyletic tetraploid lineage of Europe, northern Africa and Middle East, including two monophyletic sibling genera: *Barbus* and *Luciobarbus*. Italy, Slovenia and northern Croatia are natively inhabited by several entities of the genus *Barbus*, whose relationships and taxonomic ranks are still unclear. Aim of the present work is to focus on phylogeography of Italian and Slovenian barbels, with an appraisal of their current taxonomy.

**Results:**

One hundred fifty specimens were collected in 78 sampling sites from 33 main watersheds, widely distributed along Italian and Slovenian ichthyogeographic districts. We amplified two mitochondrial markers, cytochrome b (*cytb*) and control region (D-loop), to infer a robust phylogeny for our sample and investigate on species delimitation.

Our results strongly indicate all Italian and Adriatic Slovenian fluvio-lacustrine barbels to be comprised into at least three distinct species. We provide a proposal of taxonomic revision and a list of synonymies for two of them and a new description under the International Code of Zoological Nomenclature rules for the third one.

**Conclusions:**

If nuclear data will confirm our findings, at least three specific entities should be acknowledged across our sampling area. Namely, the three species are (i) *Barbus plebejus*, in the Padano-Venetian district; (ii) *Barbus tyberinus*, in the Tuscany-Latium district; (iii) *Barbus oscensis* Rossi & Plazzi sp. nov., in the Tyrrhenian and southernmost-Adriatic parts of Apulia-Campania district. Finally, we briefly discuss the implications of such a taxonomic scenario on conservation policies.

**Supplementary Information:**

The online version contains supplementary material available at 10.1186/s40850-021-00073-x.

## Background

The taxonomy of barbels (Actinopterygii: Cyprinidae) is a vexing question which remains partially unresolved. *Barbus* sensu *lato* is a paraphyletic taxon of the Old World, which has normally three levels of ploidy and is comprised by at least 800 species [1]. Conversely, *Barbus* sensu stricto (*Barbus* Cuvier and Cloquet 1816 sensu [2, 3]) is a monophyletic tetraploid genus of Europe, northern Africa and Middle East [4].

Doadrio [5] proposed further morphological subdivision of this taxon into two monophyletic sister subgenera: the nominotypical *Barbus,* and *Luciobarbus* Heckel 1843: the former is distributed in the northern part of the distribution range, whereas the latter is distributed in the southern watersheds.

Further molecular analyses confirmed the existence of these two clades within *Barbus s. s* [6–13].. They are currently recognised as distinct genera [14–21], and a third genus, *Aulopyge* Heckel, 1841, has been found to be closely related to the ancestor of both and has been included in the group [4, 9]. *Aulopyge* is a monotypic genus, with *A. huegelii* living in Croatia and Bosnia-Herzegovina.

**Fig. 1:**
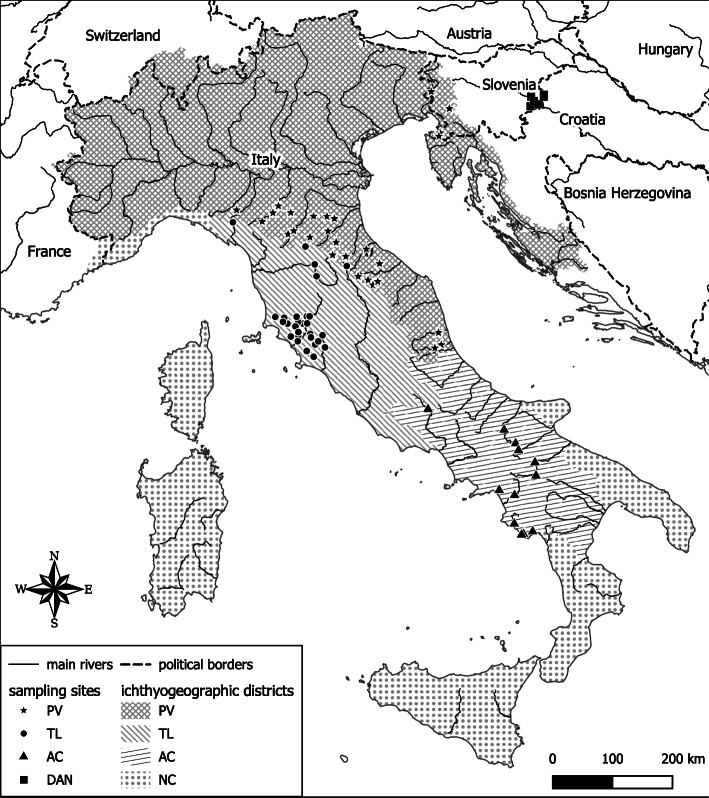
Approximate delimitation of ichthyogeographic districts in the area of interest (redrawn from districts in [22, 26, 27]) and localization of specimen collection. Abbreviations: PV, Padano-Venetian district; TL, Tuscany-Latium district; AC, Apulia-Campania district; DAN, Danubian district, NC, not classified as district. Map was generated by GR using the software QGIS 2.18 (https://qgis.org/it/site/index.html)

The Italian peninsula is currently subdivided into three different districts or regions for what ichthyogeography is concerned. The Padano-Venetian district (PV [22, 23]), alias Padan region [24], corresponds to the Paleo-Po last glacial maximum catchment and includes Adriatic rivers of Slovenia and Croatia as well. Nearly all the remaining Italian mainland is subdivided into a northern Tuscany-Latium district (TL [22, 23]) and a southern Apulia-Campania district (AC [25]); territories (including also Italian islands and Corsica) with poorness or absence of primary freshwater fish species [22, 26] are not classified as districts or regions (Fig. 1).

Native Italian barbels belong to genus *Barbus* [5, 14, 28] and are currently enlisted as four different species [15]: *Barbus caninus* Bonaparte 1839; *Barbus balcanicus* Kotlík et al. 2002; *Barbus plebejus* Bonaparte 1839; *Barbus tyberinus* Bonaparte 1839.

*Barbus caninus* and *B. balcanicus* are two rheophilic sibling vicariant species of the PV that also inhabit few TL and many not-Adriatic Dinaric/Balkan drainages, respectively [15, 18, 28–31]; although human manipulation cannot be excluded, their distribution may be linked to natural river capture phenomena.

As stated in [15, 28, 31, 32], the fluvio-lacustrine *B. plebejus* and *B. tyberinus* are vicariant sibling species too: the former native of PV [33], the latter naturally inhabiting TL and part of AC [34]. Other authors [35, 36] claimed these to be two morpho-species sensu Ruse [37], and pointed out that morphological differences between populations could be highly influenced by environmental factors, or caused by hybridization phenomena. Therefore, they do not recognize *B. tyberinus* as a valid species and include its populations and distribution range in those of *B. plebejus*.

Further insights did not settle the issue: focusing on intra- versus inter-group phenotypic similarity/dissimilarity, the existence of the two morpho-species was claimed by Lorenzoni et al. [38], but rejected by Livi et al. [39]. Nevertheless, even if the scenario proposed by Livi et al. [39] is correct, the two taxa may well represent geographically isolated, cryptic species sensu Bickford et al. [40].

Within genus *Barbus* introgressive hybridization has been repeatedly documented [18, 31, 41–46], occurring either in natural hybrid zones, (e.g., *B. balcanicus* × *B. plebejus* and *B. caninus* × *B. plebejus* [18]) or after a human-mediated secondary contact, e.g. between *B. plebejus*, *B. tyberinus* and *B. barbus* (L.) [18, 45, 47], the latter being a native species of the Danubian basin.

In fact, indigenous Italian barbel distribution is altered by anthropic activities [24], and invasive species introduction plays a key role as well [48], so that the existence of introgressive hybridization phenomena could hamper the analysis and disentanglement of morphological and genetic differentiation between the native *Barbus* species.

Despite that the taxonomic ranking debate is still unresolved, the use of the name *B. tyberinus* increased over time by leading organizations as the International Union for Conservation of Nature [49] – Comitato Italiano IUCN [50], the Institute for Biodiversity Science and Sustainability [51], and the Species 2000 and ITIS Integrated Taxonomic Information System [52]. Unstable nomenclature could cause wildlife management aberrations, with population of *B. tyberinus* possibly losing the conservation status they had as a part of *B. plebejus* (species listed in Annexes II and V of the European Union Habitats Directive and in Appendix III of the Bern Convention).

Recently, Buonerba et al. [18] analysed mitochondrial and nuclear markers in *Barbus* specimens from PV and TL and demonstrated the genetic distinguishability and close relatedness of *B. tyberinus* and *B. plebejus*. The genetic distinguishability of *B. tyberinus* and *B. plebejus* was also observed by [53, 54] using fragments of the D-loop control region and cytochrome *b* gene. Interestingly, they also identified two new, distinct and allopatric *Barbus* clades. The former was only found in Eastern Central Italy, in the Adriatic watersheds of Vomano [53], Aterno-Pescara, Sangro and Biferno [54]. The latter is distributed in Adriatic watersheds to the south of Biferno river (Fortore and Ofanto) and in southernmost investigated Tyrrhenian watersheds (Liri-Garigliano, Volturno and Sele) [54].

The existence of genetically distinct allopatric taxa at least identifies them as Evolutionary Significant Units [55], but their taxonomic rank remains undefined. Nonetheless, phylogeographic structures and genetic similarity/dissimilarity between and within territories could be a diagnostic key for the definition of ichthyogeographic (or more generally biogeographic) districts [56–58]. Therefore, AC can be split into its northern Adriatic part (NAAC) and its Tyrrhenian and southern Adriatic part (TSAAC).

Specifically, in the cited works, nine sampling sites from AC were analysed covering four out of the five main watersheds of NAAC (lacking Trigno river basin), but only five out of the 14 main watersheds of TSAAC (missing, north to south, the Adriatic Candelaro, Carapelle, Cervaro and the Tyrrhenian Savone, Sarno, Tusciano, Alento, Mingardo, Bussento river basins) in which *Barbus* species are possibly autochthonous according to Bianco [28]. Moreover, two mitochondrial markers were used, but the sequencing of the fragment of the cytochrome *b* gene was limited to a subsample of 26 specimens showing 26 different D-loop haplotypes [54].

In this work, we analysed phylogenetic relationships within Italian barbels increasing the number of analysed localities, primarily in TSAAC and in the Slovenian part of PV. We present a thorough phylogenetic reconstruction of Italian barbels using two mitochondrial markers for each specimen instead of collapsing to haplotypes. This allowed to assess genetic and morphological differences in terms of similarity/dissimilarity between and within clades. We focused on the hypothesis of the existence of two new species previously undescribed under the rules of the International Code of Zoological Nomenclature (ICZN). In this scenario, the current nomenclature in use for Italian fluvio-lacustrine barbels would be obsolete, since the two putative new species are until now enlisted as part of *B. tyberinus*.

We then found supporting evidence for the description of a new species corresponding to the TSAAC clade, for which we propose the name *B. oscensis*, though we advise caution until present data are confirmed by thorough molecular studies using also nuclear markers. Finally, we also review range, description and synonymy for the already described *B. plebejus* and *B. tyberinus* and give suggestions for conservation policies of Italian barbels.

## Methods

### Specimen collection, morphologic analysis, identification and preparation

After acquiring the relevant approvals and permissions to collect animals, we collected 138 specimens in 72 sampling sites from 32 main watersheds (i.e., basins of rivers emptying into a sea), widely distributed along PV, TL and AC districts (29, 31 and 12 sampling sites, respectively; Fig. 1), to which 12 specimens in 6 sampling sites from the Danubian district have been added (DAN; Fig. 1). The distribution of sampling sites per district, main watershed and river is provided in Additional file 1. No animals were excluded from subsequent analyses.

Specimens were caught using electrofishing devices with direct current and electrical settings that minimize possible stresses to the animals [59], made unconscious with the anaesthetic 2-phenoxyethanol 0,5 ml/L, and rapidly identified utilizing meristic and qualitative external characters (not to harm any specimen) as in the dichotomous key of Kottelat and Freyhof [15] for barbels of Apennine Peninsula and Adriatic basin of Slovenia and Croatia, provided as Additional file 2. Fishes were then biopsied regardless of morphological determination and released alive and conscious in the place where caught. Biopsies (clips of caudal fin small enough to avoid a significant effect on specimens’ motility) were stored in 100% ethanol and refrigerated at 4 °C.

Six specimens from a single target sampling site belonging to the new clade described in this work were collected, humanely euthanized and fixed in 10% formalin for museum conservation and conforming to Directive 2010/63/EU (provided that all the permissions requested under the Italian law had been granted). Morphological description of the six specimens was done on the basis of morphometrics measures (eye diameter; preorbital distance; mouth-operculum distance; length of pectoral fin; length of ventral fin; length of anal fin; height of the third dorsal fin ossified ray) and meristic counts (branched rays in the dorsal, anal and pelvic fins; scales on the lateral line, above the lateral line, below the lateral line; number of circumpeduncolar scales; gill rakers in the lower arch and in the upper arch; total gill rakers; pharyngeal teeth in the left and in the right side; serrae on ossified ray of fin), following previous works on this species complex [28, 33, 54, 60].

### PCR amplification and sequencing

DNA was extracted from caudal fin clips stored in 100% ethanol using a standard phenol:chloroform protocol [61]. PCR amplification of portions of cytochrome b gene (*cytb*) and tRNA-Pro/control region (*trnP*/D-loop) was carried out with GoTaq® Flexi DNA Polymerase (Promega, Madison, WI, USA), as follows: 10 μL 5× Green GoTaq® Flexi Buffer, MgCl2 (3 mM), ddNTPs (800 μM each), primers (500 nM each), 1 U GoTaq® DNA Polymerase, 40 ng template DNA, ddH_2_O up to 50 μL. Primers were designed for the present study and are BARBUS 8 (5′-GCGCTAGGGAGGAGTTTA-3′) and BARBUS 5 (5’TTTTAACCGAGACCAATGAC-3′) for *cytb* and dloop sxF (D1) (5′-AAAGCATCGGTCTTGTAATC-3′) and dloop dxR (D2) (5′-GAGTTTTCTAGGACCCATCTTA-3′) for *trnP*/D-loop. Annealing settings were 60 °C/35″ and 55 °C–58 °C/35″, respectively. PCR results were visualized using a 1% electrophoresis agarose gel stained with ethidium bromide. Amplicons were purified and sequenced at the Macrogen Europe facility (Amsterdam, The Netherlands). Electropherograms were edited using MEGA 7 [62].

### Phylogenetic analyses

This paper presents the first phylogenetic reconstruction of Italian barbels using two mitochondrial markers for each specimen. To our knowledge, all the previous papers focusing on Italian barbels (e.g., [18, 45, 53, 54, 63]) presented haplotypes instead of sequences of single individuals. As a consequence, it was not possible to include all the available literature in tree reconstruction, with the only exception of [54], who published the association of 19 D-loop haplotypes with the respective *cytb* haplotype: these sequences were added to our dataset. *Cyprinus carpio* (L.) and *Luciobarbus graellsii* (Steindachner, 1866) sequences were downloaded from GenBank (Accession Numbers DQ868875/JN105352 and JN049525/MG827110, respectively) and used as outgroups.

The T-Coffee [64] algorithm was used for single alignments. Nucleotide sequences of *cytb* and D-loop were separately aligned through the M-Coffee approach, starting from MAFFT [65] and Muscle [66] libraries. Sites with low or noisy phylogenetic signal were masked using Gblocks 0.91b [67]. The *cytb* alignment was further subdivided into the three codon positions using a custom-tailored Python script (available from FP upon request), obtaining five datasets: cytb1_1, cytb1_2, cytb1_3, *trnP*, and dloop.

The datasets were concatenated into the final dataset and PartitionFinder 1.1.0 [68] was used to decide whether to apply a single-partition or a multiple-partition scheme, as well as to select molecular evolution models; the Bayesian Information Criterion and the greedy approach were chosen. Three methods were then selected to reconstruct the phylogenetic tree of Italian barbels.

First, the software RaxML 8.2.11 [69] was used for the Maximum Likelihood (ML) inference under the CAT model. The Best-Known Likelihood Tree (BKLT) was computed and then it was annotated with bootstrap support values and using 1000 bootstrap replicates. Furthermore, Bayesian Inference was also carried out with MrBayes 3.2.7 [70] using two separate runs, four chains, and 10,000,000 generations of MC^3^, sampling every 100 trees. Convergence between runs and burn-in were estimated looking to four diagnostics: standard deviation of average split frequencies sampled every 1000 generation, Potential Scale Reduction Factor (PSRF [71]), plot of log-likelihoods on the MCMC generations, and minimum Estimated Sample Sizes. Finally, the phylogenetic inference was carried out using IQ-TREE 1.7-beta7 [72] with 1000 ultrafast bootstrap replicates [73]. In the IQ-TREE analysis, substitution models were selected using ModelFinder [74] and the best partitioning scheme was selected with the greedy strategy implemented in ModelFinder [68, 75].

### Taxonomic unit definition and networks

Two different barcode gap (see, f.i., [76, 77]) approaches were explored to test taxonomic unit definition among the native Italian barbel clades (*B. plebejus*, *B. tyberinus*, *Barbus* sp. clade 4, and *Barbus* sp. TSAAC clade). First, we applied the Automatic Barcode Gap Discovery (ABGD [78]), using the K-2-P distance and a transition/transversion ratio set to 2. Prior intraspecific distance ranged from 0.001 and 0.01, with 20 steps. Moreover, we used the Species Delimitation Plugin of Geneious Prime® 2021.0.3 (Biomatters Ltd., Auckland, New Zealand) to compute the Rosenberg’s P_AB_ [79] and the mean probabilities of making a correct identification of an unknown specimen of the focal taxon following [80].

Given the short length of the amplified *trnP* fragment (76 bp), the minimum spanning network was computed from multiple *cytb* and D-loop sequence alignments only. Newly produced sequences were added to previous data taken from [18, 45, 53, 54, 63]; the software PopART v 1.7 [81] was used to draw the network, using the statistical parsimony criterion and setting ε = 0. Data from literature were assigned the relative geographical abundancies upon retrieval in the original publication. Unfortunately, the geographical information associated to some Tyrrhenian watershed haplotypes by [54] was missing or misspelt (see Table 1 in [54]), therefore we could not unambiguously assign them to either TSAAC or NAAC district. All sequences used for single-marker minimum spanning networks are listed in Additional file 3. Conversely, data from the present work refer to single specimens, therefore an abundancy of 1 was assigned to the relative ichthyogeographical district (see Additional file 3). Finally, for the combined *cytb*-dloop network, we retained only those 155 entries for which an association between *cytb* and dloop was available; the analysis was carried out as above.

### Morphological analysis

Data registered for the formal description of the new clade types were added to those of the corresponding SI2 lineage of [54] and compared to other Italian clades from the same work (*B barbus*, *B. plebejus*, *B. tyberinus*, and the SI1 lineage, corresponding to the NAAC lineage). Single measurements were not published in [54], therefore it was not possible to standardize morphometric data using the Beacham [82] formula to reduce the effects of size and allometry. Therefore, statistical analyses (ANOVA and Tukey post-hoc test) were performed exclusively on meristic traits published by [54]. Aggregate data (average value, standard deviation and sample size [54];) were merged with new data and a statistical analysis was carried out using custom scripts available from FP upon request.

Meristic data used for the formal description of the types of the new clade were compared to the values of *B. plebejus* and *B. tyberinus* published in the taxonomic revision of Bianco [28, 33, 34], which includes the respective type specimens. Since maximum ranges and usual vales (generically defined) are only reported therein, no quantitative measure of data dispersion was available; hence, no statistical test was performed and only a graphical comparison was carried out.

### Nomenclatural acts

The electronic version of this article in portable document format represents a published work according to the International Commission on Zoological Nomenclature (ICZN), and hence the new name contained in the electronic version are effectively published under that Code from the electronic edition alone (see Articles 8.5–8.6 of the Code). This published work and the nomenclatural acts it contains have been registered in ZooBank (http://zoobank.org/), the online registration system for the ICZN. The ZooBank LSIDs (Life Science Identifiers) can be resolved and the associated information viewed through any standard web browser by appending the LSID to the prefix “http://zoobank.org/”. The LSID for this publication is: urn:lsid:zoobank.org:pub:BE76B7A1-8FF7-4903-A1F7-9D52D03EAD83. The electronic edition of this work was published in a journal with an ISSN, and has been archived and is available from the following digital repositories: PubMed Central, LOCKSS.

## Results

### Phylogenetic analysis

The final dataset is composed by 171 sequences, including the only 19 sequences that were possible to obtain from [54] and two outgroups: *C. carpio* and *L. graellsii*. However, three specimens (GR633Omb11BA, GR570Usi200BA, and GR568Usi200BA) from our sample turned out to be *L. graellsii* as well*.* Indeed, this Iberian species is enlisted within Italian non-native barbels [17, 83]: thus, it is unsurprising to sporadically catch *L. graellsii* specimens among native Italian barbels.

The final *cytb* alignment is 1141 bp long, while the final *trnP*/D-loop alignment is 620 bp long (including 76 and 544 bp of *trnP* and D-loop, respectively). Both markers were amplified from all individuals: new sequences were uploaded to GenBank under Accession Numbers MG495623-MG495922. Specimens vouchers, taxonomy following our phylogenetic reconstruction, locality (country, river, altitude) and accession numbers are provided as Additional file 4.

The strategy selected by PartitionFinder was to keep all markers together in a single partition and the best-fitting molecular evolutionary model was TrN + I + G. Conversely, the ModelFinder algorithm of IQ-TREE selected a partitioning scheme with four partitions: cytb_1 + *trnP*, cytb_2, cytb_3, and dloop. The best-fitting molecular evolutionary models were K-2-P + G4, TPM3u + F + I, TIM2 + F, and HKY + F + R3, respectively.

**Fig. 2:**
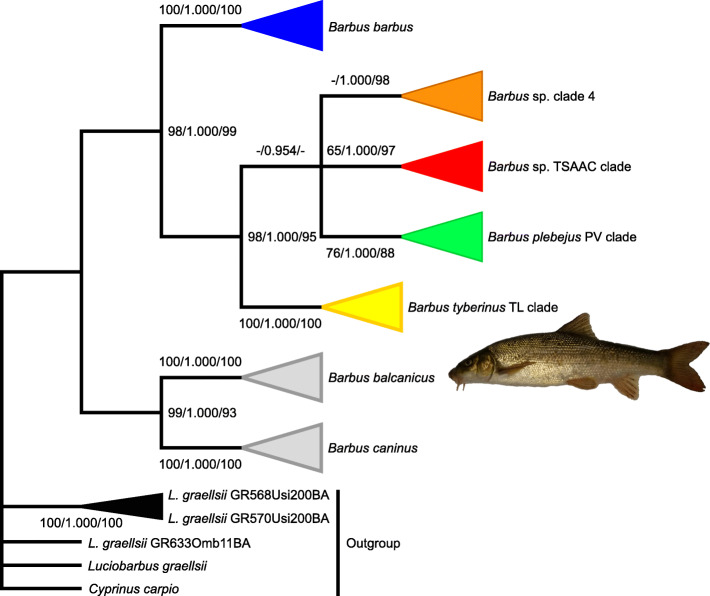
Consensus phylogenetic tree of barbels of Italy and Adriatic catchments of Slovenia and Croatia, based on combined *cytb*/*trnP*/D-loop data. *Cyprinus carpio* and *Luciobarbus graellsii* were used as outgroups. Node support values are shown in the following order: RAxML boostrap proportion (BP)/Bayesian posterior probability (PP)/IQ-TREE ultrafast bootstrap (UF_Boot) support values. For sake of clarity, only internal nodes are shown: nodes with BP < 60, PP < 0.950, and UF-Boot support value< 85 were collapsed. For complete trees, explicit GenBank Accession Numbers, and branch lengths, we refer to Additional file 5. Photograph by AM

The three trees yielded identical topologies, the only differences being in the node support values (Fig. 2): some internal nodes are not statistically supported in some trees, but topologies are never mutually exclusive (Fig. 2 and Additional file 5).

Italian fluvio-lacustrine barbels comprise a single monophyletic clade, which is highly supported, with a bootstrap proportion (BP) of 98, a posterior probability (PP) of 1.000 and an ultrafast bootstrap (UF-Boot) support value of 95. Within this clade, four main lineages were statistically evidenced. The first lineage, corresponding to *B. tyberinus*, almost exclusively encompasses samples from TL district (BP = 100; PP = 1.000, UF-Boot support value = 100). The only exception is represented by the *B. tyberinus* AQ84Lir330BA specimen caught in the Liri river, which is part of the northernmost watershed (Liri-Garigliano) of the TSAAC district, at the boundary with TL.

The second lineage, corresponding to *B. plebejus* (BP = 76; PP = 1.000; UF-Boot support value = 88.0), is mainly comprised by samples from the PV district, but few samples were also caught in TL and AC (see Additional files 4 and 5).

A third lineage (BP = 65; PP = 1.000, UF-Boot support value = 97.0) consists exclusively of samples from the TSAAC district and five out nineteen samples from [54] are nested within this clade. A possible fourth lineage (PP = 1.000; UF-Boot support value = 98) was only found in the NAAC district and is comprised by the remaining samples from [54]; this lineage was not supported as monophyletic in the RAxML analysis. Phylogenetic relationships between these four clades are not well resolved: the Bayesian inference suggest the first clade to be the sister group of a monophyletic clade comprised by the remaining three (PP = 0.954; UF-Boot support value = 61).

### Taxonomic unit definition and networks

**Fig. 3:**
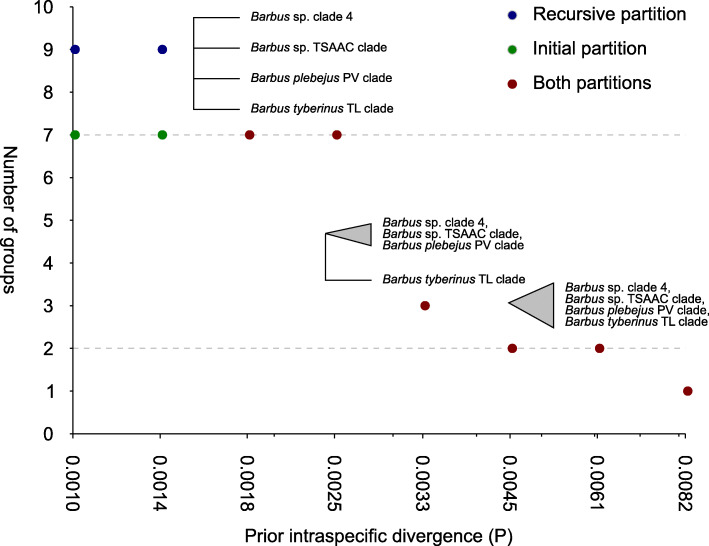
Automatic Barcode Gap Discovery. The number of identified groups is plotted over the prior intraspecific divergence used to identify the barcode gap. Major monophyletic groups, corresponding to clades in Fig. 2, are shown close to the respective points; minor groups, comprised either by a single OTU or by two OTUs, are detailed in the text. If possible, the method recursively splits (blue circles) the original partition (green circles), but in most case they overlap (red circles)

The number of groups identified in the ABGD analysis ranged from 7 to 2 moving from a prior intraspecific divergence (P) equal to 0.001/0.003 through *P* = 0.01 (Fig. 3). In the former case (low Ps), we retrieved two groups with a single specimen (either RA606Sin150BA or a specimen from [54] corresponding to Accession Numbers MK728798/MK728816), one group with two specimens (PU245Mta595BA and PU246Mta595BA), and four groups corresponding to the four clades of native Italian barbels depicted in Fig. 2. In the latter case (high Ps), a group with a single specimen (RA606Sin150BA, as before) and a group with all remaining specimens were recovered. At an intermediate *P* = 0.0033, this large assembly was split into PV specimens, one side, and all remaining specimens, the other side. The four clades were consistently recovered as reciprocally monophyletic across the three tree-building methods, with Rosenberg’s P_AB_ always smaller than 3.10 × 10^− 11^ (Additional file 6); moreover, the mean probabilities of making a correct identification of an unknown specimen of the focal taxon were always greater than 0.90, with the only exception of the NAAC district lineage, that showed slightly smaller values (Additional file 6).

**Fig. 4:**
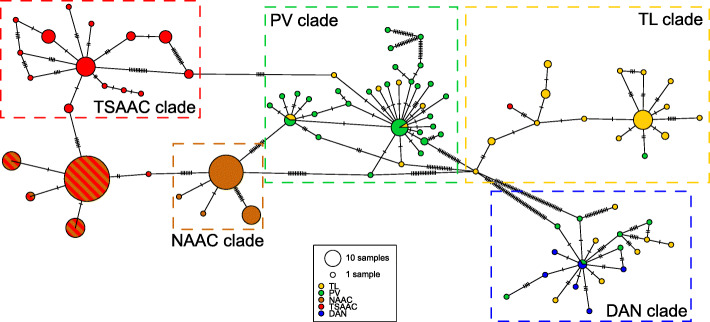
Minimum spanning network computed on the combined *cytb*/D-loop dataset, using published abundance data from [54]. Samples that was not possible to unambiguously assign to an ichthyogeographical district are shaded in red and brown

The minimum spanning network computed by the concatenated *cytb*-dloop alignment (155 sequences/1761 bp) identifies the four clades as above (Fig. 4). The cluster of Danubian haplotypes is connected to the *B. tyberinus* TL clade and then to the *B. plebejus* PV clade. From PV haplotypes, haplotypes found in TSAAC and NAAC districts are separated by few mutation steps; moreover, haplotypes of ambiguous geographical origin (see below) connect these Southern Italy lineages. However, single-marker networks including a larger amount of available data (205 sequences/1141 bp and 230 sequences/547 bp for *cytb* and dloop, respectively) yielded slightly different results: Danubian *cytb* haplotypes are connected to TSAAC haplotypes and then to PV ones, while Danubian D-loop haplotypes are connected to TL haplotypes and then to PV ones (Additional file 7). PV haplotypes are always connected to TL haplotypes, one side, and NAAC ones, the other side (Fig. 4; Additional file 7).

Within each clade, the mean *cytb* uncorrected p-distance is one order of magnitude lower than between clades; the four Italian clades show between-groups mean uncorrected p-distance values ranging from 1.55 to 2.22%, while these values rise to 3.86–4.25%, 8.31–8.96%, and 8.38–9.19% when the four Italian clades are compared with *B. barbus*, *B. balcanicus*, and *B. caninus* lineages, respectively (Table 1; see also Additional file 6). Similar results were retrieved from the D-loop fragment, which, as expected, is in some cases more variable: in some clades the intra-group mean uncorrected p-distance is greater than 1% (*B. balcanicus* and *B. caninus*). However, the four Italian clades show between-groups mean uncorrected p-distance values ranging from 0.83 to 2.64%, while these values rise to 4.86–5.74%, 7.37–8.07%, and 5.81–6.02% when the four Italian clades are compared with *B. barbus*, *B. balcanicus*, and *B. caninus* lineages, respectively (Table 1).

**Table 1:** Within- and between-groups uncorrected p-distance (mean ± standard deviation) among the seven *Barbus* lineages shown in Fig. 2

	*B. balcanicus*	*B. barbus*	*B. caninus*	*Barbus* sp. clade 4	*B. plebejus* PV clade	*B. tyberinus* TL clade	*Barbus* sp. TSAAC clade
*cytb*							
*B. balcanicus*	0.0043 ± 0.0021	0.0934 ± 0.0035	0.0679 ± 0.0055	0.0881 ± 0.0032	0.0831 ± 0.0032	0.0896 ± 0.0023	0.0836 ± 0.0023
*B. barbus*		0.0054 ± 0.0038	0.0839 ± 0.0039	0.0386 ± 0.0036	0.0403 ± 0.0049	0.0425 ± 0.0042	0.0406 ± 0.0031
*B. caninus*			0.0021 ± 0.0014	0.0865 ± 0.0049	0.0838 ± 0.0033	0.0919 ± 0.0032	0.0838 ± 0.0034
*Barbus* sp. clade 4				0.0025 ± 0.0041	0.0155 ± 0.0029	0.0216 ± 0.0022	0.0163 ± 0.0025
*B. plebejus* PV clade					0.0033 ± 0.0032	0.0183 ± 0.003	0.0166 ± 0.0026
*B. tyberinus* TL clade						0.0027 ± 0.0024	0.0222 ± 0.0025
*Barbus* sp. TSAAC clade							0.0018 ± 0.0016
dloop							
*B. balcanicus*	0.049 ± 0.0414	0.0668 ± 0.0406	0.0891 ± 0.0164	0.0753 ± 0.0175	0.0807 ± 0.017	0.0737 ± 0.0173	0.0782 ± 0.0187
*B. barbus*		0.0068 ± 0.0088	0.0682 ± 0.0107	0.0509 ± 0.0051	0.0574 ± 0.0079	0.0486 ± 0.0043	0.0516 ± 0.0055
*B. caninus*			0.0553 ± 0.0287	0.0581 ± 0.0235	0.0598 ± 0.0335	0.0601 ± 0.0258	0.0602 ± 0.0238
*Barbus* sp. clade 4				0.0085 ± 0.0069	0.0245 ± 0.0088	0.0239 ± 0.0042	0.0083 ± 0.0079
*B. plebejus* PV clade					0.0072 ± 0.0095	0.021 ± 0.0066	0.0258 ± 0.0099
*B. tyberinus* TL clade						0.0021 ± 0.0025	0.0264 ± 0.0033
*Barbus* sp. TSAAC clade							0.0062 ± 0.0087

### Morphological analysis

Morphological character values of type specimens of the TSAAC clade are reported in Additional file 8 together with meristic character values of *B. plebejus* and *B. tyberinus* in the taxonomic revision of Bianco [28, 33, 34], which includes type specimens.

Graphical morphological comparison between the three clades shows moderate to high overlapping values: the most reliable characters, number of scales on lateral line and around caudal peduncle, only permit discrimination of *B. plebejus*. The other two taxa remain indistinguishable. Graphical comparisons are provided as Additional file 9.

Meristic character values used to test morphological differences are reported in Table 2 together with ANOVA results. Meristic traits of the type specimens of the *Barbus* sp. TSAAC lineage here described were merged with those of the corresponding SI2 linage of [54] and tested against the other clades recorded by the same authors (*B. barbus*, *B. plebejus*, *B. tyberinus*, and *Barbus* sp. NAAC lineage). In all cases, results from ANOVA were highly significant (*p* < 0.001), meaning that a structure is present in the provided groups.

**Table 2:** Meristic traits of the four Italian fluvio-lacustrine barbel clades along with *Barbus barbus*. Data from 6 specimens of the *Barbus* sp. TSAAC lineage presented in this paper have been added to data published by [54]. Results from the Analysis of Variance (ANOVA) are also shown. The table lists mean ± standard deviation; observed ranges are reported in parentheses

	*Barbus* sp. NAAC lineage	*Barbus* sp. TSAAC lineage	*B tyberinus*	*B plebejus*	*B barbus*	ANOVA results	
Number of specimens	85	127	107	96	96	F	*p*
Number of dorsal fin branched rays	7*.*9 ± 0*.*4 (7–9)	8 ± 0*.*3 (7–9)	8*.*1 ± 0*.*3 (7–9)	7*.*8 ± 0*.*5 (7–9)	8*.*1 ± 0*.*3 (7–9)	12*.*6	< 0.001
Number of scales on the lateral line	55*.*8 ± 4*.*1 (50–70)	55*.*2 ± 2*.*8 (49–62)	56 ± 3*.*5 (50–66)	62*.*6 ± 3*.*8 (53–71)	56*.*9 ± 3*.*5 (49–68)	73*.*6	< 0.001
Number of scales above the lateral line	11*.*1 ± 1*.*1 (9–14)	11*.*8 ± 1*.*1 (9–15)	12*.*2 ± 1*.*3 (10–16)	13*.*4 ± 1*.*1 (10–16)	12*.*2 ± 1 (10–15)	51*.*7	< 0.001
Number of scales under the lateral line	7*.*9 ± 0*.*8 (6–10)	8*.*8 ± 0*.*8 (7–12)	8*.*5 ± 1*.*1 (6–13)	9*.*3 ± 1 (7–12)	8*.*4 ± 0*.*8 (7–10)	28*.*7	< 0.001

Tukey post-hoc test for pairwise comparisons (provided as Additional file 10), confirmed most differences between the analysed groups as significant, hence evidencing morphological differentiation between them.

Concluding, given the molecular and morphological results presented above, we propose the *Barbus* sp. TSAAC clade to be a new species: *Barbus oscensis* Rossi & Plazzi sp. nov.

## Discussion

### Phylogenetics and systematics of barbels in continental Italy

Our analysis confirms the monophyly and genetic distinguishability of at least three out of the four taxa previously identified [18, 53, 54]: a Padano-Venetian clade, a Tuscany-Latium clade, and a new clade distributed in Tyrrhenian watersheds and southernmost-Adriatic part of Apulia-Campania district, deepening genetic structure knowledge, taxonomic position and geographic distribution of the latter. The reciprocal monophyly of the two established clades and the new clade is repeatedly confirmed in our analysis, and the three groups were not identified only for relatively high values of prior intraspecific divergence, nor were *B. tyberinus* and *B. plebejus* (Fig. 3). In a nutshell, this means that, if *B. tyberinus* and *B. plebejus* are distinguished as monophyletic entities, the same must hold for the two AC *Barbus* sp. clades.

Conversely, the fourth lineage identified by [54] is distributed in the northernmost-Adriatic part of Apulia-Campania district, but further insights are required to assess its identity. In fact, some of the original coordinates [54] are lacking or result in unclear spatial reference system, and therefore geographic details assumed as valid here were taken from text and cartography of the cited work, but there is no clear indication of sampling sites. Moreover, in our phylogenetic reconstruction, this clade was not always recovered as monophyletic (Fig. 2; see also Additional file 6) and its relationships with other lineages are not unambiguously resolved and appear to be highly dependent from the dataset (see Figs. 2, 3 and 4; Additional file 7).

As is the case for previous work, our study is based on mitochondrial markers that are commonly employed in freshwater fish systematics. Therefore, we advise caution while interpreting present results, until they are confirmed by future studies using nuclear markers. A thorough genetic approach may shed light on hybridization and introgression phenomena as well, which may be easily overlooked using mitochondrial datasets. Alternative explanations of our results, such as retentions of ancestral polymorphisms and introgressions, were repeatedly observed in Barbinae subfamily [20, 41, 42, 83–88].

Nonetheless, our results are supported by available morphological data and our analyses do suggest that the three identified taxa are new species with mutually exclusive distribution. In fact, the two known exceptions can be explained either by human-mediated or natural events.

(i) Samples of Padano-Venetian lineage found outside the PV district show a patchwork pattern distribution and have no original haplotype. Moreover, Tuscany-Latium and Apulia-Campania district were historically subject to transplantation of native species from PV district [89]. This well-documented phenomenon (also known as ichthyofaunal “padanization”, from the Padan Plain) is due to governmental institutional restocking plans: public and private ichthyogenic centers located in the PV district were used as main sources of specimens starting from the end of the nineteenth century [23]. The opposite phenomenon, the transplantation of native species from Tuscany-Latium or Apulia-Campania districts to other districts, is not documented.
(ii) Three haplotypes of the TL lineage were found in the Liri-Garigliano basin in the present work, while [54] individuated two haplotypes of Tyrrhenian and southern Adriatic parts of Apulia-Campania species. The Liri-Garigliano basin is the northernmost watershed of the Apulia-Campania district at the boundary with the Tuscany-Latium district. The Gari river, which is now part of the Liri-Garigliano basin, was probably flowing in the southern Volturno basin until the growing of Roccamonfina volcano, which took place approximately 630,000 years ago [90]. This may explain the presence of haplotypes from different lineages in this river.

Indeed, each of the three Italian fluvio-lacustrine barbels species recognized here do not trespass the extension of a single Italian mainland ichthyogeographic district sensu [25].

### Taxonomy of barbels in continental Italy

The Padano-Venetian lineage fully corresponds to the species *B. plebejus*, whose first valid description (based on type specimens from the PV district only) is the one of Bonaparte in 1839 [91]. A revision of the synonymies for this species is provided as Appendix 1.

The Tuscany-Latium lineage exactly corresponds to the species originally described as *B. fluviatilis tyberinus* by Bonaparte in 1839 [91] (based on type specimens sampled in the TL district only), which is an older synonym of *B. fucini* Costa 1853; this species was previously considered [28, 34] inhabiting the Apulia-Campania district as well. A revision of the synonymies for this species is provided as Appendix 2.

**Fig. 5:**
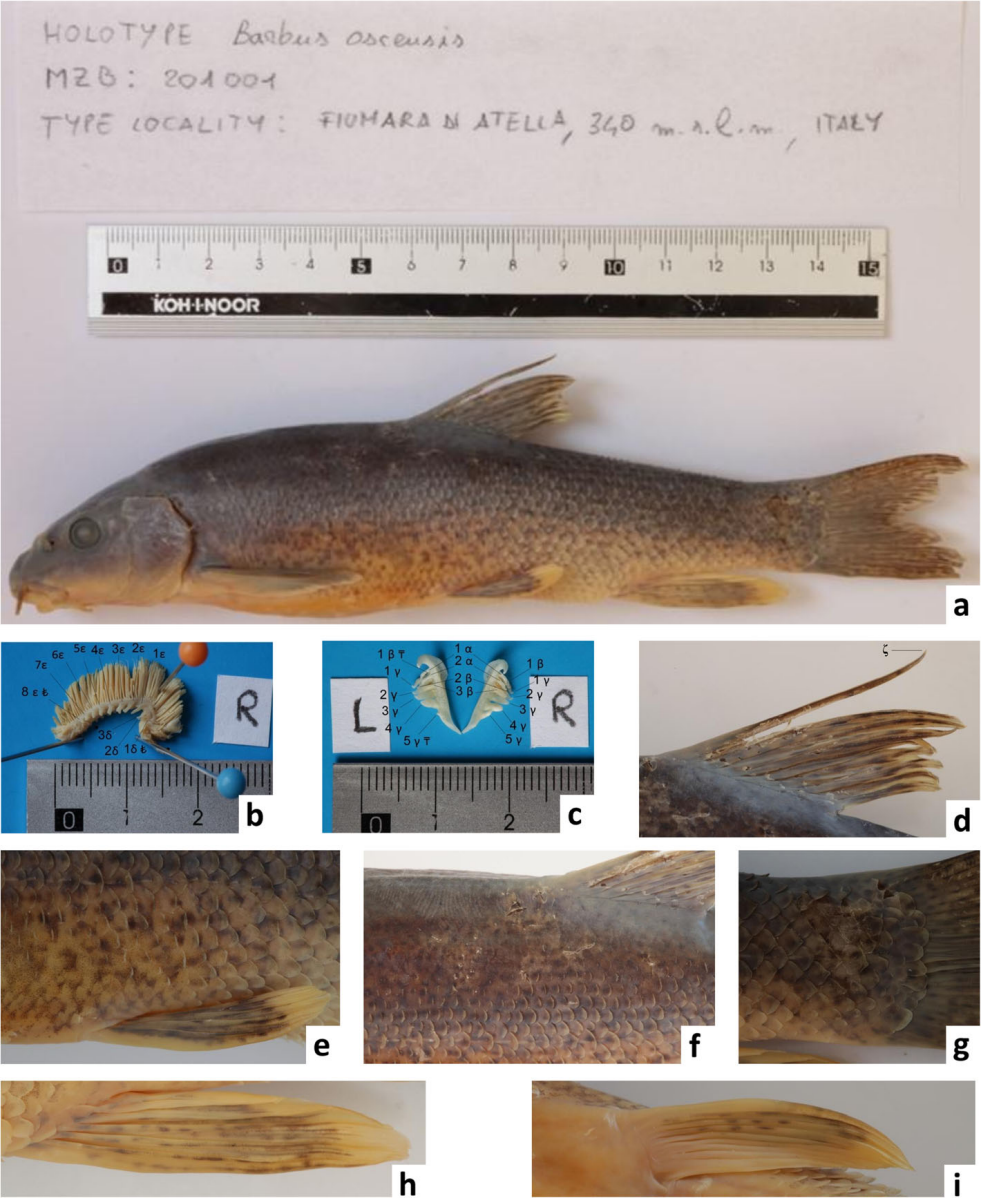
Holotype of *Barbus oscensis*; original pictures. **a** Lateral view with registration details of the Museum of Zoology of Bologna – MZB. **b** Outer gill rakers count of the first (anterior-most) gill arch on the right side. **c** Pharyngeal teeth count. **d** Dorsal fin rays, with undistinguished serration of the last unbranched ray. **e** Scales below lateral line. **f** Scales above lateral line. **g** Circumpeduncolar scales. **h** Anal fin rays. **i** Pelvic fin rays. R, right; L, left; ε, lower limb of the gill arch; δ, upper limb of the gill arch; ₺, distinctly formed rudiment; α, inner row of pharyngeal teeth; β, middle row of pharyngeal teeth; γ, outer row of pharyngeal teeth; ₸, fallen or broken tooth; ζ, last unbranched ray

Finally, the Tyrrhenian and southern Adriatic parts of Apulia-Campania lineage has no valid description as a species under the rules of the International Code of Zoological Nomenclature (ICZN). We then propose the name *B. oscensis* sp. nov. and provide the original description, diagnosis, and pictures hereafter (Fig. 5).

### Original description of *Barbus oscensis* Rossi, G. & Plazzi, F. sp. nov.

Family Cyprinidae Rafinesque 1815

Subfamily Barbinae Bleeker 1859

Genus *Barbus* Cuvier and Cloquet 1816

*Barbus oscensis* Rossi & Plazzi sp. nov. (Fig. 5)

### *Pro parte* synonymy

*Barbus fucini* Costa 1838 sensu [23]: 463 (partim: TSAAC); *Barbus fluviatilis plebejus* (non Bonaparte 1839) [92] (partim: TSAAC); *Barbus plebejus* (non Bonaparte 1839) [93]: 72–77 (partim: TSAAC) [94]: 11 (partim: TSAAC) [95]: 43 (partim: Volturno River near Venafro); [96]: 42–43 (partim: TSAAC); [97]: 40–41 (partim: TSAAC); [98]: 274–275 (partim: TSAAC); [35]: 198–202 (partim: TSAAC); *Barbus Barbus plebejus* (non Bonaparte 1839) [99]: 172 (partim: TSAAC); [100]: 19 (partim: TSAAC); [101]: 274–275 (partim: TSAAC); *Barbus tyberinus* (non Bonaparte 1839) [28]: 313–318, figs. 4b, 6–7 (Partim: TSAAC); [102]: 50–51 (Partim: TSAAC); [14]: 234 (Partim: TSAAC); [34]: 427 (Partim: TSAAC).

### Type specimens

All specimens were sampled with electrofishing from a single sampling site and conserved in alcohol 70% after formalin fixation in Museum of Zoology of Bologna (MZB).

### Holotype

MZB 201001 (GenBank Accession Numbers MG495912 and MG495762 for *cytb* and D-loop, respectively).

### Paratypes

MZB 201002–201,006 (GenBank Accession Numbers MG495913, MG495914, MG495915, MG495917, MG495918 for *cytb* and MG495763, MG495764, MG495765, MG495767, MG495768 for D-loop)*.*

### Type locality

Fiumara di Atella stream (River Ofanto watershed; Adriatic side of Southern Apennine), 340 m above sea level in Basilicata Region at the foot of Vulture Mountain in the vicinity of “il Calvario”.

### Distribution

TSAAC

### Etymology

Specific name *oscensis* is derived from Osci: the name of an ancient Italian people inhabiting a territory strongly overlapping the range of the taxon during the Iron Age.

### Diagnosis

Tyrrhenian and southern Adriatic parts of Apulia-Campania lineage shows a genetic differentiation from other fluvio-lacustrine barbel groups statistically supported in all the phylogenetic analyses hereby conducted. Moreover, the amount of genetic difference of this clade with the well-established species *B. plebejus* and *B. tyberinus* is comparable with the genetic distance between them.

Even from a morphological perspective this clade is statistically distinguishable from other fluvio-lacustrine barbel groups. However, these differences, albeit relevant to support a phylogenetic differentiation, are not reliable enough in terms of field discrimination of the different groups. This is even more true when considering that many of those differences rely (see also [54]) on morphometric traits that can only be identified after image analysis and statistic tests. In this sense, the TSAAC lineage could be considered an almost cryptic species that recently speciated from other fluvio-lacustrine species. Being a new species previously undescribed under International Code of Zoological Nomenclature rules, although yet observed as a proper taxonomic entity in other works [54], we provide a full description under the proposed name of *Barbus oscensis* sp. nov.

The holotype and paratypes of the species are conserved in the Museum of Zoology of Bologna (MZB).

### Morphology

Count and measurement are given in Additional file 8; last unbranched ray of the dorsal fin poorly ossified and with undistinguished serration; serration could be distinguishable in younger specimens as in the other two fluvio-lacustrine subspecies; ossification reduced in the distal portion of the ray.

### Remarks

*B. oscensis* sp. nov is a fluvio-lacustrine barbel species with TSAAC range, parapatric to *B. plebejus* (PV range) and *B. tyberinus* (TL range). Conversely, on the basis of morphological analysis [28, 34], *B. oscensis* was enlisted undistinguished in *B. tyberinus* species. The TSAAC range identified by the presence of this species include the Tyrrhenian basins from Liri-Garigliano to Bussento and the Adriatic basins from Fortore to Ofanto.

### Conservation and management of Italian barbels

The phylogenetic subdivision of *B. plebejus* into at least three *Barbus* species supports the partitioning of continental Italy in at least the three districts proposed by Bianco and de Filippo [25] on the basis of district-specific endemisms and subendemisms. In fact, each of the three allopatric fluvio-lacustrine barbels species hereby identified is a district-specific endemism too and an Evolutionary Significant Unit (ESU) by itself.

Results achieved by this study relies on a wide distribution of the sampling sites that, differently from previous works, covers most watersheds of Italian mainland and Slovenia. However, it must be noted that further investigation is needed in order to fully characterize the areal of the newly described species, *B. oscensis*, and verify the possibly the existence of a fourth species.

Moreover, since our results evidence a mutually exclusive range distribution of fluvio-lacustrine barbels species with morphological character moderately to strongly overlapping, it cannot be neglected that morphologically indistinguishable unknown endemisms may inhabit the few watersheds not investigated here. This is partially confirmed by the distinct lineage previously observed [53, 54], outside the range of our sampling design, whose sequences mostly comprise our *Barbus* sp. clade 4 (Fig. 2). To date, we regard at the northernmost-Adriatic part of the Apulia-Campania district as a currently unidentified district, possibly inhabited by a further undescribed species. Therefore, further investigations are needed for barbel populations of Abruzzo and Molise Regions, as well as of the Ionic side of the Basilicata Region.

## Conclusions and final remarks

The up-to-date taxonomic revision proposed in the present paper has clear effects on management and conservation policies. Historically, freshwater fish repopulations (either for sport fishing or conservation policies) were managed taking into account administrative borders and specific ranks rather than ichthyogeographic districts and ESUs. As fluvio-lacustrine cyprinids, the three species revised in the present study occupy similar habitats in their respective allopatric ranges; they can therefore be considered as vicariants species. Thus, the biology of Italian barbels and the lack of Italian laws dealing with transplantations within national borders led to indiscriminate repopulation and genetic erosion of wildlife *Barbus* populations – and the situation may get still worse.

When the Habitat Directive of the European Union (Council Directive 92/43/EEC) was written, all these species were considered as *B. plebejus*; therefore, every Italian fluvio-lacustrine barbel species should inherit the status of *B. plebejus* and deserves the proper effort in terms on conservation. In fact, each species must be protected as a separate ESU, avoiding transplantation at least between ichthyogeographic districts. On a precautionary principle, transplantation should be avoided even between different main watersheds, so that ESUs from currently unidentified districts are also protected.

Furthermore, since the taxonomy used in wildlife conservation laws (e.g., the Habitat Directive of the European Union or the Bern Convention) cannot be easily updated following scientific evidence, every wildlife conservation law addressed to any of the fluvio-lacustrine Italian barbels should be extended to any (albeit *pro parte*) possible taxonomic synonym here reported (see Discussion and Appendices 1 and 2). Concluding, since taxonomic revisions increased exponentially after the development of molecular analyses, wildlife legislation must also target ESUs and Management Units (beside species) in order to reach long-term effectiveness.

### Supplementary Information

**Additional file 1.** Sampling sites distribution, along with coordinates in EPSG 32632 reference systems.

**Additional file 2.** Dichotomous key for barbels of Apennine Peninsula and Adriatic basin of Slovenia and Croatia [15].

**Additional file 3.** GenBank Accession Numbers and geographical abundancies of sequences used for single-marker minimum spanning network computations (see Additional file 6). TL, Tuscany-Latium district; PV, Padano-Venetian district; NAAC, northernmost-Adriatic part of Apulia-Campania district; TSAAC, Tyrrhenian and southernmost-Adriatic parts of Apulia-Campania district; DAN, Danubian district; AccNum, GenBank Accession Number.

**Additional file 4.** Dataset, taxonomy, river/watershed location, sampling coordinates in EPSG 32632 reference systems and GenBank Accession Numbers.

**Additional file 5.** Original phylogenetic trees in NEXUS format as computed by IQ-TREE, MrBayes, and RAxML, respectively. Each OTU is labeled with the resulting clade (see Fig. 2) and with GenBank Accession Numbers (Additional file 4).

**Additional file 6.** Tests for chance occurrence of reciprocal monophyly. All analyses were carried out using the Species Delimitation Plugin of Geneious Prime® 2021.0.3.

**Additional file 7.** Single-marker minimum spanning networks computed from sequences and geographical abundancies listed in S4. a, cytb; b, D-loop. Asterisks mark samples that was not possible to unambiguously assign to an ichthyogeographical district (see also Fig. 4).

**Additional file 8. **Meristic data of *Barbus plebejus* and *B. tyberinus* from [28, 33, 34] and morphometric and meristic data of *B. oscensis* (i.e., the *B. tyberinus* TSAAC clade).

**Additional file 9. **Meristic data of *Barbus plebejus* (*N* = 153) and *Barbus tyberinus* (*N* = 168) from [28, 33, 34] and *Barbus oscensis* (i.e., the *B. tyberinus* TSAAC clade; *N* = 6) from original counts. Vertical lines: observed range; dithered boxes and horizontal lines: usual values. Characters with no evident variability between subspecies are not shown.

**Additional file 10. **Tukey post-hoc test results. The table lists the Q value; the *p*-value is shown in parentheses. ***, *p* < 0.001; **, *p* < 0.01. NAAC, *Barbus* sp. clade 4; TSAAC, *Barbus* sp. TSAAC clade; TL, *B. tyberinus* TL clade; PV, *B. plebejus* PV clade; DAN, *B. barbus* clade.

## Data Availability

The datasets generated during and analysed during the current study are available in the NCBI GenBank repository, under Accession Numbers MG495623-MG495922. Specimens vouchers, taxonomy following our phylogenetic reconstruction, locality (country, river, altitude) and accession numbers are provided as S5. A custom R script was developed to separate intra- and inter-clade distances and is freely available for download from GitHub (https://github.com/federicoplazzi/inter-intra). All *B. oscensis* type specimens are conserved in alcohol 70% after formalin fixation in Museum of Zoology of Bologna (MZB).

## Appendix 1

### Review of the original description of *Barbus plebejus*

Family Cyprinidae Rafinesque 1815

Subfamily Barbinae Bleeker 1859

Genus *Barbus* Cuvier and Cloquet 1816

*Barbus plebejus* Bonaparte 1839

### Synonymy

*Barbus plebejus* [91]: vol 3, no. 25, pl. 110, fig. 1 (Lake Como); Valenciennes, in [103]: 139, pl. 462 (Po River); [104]: 82, fig. 38 (near Milan and Triest); [105]: 72 (upper Adriatic Province); [106]: 142 (Isonzo-Soča River); [93]: 72 (Partim: PV); [107]: 88 (Lago Maggiore and Dalmatia); [108]: 179 (Isonzo-Soča, Zrmanja and Krka Rivers, Adriatic watershed of former Yugoslavia); [35]: 198–202 (Partim: PV); [23]: 461 (PV); [28]: 307, figs. 2-4 (Partim: Northern Italy, Adriatic Watershed of Central Italy north of Vomano excluded, Istria, Dalmazia north to the river Krka included); [14]: 128 (PV including the Zrmanja and Krka Rivers in Croatia); [33]: 339 (PV); *B. eques* [91]: vol 3, no. 25, pl. 110, fig. 2 (Partim: Northern Italy); *B. fluviatilis* non Valenciennes 1842: [92]: vol. 1, 594 (Lombardy); *B. barbus plebejus* [99]: 172, Tab I, figs. 6,7 (Italy); [109]: 100 (Northern Italy); [101]: X, 274, fig. 11A (Partim: PV); *B. plebejus plebejus* [110]: 200 (partim: PV); [111]: 154 (Partim: PV).

### Type specimens

The type specimens defined by Bianco [33] for *B. plebejus* within syntypes of Bonaparte’s original collection, sampled in PV.

### Lectotype

Lectotype of *B. plebejus* Bonaparte: Collection of the Academy of Natural Sciences of Philadelphia No. ANSP 6183, from Lake Como.

### Paralectotypes

5 paralectotypes of *B. plebejus* Bonaparte: Collection of the Academy of Natural Sciences of Philadelphia No. ANSP 6184–6188, from Lake Como.

### Type locality

Lake Como.

### Distribution

PV

### Etymology

The specific name *plebejus* is the Latin word for plebeian, meaning common.

### Diagnosis

Due to overlapping, meristic characters have low or no reliability for morphological discrimination from other species; therefore, the diagnosis is mainly based on genetic differences in mitochondrial markers (*cytb* and D-loop).

### Morphology

Count and measurement are given in S8; last unbranched ray of the dorsal fin moderately ossified and with fine serration; ossification reduced in the distal portion of the ray, serration tend to become less evident as the fish becomes older.

### Remarks

*B. plebejus* Bonaparte 1839 is a fluvio-lacustrine barbel species perfectly matching the PV, parapatric to *B. tyberinus* (TL range) and to the new species proposed in this work (TSAAC range). Originally mentioned (as *nomen nudum*) under the name of *B. plebejus* by Cuvier [112], the first valid description of this taxon, *B. plebejus* Bonaparte, 1839 [91], is exclusively based on type specimens from PV; hence, no revision or emendation is needed.

## Appendix 2

### Review of the original description of *Barbus tyberinus*

Family Cyprinidae Rafinesque 1815

Subfamily Barbinae Bleeker 1859

Genus *Barbus* Cuvier and Cloquet 1816

*Barbus tyberinus* Bonaparte 1839

### Synonymy

*Barbus fluviatilis tyberinus* [91]: fasc. 25, pl. 110 fig. 3 (Tiber River near Rome); *Barbus eques* [91]: fasc. 25 pl. 110 fig. 2 (partim: River Arno in Tuscany Region); Valenciennes in [103]: 141 (Florence); *Barbus canalii* Valenciennes in [103]: 143–144 (Topino River, misspelled Topico); *Barbus fucini* [113]: folio 79, pl 11, figs 1-7 (lake Fucino) [113]; sensu Bianco [23]: 463 (partim: TL); *Barbus fluviatilis plebejus* (non Bonaparte 1839) [92] (partim: TL); *Barbus plebejus* (non Bonaparte 1839), [93]: 72–77 (partim: TL); [94]: 11 (partim: TL); [95]: 43 (partim: Castelnuovo in Garfagnana, Arno River at Florence, Casentino region); [114]: 28 (Rome province); [96]: 42–43 (partim: TL); [97]: 40–41 (partim: TL); [98]: 274–275 (partim: TL); [35]: 198–202 (partim: TL); *Barbus Barbus plebejus* (non Bonaparte 1839) [99]: 172 (partim: TL); [100]: 19 (partim: TL); [101]: 274–275 (partim: TL); *Barbus tyberinus* (non Bonaparte 1839) [28]: 313–318, figs. 4b, 6–7 (Partim: TL); [102]: 50–51 (Partim: TL); [14]: 234 (Partim: TL); [34]: 427 (Partim: TL).

### Type specimens

The type specimens defined by Bianco [34] within syntypes of Bonaparte’s and Costa’s original collections, sampled in TL.

### Lectotype

Lectotype of *B. fluviatilis tyberinus*: Collection of the Academy of Natural Sciences of Philadelphia No. ANSP 6152 (Bonaparte’s original number 426), from river Tevere. 122 mm SL (166 mm TL); LL 55; 15.5 row of scales above and 9.5 below LL; 26 circumpeduncular scales; D 8; 8 (6 + 2) gill rakers.

### Paralectotypes

27 paralectotypes of *B. fluviatilis tyberinus*: Collection of the Academy of Natural Sciences of Philadelphia No. ANSP 6153–6179, from river Tevere. 50–188 mm SL; 47–61 LL; 12.5–15.5 row of scales above and 7.5–9.5 below LL; 24–28 circumpeduncular scales; D 8; 8–10 (6–8+ 1–3) gill rakers. Lectotype of *B. canalii*. Muséum national d’Histoire Naturelle in Paris No. MNHN 1412; from river Topino. 146 mm SL; 53 LL; 12.5 row of scales above and 8.5 scales below LL; 24 circumpeduncular scales; 8 (7 + 1) gill rakers. 5 paralectotypes of *B. eques* Bonaparte: Collection of the Academy of Natural Sciences of Philadelphia No. ANSP 6144, 6146–6149, from river Arno.

### Type locality

River Tevere near Rome.

### Distribution

TL

### Etymology

The specific name *tyberinus* is derived from the name of the River Tevere, the type locality.

### Diagnosis

Same considerations as for *B. plebejus*.

### Morphology

Count and measurement are given in S8; last unbranched ray of the dorsal fin poorly ossified and with fine serration; ossification reduced in the distal portion of the ray, serration tend to become undistinguished as the fish becomes older.

### Remarks

*B. tyberinus* Bonaparte 1839 is a fluvio-lacustrine barbel species perfectly matching the TL, parapatric to *B. plebejus* (PV range) and to the new species proposed in this work (TSAAC range); the presence of the species in Liri-Garigliano basin, the northernmost watershed of Apulia-Campania district at the boundary with Tuscany-Latium district is likely to be naturally due to the geomorphological phenomena.

Conversely, on the basis of morphological analysis [28, 34] *B. tyberinus* was considered including every non-PV fluvio-lacustrine populations; therefore, its range was assumed covering also AC.

The first valid description of this taxon, *B. fluviatilis tyberinus* Bonaparte 1839 is based on type specimens all sampled in TL as individuated by Bianco [28], therefore the original description of Bonaparte must be considered correct; anyway, being not a subspecies of *B. fluviatilis*, rather a different species, Bianco [28] identified as valid the combination *B. tyberinus* Bonaparte 1839. Correctly, following article 51.3.2. of ICZN, no parenthesis was added around author’s name and date. Moreover, this combination is also a synonym of *B. eques* Valenciennes in Cuvier and Valenciennes 1842, of *B. canalii* Valenciennes in Cuvier and Valenciennes 1842 and *B. fucini* Costa 1853.

## Abbreviations

PV: Padano-Venetian district
TL: Tuscany-Latium district
AC: Apulia-Campania district
NAAC: Northern Adriatic part of Apulia-Campania district
TSAAC: Tyrrhenian and southern Adriatic part of Apulia-Campania district
DAN: Danubian district
*cytb*: Cytochrome b
*trnP*: tRNA-Pro
BP: Bootstrap proportion
PP: Posterior probability
UF-Boot: Ultrafast bootstrap support value
P: Prior intraspecific distance
