# Correction: Mitochondrial phylogeny and taxonomic revision of Italian and slovenian fluvio-lacustrine barbels, Barbus sp. (Cypriniformes, Cyprinidae)

**DOI:** 10.1186/s40850-023-00167-8

**Published:** 2023-06-02

**Authors:** Giovanni Rossi, Federico Plazzi, Gianluca Zuffi, Andrea Marchi, Salvatore De Bonis, Marco Valli, Petra Marinšek, Rosanna Falconi

**Affiliations:** 1 Department of Biological, Geological and Environmental Sciences, University of Bologna, Bologna, Italy; 2 Hydrosynergy SC, San Lazzaro di Savena, Italy; 3 Sezione di Bologna, Arpae Emilia-Romagna, Bologna, Italy; 4 Servizio Monitoraggio Risorse Idriche, Arpa Lazio, Rome, Italy; 5 Regione Emilia-Romagna, Servizio Attività Faunistico Venatorie e Pesca, Bologna, Italy; 6 Faculty of Natural Science and Mathematics, University of Maribor, Maribor, Slovenia; 7 Hochdorf Swiss Nutrition AG, Hochdorf, Switzerland

**Correction to: BMC Zoology (2021) 6:8**

10.1186/s40850-021-00073-x

Following publication of the original article [1], the link to the nomenclature act, which is made mandatory by the International Commission on Zoological Nomenclature (ICZN) for a species description to be valid, was accidentally removed.

We have therefore added the link and changed the section ‘Nomenclature acts’ from:

“The electronic edition of this article conforms to the requirements of the amended International Code of Zoological Nomenclature, and hence the new name contained herein is available under that Code from the electronic edition of this article.”

to:

“The electronic version of this article is considered a published work according to the International Commission on Zoological Nomenclature (ICZN). Therefore, the new name contained in the electronic version is officially published under the ICZN code (see articles 8.5–8.6) alone. The work and its content have also been registered in ZooBank (http://zoobank.org/), the online registration system for the ICZN and can be accessed under http://zoobank.org/urn:lsid:zoobank.org:pub:BE76B7A1-8FF7-4903-A1F7-9D52D03EAD83.”

The original article [1] has been corrected.
