# Supplementary material for: Mitochondrial phylogeny and taxonomic revision of Italian and Slovenian fluvio-lacustrine barbels, Barbus sp. (Cypriniformes, Cyprinidae)
Source: BMC Zool. 2021 Apr 21;6:8. doi: 10.1186/s40850-021-00073-x (PMC10127354; doi:10.1186/s40850-021-00073-x)
Supplement: Supplementary file 1 — Additional file 1. Sampling sites distribution, along with coordinates in EPSG 32632 reference systems. [file 40850_2021_73_MOESM1_ESM.pdf]

Additional file 1. Sampling sites distribution, along with coordinates in EPSG 32632 reference systems.

Ichthyogeographic district[a]	Main watershed	River	Altitude	Coordinates		Country[b]	Toponym
			(m a.s.l).	X	Y		
AC	Alento	Alento	12	1024103	4468551	ITA	Poggio della Fornace
AC	Bussento	Bussento	14	1054157	4458347	ITA	Molino di Santa Lucia
AC	Candelaro	Celone	575	1017375	4594280	ITA	Fosso Calizzano
AC	Cervaro	Cervaro	393	1022706	4583826	ITA	Pian di Bartolo
AC	Fortore	Fortore	27	1017360	4644341	ITA	Velina
AC	Lambro	Lambro	15	1036851	4451873	ITA	Figline Valdarno
AC	Liri-Garigliano	Liri	330	878260	4638622	ITA	Valpiana
AC	Mingardo	Mingardo	15	1039731	4453245	ITA	Vanganel
AC	Ofanto	Fiumara di Atella	340	1052856	4546315	ITA	il Calvario
AC	Ofanto	Ofanto	223	1049790	4566857	ITA	Molino Nuovo
AC	Picentino	Picentino	56	996734	4519647	ITA	Montelattaia
AC	Sele	Sele	49	1021369	4513421	ITA	Balzette
DAN	Danubio	Brestanica	184	1000905	5113340	SLO	S. Giovanni-Lago
DAN	Danubio	Krka	143	1001240	5099529	SLO	Vocabolo Chigualtieri
DAN	Danubio	Sava	134	1012473	5103427	SLO	Mulino della Badia
DAN	Danubio	Sava	152	1005445	5107313	SLO	Secchiano
DAN	Danubio	Sotla	158	1019091	5117519	SLO	Brestanica
DAN	Danubio	Sotla	149	1019047	5114645	SLO	C. Santa Chiara
PV	Badaševica	Badaševica	20	873044	5051768	SLO	Ponte di Macchiascandona
PV	Dragonja	Rokava	85	871672	5046745	SLO	La Cooperativa
PV	Fiumi Uniti	Bidente di Campigna	550	723205	4864132	ITA	Sovana
PV	Fiumi Uniti	Bidente di Celle	545	723187	4865182	ITA	Ponticello
PV	Fiumi Uniti	Montone	186	727828	4884241	ITA	Confluenza Fosso Cadone (Santa Fiora)

PV	Foglia	Foglia	85	793808	4855105	ITA	Ponte SP della Sgrilla
PV	Marecchia	Marecchia	101	773181	4875807	ITA	Fosso della Pietra
PV	Metauro	Biscubio	455	777697	4829795	ITA	Celle San Vito
PV	Metauro	Bosso	305	792225	4828182	ITA	Ischia
PV	Metauro	Certano	435	785309	4822562	ITA	Piano di Cornia
PV	Metauro	Meta	595	762595	4834684	ITA	C. la Fornacina
PV	Po	Baganza	790	579027	4928485	ITA	Galleo
PV	Po	Panaro	111	659132	4926082	ITA	Montecalvo in Foglia
PV	Po	Secchia	99	640781	4935633	ITA	Palinuro
PV	Po	Secchia	143	632835	4925147	ITA	Capannelle
PV	Po	Secchiello	505	617239	4912159	ITA	Balsorano Nuovo
PV	Reno	Borro Diaterna	520	688554	4890518	ITA	Monte Caibaldini
PV	Reno	Idice	78	693073	4921730	ITA	Petriolo
PV	Reno	Reno	240	664231	4899478	ITA	Madonna del Carmine
PV	Reno	Santerno	14	724054	4919242	ITA	Castel dei Britti
PV	Reno	Silla	365	655947	4894173	ITA	Ponte Verucchio
PV	Reno	Sillaro	31	712916	4922006	ITA	Podere la Ciaia
PV	Reno	Sintria	166	715124	4900451	ITA	P. Capanno
PV	Savio	Savio	355	743013	4863685	ITA	Brod v Podbocju
PV	Soča-Isonzo	Močilnik	104	884423	5087658	SLO	Ponte SP di Sovana (P. San Vincenzo)
PV	Soča-Isonzo	Soča	84	856857	5110453	SLO	Lamoli
PV	Tordino	Tordino	260	883688	4732232	ITA	Centola
PV	Tordino	Tordino	124	893143	4737787	ITA	farm near Gradišce pri Vipavi
PV	Tronto	Tronto	39	887923	4755975	ITA	Molino Cavina
TL	Albegna	Albegna	298	705666	4734996	ITA	Fattoria Grancia
TL	Albegna	Albegna	260	704668	4733062	ITA	Casa del Cavallo/Garofano
TL	Albegna	Elsa	55	699360	4711302	ITA	Molino delle Caselle

TL	Albegna	Fiascone	200	701038	4729924	ITA	Bedogno
TL	Albegna	Patrignone	68	688479	4719804	ITA	Bracna Vas
TL	Arno	Arno	115	700104	4832917	ITA	Magliano in Toscana
TL	Arno	Sieve	83	696936	4850517	ITA	Madonna della Cona
TL	Arno	Sorcella	295	682344	4876625	ITA	San Nicola a Tordino
TL	Bruna	Acquabona	78	658114	4760467	ITA	il Calvario
TL	Bruna	Bai	153	674283	4763541	ITA	Campigliano
TL	Bruna	Bandinelle	47	670369	4756645	ITA	Riola di Vergato
TL	Bruna	Bruna	50	660623	4759925	ITA	Župancici
TL	Bruna	Bruna	7	663772	4741069	ITA	Cava di Breccia
TL	Cornia	Cornia	40	639455	4770341	ITA	nuclear plant of Krško
TL	Fiora	Calesine	219	716207	4726037	ITA	ponte Mordano-Bagnara
TL	Fiora	Fiora	200	714690	4726230	ITA	Oasi Persano
TL	Fiora	Fiora	525	711855	4744366	ITA	Pontassieve
TL	Magra	Caprio	210	573781	4910462	ITA	Catež ob Savi
TL	Ombrone	Acquaviva	98	675010	4748028	ITA	Rocchetta Scalo
TL	Ombrone	Farma	150	687216	4772189	ITA	Sassuolo
TL	Ombrone	Farma	338	671785	4771211	ITA	Muraglione
TL	Ombrone	Gretano	100	681558	4761328	ITA	Molino di Gaggio
TL	Ombrone	Lanzo	115	688004	4761650	ITA	Castel Guelfo/Case Fornace
TL	Ombrone	Melacciole	99	693228	4745888	ITA	Zattaglia
TL	Ombrone	Merse	109	691433	4772402	ITA	Deskle
TL	Ombrone	Ombrone	11	674483	4734243	ITA	il Bello di Vvetta
TL	Ombrone	Salica	31	675148	4742883	ITA	Piantrebbio
TL	Ombrone	Trasubbie	171	694426	4740600	ITA	Frazione Tronto
TL	Pecora	Aronna	154	651370	4763053	ITA	p. Prato al Bardo
TL	Pecora	Pecora	181	653024	4769165	ITA	Stara Vas

TL	Tevere	Teveriola	620	744847	4849645	ITA	Baccinello

[a] AC, Apulia-Campania district; PV, Padano-Venetian district; TL, Tuscany-Latium district; DAN, Danubian district.

[b] ITA, Italy; SLO, Slovenia.
