# Supplementary material for: Mitochondrial phylogeny and taxonomic revision of Italian and Slovenian fluvio-lacustrine barbels, Barbus sp. (Cypriniformes, Cyprinidae)
Source: BMC Zool. 2021 Apr 21;6:8. doi: 10.1186/s40850-021-00073-x (PMC10127354; doi:10.1186/s40850-021-00073-x)
Supplement: Supplementary file 2 — Additional file 2. Dichotomous key for barbels of Apennine Peninsula and Adriatic basin of Slovenia and Croatia [15]. [file 40850_2021_73_MOESM2_ESM.pdf]

Additional file 2. Dichotomous key for barbels of Apennine Peninsula and Adriatic basin of Slovenia and Croatia [15].

1a. Lower lip without median lobe or swollen pad; 17–22 gill rakers; last simple dorsal ray slender, not serrated posteriorly in adults, occasionally with a few small, widely set serrae in individuals smaller than about 150 mm SL.

Luciobarbus graellsii

1b. Lower lip with median lobe or swollen pad; 10–14 gill rakers; last simple dorsal ray serrated posteriorly or not.

2

2a. Median part of lower lip produced posteriorly into a lobe.

3

2b. Lower lip thick, with a median swollen pad.

5

3a. Tip of dorsal pointed; posterior margin of dorsal concave; last simple ray finely serrated posteriorly; 58–77 + 3–4 lateral line scales; sides finely dotted in individuals larger than 150 mm SL; paired fins orange to grey without conspicuous spots.

Barbus plebejus

3b. Tip of dorsal rounded; posterior margin of dorsal straight or convex; last simple dorsal ray not serrated posteriorly; 36–57 + 2–4 lateral line scales; back, sides and fins speckled with large, irregular black dots.

4

4a. 36–47 + 2–3 lateral line scales.

Barbus caninus

4b. 49–57 + 3–4 lateral line scales.

Barbus balcanicus

5a. Posterior margin of dorsal strongly concave; last simple dorsal ray spinous, with strong serrae along entire posterior edge; posterior part of scales (art least on caudal peduncle) pointed.

Barbus barbus

5b. Posterior margin of dorsal slightly concave; last simple dorsal ray feebly hardened, finely serrated along posterior edge in small individuals or serrae absent in individuals larger than about 150–200 mm SL; posterior part of scales (at least on caudal peduncle) rounded.

Barbus tyberinus
