# Supplementary material for: Mitochondrial phylogeny and taxonomic revision of Italian and Slovenian fluvio-lacustrine barbels, Barbus sp. (Cypriniformes, Cyprinidae)
Source: BMC Zool. 2021 Apr 21;6:8. doi: 10.1186/s40850-021-00073-x (PMC10127354; doi:10.1186/s40850-021-00073-x)
Supplement: Supplementary file 3 — Additional file 3. GenBank Accession Numbers and geographical abundancies of sequences used for single-marker minimum spanning network computations (see Additional file 6). TL, Tuscany-Latium district; PV, Padano-Venetian district; NAAC, northernmost-Adriatic part of Apulia-Campania district; TSAAC, Tyrrhenian and southernmost-Adriatic parts of Apulia-Campania district; DAN, Danubian district; AccNum, GenBank Accession Number. [file 40850_2021_73_MOESM3_ESM.pdf]

Additional file 3. GenBank Accession Numbers and geographical abundancies of sequences used for single-marker minimum spanning network computations (see Additional file 6).

	TL	PV	NAAC	TSAAC	DAN	AccNum		TL	PV	NAAC	TSAAC	DAN	AccNum
1_1	0	61	0	0	0	KC818238	Bplebejus1	2	0	0	0	0	MG717942
04_25	0	1	0	0	0	KC818239	Bplebejus2	1	2	0	0	0	MG717943
04_26	0	1	0	0	0	KC818240	Bplebejus3	0	1	0	0	0	MG717944
1_2	0	4	0	0	0	KC818241	Bplebejus4	1	0	0	0	0	MG717945
1_3	0	1	0	0	0	KC818242	Bplebejus5	7	1	0	0	0	MG717946
2_1	0	3	0	0	0	KF963315	Bplebejus6	1	0	0	0	0	MG717947
2_3	0	1	0	0	0	KC818243	Bplebejus7	4	0	0	0	0	MG717948
2_5	0	3	0	0	0	KC818244	Bplebejus8	1	1	0	0	0	MG717949
2_9	0	1	0	0	0	KC818245	Bplebejus9	1	0	0	0	0	MG717950
1C	20	18	0	0	4	KF963316	Bplebejus10	0	2	0	0	0	MG717951
3C	6	1	0	0	0	KC818255	Bplebejus11	16	19	0	0	0	MG717952
9a1	2	5	0	0	0	KC818247	Bplebejus12	13	0	0	0	0	MG717953
3_1	19	47	0	0	0	KC818248	Bplebejus13	2	0	0	0	0	MG717954
7_2	0	1	0	0	0	KC818249	Bplebejus14	11	0	0	0	0	MG717955
F1_1	0	21	0	0	0	KC818250	Bplebejus15	4	1	0	0	0	MG717956
F2_1	0	14	0	0	0	KC818251	Bplebejus16	18	0	0	0	0	MG717957
t3	0	2	0	0	1	KC818252	Bplebejus17	28	2	0	0	0	MG717958
terd5	0	1	0	0	0	KC818253	Bplebejus18	1	0	0	0	0	MG717959
8_10	0	1	0	0	0	KC818246	Bplebejus19	59	2	0	0	0	MG717960
aln1	18	0	0	0	0	KC818254	Bplebejus20	3	0	0	0	0	MG717961
2lb	1	0	0	0	0	KC818256	Bplebejus21	0	3	0	0	0	MG717962
aln5	21	0	0	0	0	KC818257	Bplebejus22	6	0	0	0	0	MG717963
1_VF	1	0	0	0	0	KC818258	Bplebejus23	5	0	0	0	0	MG717964

cerf18	1	0	0	0	0	KC818259	Bplebejus24	4	0	0	0	0	MG717965
ALN7	2	0	0	0	0	KC818260	Bplebejus25	9	0	0	0	0	MG717966
ALN8	2	0	0	0	0	KC818261	Bplebejus26	1	0	0	0	0	MG717967
ALN10	1	0	0	0	0	KC818262	Bplebejus27	1	0	0	0	0	MG717968
BT4	1	0	0	0	0	KC818263	Bplebejus28	1	4	0	0	0	MG717969
BT5	2	0	0	0	0	KC818264	Bplebejus29	2	0	0	0	0	MG717970
Bbarbus	0	32	0	0	14	AY331019	Bplebejus30	1	0	0	0	0	MG717971
Bplebejus	0	2	0	0	0	AY004750	Bplebejus31	2	0	0	0	0	MG717972
Btyberinus	1	0	0	0	0	AF397300	Bplebejus32	1	0	0	0	0	MG717973
Bplebejus1	0	4	0	0	0	KC465936	Bplebejus33	0	1	0	0	0	MG717974
Bplebejus2	0	1	0	0	0	KC465940	Bplebejus34	2	0	0	0	0	MG717975
Bplebejus3	0	2	0	0	0	KC465947	Bplebejus35	1	2	0	0	0	MG717976
Bplebejus4	0	2	0	0	0	KC465941	Bplebejus36	1	0	0	0	0	MG717977
Bplebejus5	0	3	0	0	0	KC465937	Bplebejus37	3	0	0	0	0	MG717978
Bplebejus6	0	2	0	0	0	KC465933	Bplebejus38	2	0	0	0	0	MG717979
Bplebejus7	0	9	0	0	0	KC465934	Bplebejus39	5	41	0	0	0	MG717980
Bplebejus8	0	324	0	0	0	KC465928	Bplebejus40	3	72	0	0	0	MG717981
Bplebejus9	0	1	0	0	0	KC465932	Bplebejus41	0	17	0	0	0	MG717982
Bplebejus10	0	2	0	0	0	KC465929	Bplebejus42	0	28	0	0	0	MG717983
Bplebejus11	0	1	0	0	0	KC465930	Bplebejus43	1	17	0	0	0	MG717984
Bplebejus12	0	1	0	0	0	KC465946	Bplebejus44	0	28	0	0	0	MG717985
Bplebejus13	0	1	0	0	0	KC465943	Bplebejus45	3	11	0	0	0	MG717986
Bplebejus14	0	1	0	0	0	KC465939	Bplebejus46	0	18	0	0	0	MG717987
Bplebejus15	0	1	0	0	0	KC465944	Bplebejus47	0	11	0	0	0	MG717988
Bplebejus16	0	1	0	0	0	KC465938	Bplebejus48	8	2	0	0	0	MG717989
Bplebejus17	0	7	0	0	0	KC465942	Bplebejus49	0	7	0	0	0	MG717990
Bplebejus18	0	4	0	0	0	KC465931	Bplebejus50	0	6	0	0	0	MG717991

Bplebejus19	0	39	0	0	0	KC465935	Bplebejus51	0	6	0	0	0	MG717992
Bplebejus20	0	1	0	0	0	KC465948	Bplebejus52	0	4	0	0	0	MG717993
Bplebejus21	0	1	0	0	0	KC465949	Bplebejus53	1	1	0	0	0	MG717994
Bbarbus1	0	9	0	0	0	KC465920	Bplebejus54	1	2	0	0	0	MG717995
Bbarbus2	0	1	0	0	0	AY331023	Bplebejus55	1	1	0	0	0	MG717996
Bbarbus3	0	3	0	0	0	KC465924	Bplebejus56	0	1	0	0	0	MG717997
Bbarbus4	0	71	0	0	6	AY331017	Bplebejus57	0	1	0	0	0	MG717998
Bbarbus5	0	105	0	0	4	AY331020	Bplebejus58	0	5	0	0	0	MG717999
Bbarbus6	0	1	0	0	0	KC465926	Bplebejus59	0	7	0	0	0	MG718000
Bbarbus7	0	1	0	0	0	AY331018	Bplebejus60	0	2	0	0	0	MG718001
Bbarbus8	0	3	0	0	0	KC465927	Bplebejus61	1	1	0	0	0	MG718002
Bbarbus9	0	0	0	0	1	KC465925	Bplebejus62	0	1	0	0	0	MG718003
Bbarbus10	0	1	0	0	0	AY331024	Bplebejus63	0	3	0	0	0	MG718004
Bbarbus11	0	4	0	0	0	AY331021	Bplebejus64	0	1	0	0	0	MG718005
Bbarbus12	0	13	0	0	0	KC465918	Bplebejus65	0	2	0	0	0	MG718006
Bbarbus13	0	1	0	0	0	AY331022	Bplebejus66	0	1	0	0	0	MG718007
Bbarbus14	0	1	0	0	0	KC465922	Bplebejus67	0	1	0	0	0	MG718008
BaBaBAR3	0	1	0	0	0	KF923537	Bplebejus68	0	6	0	0	0	MG718009
BaBaBAR4	0	1	0	0	0	KF923538	Bplebejus69	0	1	0	0	0	MG718010
BaPlPLE1	0	3	0	0	0	KF923539	Bplebejus70	0	1	0	0	0	MG718011
BaPlPLE2	0	18	0	0	0	KF923540	Bplebejus73	0	0	21	0	0	MG718014
BaPlPLE3	0	1	0	0	0	KF923541	Bplebejus74	0	0	1	0	0	MG718015
BaPlPLE4	0	1	0	0	0	KF923542	Bplebejus75	0	0	1	0	0	MG718016
Bplebejus22	0	0	13	0	0	MK728816	Bplebejus76	0	0	1	0	0	MG718017
Bplebejus23	0	0	0	80	0	MK728817	Bplebejus77	0	0	1	0	0	MG718018
Bplebejus25	0	0	0	27	0	MK728819	Bplebejus78	0	0	34	0	0	MK728797
Bplebejus26	0	0	0	1	0	MK728820	Bplebejus79	0	0	13	0	0	MK728798

Bplebejus27	0	0	0	13	0	MK728821	Bplebejus80	0	0	11	0	0	MK728799
Bplebejus28	0	0	46	0	0	MG718025	Bplebejus81	0	0	1	0	0	MK728800
Bplebejus29	0	0	1	0	0	MG718026	Bplebejus82	0	0	1	0	0	MK728801
AQ83Liri330BA	0	0	0	1	0	MG495773	Bplebejus83	0	0	0	49	0	MK728802
AQ84Lir330BA	0	0	0	1	0	MG495774	Bplebejus84	0	0	0	1	0	MK728803
AQ90Lir330BA	0	0	0	1	0	MG495775	Bplebejus85	0	0	0	1	0	MK728804
BavaG02	0	1	0	0	0	MG495776	Bplebejus86	0	0	0	1	0	MK728805
BavaG04	0	1	0	0	0	MG495777	Bplebejus87	0	0	0	4	0	MK728806
BO121Sil365BA	0	1	0	0	0	MG495778	Bplebejus88	0	0	0	1	0	MK728807
BO128Sil365BA	0	1	0	0	0	MG495779	Bplebejus89	0	0	0	22	0	MK728808
BO12Idc78BA	0	1	0	0	0	MG495780	Bplebejus90	0	0	0	13	0	MK728809
BO14Idc78BA	0	1	0	0	0	MG495781	Bplebejus91	0	0	0	13	0	MK728810
BO26Slr31BA	0	1	0	0	0	MG495782	Bplebejus92	0	0	0	4	0	MK728811
BO30Snt14BA	0	1	0	0	0	MG495783	Bplebejus93	0	0	0	3	0	MK728812
BO31Snt14BA	0	1	0	0	0	MG495784	Bplebejus94	0	0	0	3	0	MK728813
BO56Ren250BA	0	1	0	0	0	MG495785	Bplebejus95	0	0	0	1	0	MK728814
FC202Svo350BA	0	1	0	0	0	MG495790	Bplebejus96	0	0	0	5	0	MK728815
FC203Svo350BA	0	1	0	0	0	MG495791	AQ83Liri330BA	0	0	0	1	0	MG495623
FC555Mon196BA	0	1	0	0	0	MG495794	AQ84Lir330BA	0	0	0	1	0	MG495624
FC556Mon196BA	0	1	0	0	0	MG495795	AQ90Lir330BA	0	0	0	1	0	MG495625
FC68Tva620BA	1	0	0	0	0	MG495796	BavaG02	0	1	0	0	0	MG495626
FG10For197BA	0	0	0	1	0	MG495797	BavaG04	0	1	0	0	0	MG495627
FG20Crv350BA	0	0	0	1	0	MG495798	BO121Sil365BA	0	1	0	0	0	MG495628
FG27Crv350BA	0	0	0	1	0	MG495799	BO128Sil365BA	0	1	0	0	0	MG495629
FG29Crv350BA	0	0	0	1	0	MG495800	BO12Idc78BA	0	1	0	0	0	MG495630
FG5For197BA	0	0	0	1	0	MG495801	BO14Idc78BA	0	1	0	0	0	MG495631
FG74Ofn222BA	0	0	0	1	0	MG495802	BO26Slr31BA	0	1	0	0	0	MG495632

FG83Cal750BA	0	0	0	1	0	MG495803	BO30Snt14BA	0	1	0	0	0	MG495633
FI114Src295BA	1	0	0	0	0	MG495804	BO31Snt14BA	0	1	0	0	0	MG495634
FI21BoD520BA	0	1	0	0	0	MG495805	BO56Ren250BA	0	1	0	0	0	MG495635
FI339Siv83BA	1	0	0	0	0	MG495806	FC202Svo350BA	0	1	0	0	0	MG495640
FI492Arn115BA	1	0	0	0	0	MG495807	FC203Svo350BA	0	1	0	0	0	MG495641
GR1203Acv99BT	1	0	0	0	0	MG495808	FC555Mon196BA	0	1	0	0	0	MG495644
GR1210Acb78BA	1	0	0	0	0	MG495809	FC556Mon196BA	0	1	0	0	0	MG495645
GR1211Acb78BA	1	0	0	0	0	MG495810	FC68Tva620BA	1	0	0	0	0	MG495646
GR1229Frm340BT	1	0	0	0	0	MG495811	FG10For197BA	0	0	0	1	0	MG495647
GR1259Lnz116BA	1	0	0	0	0	MG495812	FG20Crv350BA	0	0	0	1	0	MG495648
GR1281Arn155BT	1	0	0	0	0	MG495813	FG27Crv350BA	0	0	0	1	0	MG495649
GR1282Arn155BT	1	0	0	0	0	MG495814	FG29Crv350BA	0	0	0	1	0	MG495650
GR1294Bnd49BT	1	0	0	0	0	MG495815	FG5For197BA	0	0	0	1	0	MG495651
GR1321Mle99BT	1	0	0	0	0	MG495816	FG74Ofn222BA	0	0	0	1	0	MG495652
GR1322Mle99BT	1	0	0	0	0	MG495817	FG83Cal750BA	0	0	0	1	0	MG495653
GR1323Mle99BT	1	0	0	0	0	MG495818	FI114Src295BA	1	0	0	0	0	MG495654
GR1331Tra171BT	1	0	0	0	0	MG495819	FI21BoD520BA	0	1	0	0	0	MG495655
GR1333Tra171BT	1	0	0	0	0	MG495820	FI339Siv83BA	1	0	0	0	0	MG495656
GR1395Cls229BA	1	0	0	0	0	MG495821	FI492Arn115BA	1	0	0	0	0	MG495657
GR1488Alb298BT	1	0	0	0	0	MG495822	GR1203Acv99BT	1	0	0	0	0	MG495658
GR1489Alb298BT	1	0	0	0	0	MG495823	GR1210Acb78BA	1	0	0	0	0	MG495659
GR1492Pcr181BT	1	0	0	0	0	MG495824	GR1211Acb78BA	1	0	0	0	0	MG495660
GR1493Pcr181BT	1	0	0	0	0	MG495825	GR1229Frm340BT	1	0	0	0	0	MG495661
GR15Brn50BT	1	0	0	0	0	MG495826	GR1259Lnz116BA	1	0	0	0	0	MG495662
GR202Frm150BA	1	0	0	0	0	MG495827	GR1281Arn155BT	1	0	0	0	0	MG495663
GR210Frm150BT	1	0	0	0	0	MG495828	GR1282Arn155BT	1	0	0	0	0	MG495664
GR277Cor40BT	1	0	0	0	0	MG495829	GR1294Bnd49BT	1	0	0	0	0	MG495665

GR30Brn50BT	1	0	0	0	0	MG495830	GR1321Mle99BT	1	0	0	0	0	MG495666
GR314Mrs109BT	1	0	0	0	0	MG495831	GR1322Mle99BT	1	0	0	0	0	MG495667
GR365Tpa68BA	1	0	0	0	0	MG495832	GR1323Mle99BT	1	0	0	0	0	MG495668
GR436Els55BA	1	0	0	0	0	MG495833	GR1331Tra171BT	1	0	0	0	0	MG495669
GR448Els55BA	1	0	0	0	0	MG495834	GR1333Tra171BT	1	0	0	0	0	MG495670
GR460Fio525BA	1	0	0	0	0	MG495835	GR1395Cls229BA	1	0	0	0	0	MG495671
GR490Fio200BA	1	0	0	0	0	MG495836	GR1488Alb298BT	1	0	0	0	0	MG495672
GR493Fio200Ba	1	0	0	0	0	MG495837	GR1489Alb298BT	1	0	0	0	0	MG495673
GR587Alb260BA	1	0	0	0	0	MG495840	GR1492Pcr181BT	1	0	0	0	0	MG495674
GR590Alb260BA	1	0	0	0	0	MG495841	GR1493Pcr181BT	1	0	0	0	0	MG495675
GR591Alb260BA	1	0	0	0	0	MG495842	GR15Brn50BT	1	0	0	0	0	MG495676
GR676Omb30BA	1	0	0	0	0	MG495844	GR202Frm150BA	1	0	0	0	0	MG495677
GR689ASO31BT	1	0	0	0	0	MG495845	GR210Frm150BT	1	0	0	0	0	MG495678
GR692ASO31BT	1	0	0	0	0	MG495846	GR277Cor40BT	1	0	0	0	0	MG495679
GR726Brn7BA	1	0	0	0	0	MG495847	GR30Brn50BT	1	0	0	0	0	MG495680
GR727Brn7BS	1	0	0	0	0	MG495848	GR314Mrs109BT	1	0	0	0	0	MG495681
GR729Gre100BT	1	0	0	0	0	MG495849	GR365Tpa68BA	1	0	0	0	0	MG495682
GR730Gre100BT	1	0	0	0	0	MG495850	GR436Els55BA	1	0	0	0	0	MG495683
GR801Bai153BT	1	0	0	0	0	MG495851	GR448Els55BA	1	0	0	0	0	MG495684
GR802Bai153BT	1	0	0	0	0	MG495852	GR460Fio525BA	1	0	0	0	0	MG495685
KrkaPO13	0	0	0	0	1	MG495853	GR490Fio200BA	1	0	0	0	0	MG495686
KrkaPO16	0	0	0	0	1	MG495854	GR493Fio200BA	1	0	0	0	0	MG495687
KrkaPO18	0	0	0	0	1	MG495855	GR587Alb260BA	1	0	0	0	0	MG495690
MO14Seca185BA	0	1	0	0	0	MG495856	GR590Alb260BA	1	0	0	0	0	MG495691
MO1Pan113BA	0	1	0	0	0	MG495857	GR591Alb260BA	1	0	0	0	0	MG495692
MO24Seco512BA	0	1	0	0	0	MG495858	GR676Omb30BA	1	0	0	0	0	MG495694
MO45Seca97BA	0	1	0	0	0	MG495859	GR689ASO31BT	1	0	0	0	0	MG495695

MO5Pan113BA	0	1	0	0	0	MG495860	GR692ASO31BT	1	0	0	0	0	MG495696
MoajG05	0	1	0	0	0	MG495861	GR726Brn7BA	1	0	0	0	0	MG495697
MOAJG06	0	1	0	0	0	MG495862	GR727Brn7BS	1	0	0	0	0	MG495698
MoajG07	0	1	0	0	0	MG495863	GR729Gre100BT	1	0	0	0	0	MG495699
MoajG09	0	1	0	0	0	MG495864	GR730Gre100BT	1	0	0	0	0	MG495700
MS26CprBA	1	0	0	0	0	MG495866	GR801Bai153BT	1	0	0	0	0	MG495701
RA606Sin150BA	0	1	0	0	0	MG495869	GR802Bai153BT	1	0	0	0	0	MG495702
RN10Mar101BB	0	1	0	0	0	MG495870	KrkaPO13	0	0	0	0	1	MG495703
RN8Mar101BA	0	1	0	0	0	MG495871	KrkaPO16	0	0	0	0	1	MG495704
RN9Mar101BA	0	1	0	0	0	MG495872	KrkaPO18	0	0	0	0	1	MG495705
RoG06	0	1	0	0	0	MG495873	MO14Seca185BA	0	1	0	0	0	MG495706
SA11Sel70BA	0	0	0	1	0	MG495874	MO1Pan113BA	0	1	0	0	0	MG495707
SA144BstBA	0	0	0	1	0	MG495875	MO24Seco512BA	0	1	0	0	0	MG495708
SA199LmbBA	0	0	0	1	0	MG495876	MO45Seca97BA	0	1	0	0	0	MG495709
SA170MnrBA	0	0	0	1	0	MG495877	MO5Pan113BA	0	1	0	0	0	MG495710
SA54AlnBA	0	0	0	1	0	MG495878	MoajG05	0	1	0	0	0	MG495711
SACA65	0	0	0	0	1	MG495879	MOAJG06	0	1	0	0	0	MG495712
SAKR127	0	0	0	0	1	MG495880	MoajG07	0	1	0	0	0	MG495713
SodeG06	0	1	0	0	0	MG495881	MoajG09	0	1	0	0	0	MG495714
SODEG08	0	1	0	0	0	MG495882	MS26CprBA	1	0	0	0	0	MG495716
SodeG09	0	1	0	0	0	MG495883	RA606Sin150BA	0	1	0	0	0	MG495719
SodeG10	0	1	0	0	0	MG495884	RN10Mar101BB	0	1	0	0	0	MG495720
SOTLA30	0	0	0	0	1	MG495886	RN8Mar101BA	0	1	0	0	0	MG495721
SOTLA4	0	0	0	0	1	MG495888	RN9Mar101BA	0	1	0	0	0	MG495722
TE1Tro40BA	0	1	0	0	0	MG495889	RoG06	0	1	0	0	0	MG495723
TE40Tor124BA	0	1	0	0	0	MG495890	SA11Sel70BA	0	0	0	1	0	MG495724
TE41Tor124BA	0	1	0	0	0	MG495891	SA144BstBA	0	0	0	1	0	MG495725

TE60Tor260BA	0	1	0	0	0	MG495892	SA170MnrBA	0	0	0	1	0	MG495726
106	0	0	0	1	0	MG495893	SA199LmbBA	0	0	0	1	0	MG495727
108	0	0	0	1	0	MG495894	SA54AlnBA	0	0	0	1	0	MG495728
112	0	0	0	1	0	MG495895	SACA65	0	0	0	0	1	MG495729
116	0	0	0	1	0	MG495896	SAKR127	0	0	0	0	1	MG495730
122	0	0	0	1	0	MG495897	SodeG06	0	1	0	0	0	MG495731
123	0	0	0	1	0	MG495898	SODEG08	0	1	0	0	0	MG495732
124	0	0	0	1	0	MG495899	SodeG09	0	1	0	0	0	MG495733
125	0	0	0	1	0	MG495900	SodeG10	0	1	0	0	0	MG495734
PU136Bsc455BA	0	1	0	0	0	MG495901	SOTLA30	0	0	0	0	1	MG495736
PU186Bss305BA	0	1	0	0	0	MG495902	SOTLA4	0	0	0	0	1	MG495738
PU187Bss305BA	0	1	0	0	0	MG495903	TE1Tro40BA	0	1	0	0	0	MG495739
PU102Crt435BA	0	1	0	0	0	MG495904	TE40Tor124BA	0	1	0	0	0	MG495740
PU103Crt435BA	0	1	0	0	0	MG495905	TE41Tor124BA	0	1	0	0	0	MG495741
PU68Fgl85BA	0	1	0	0	0	MG495906	TE60Tor260BA	0	1	0	0	0	MG495742
PU69Fgl85BA	0	1	0	0	0	MG495907	106	0	0	0	1	0	MG495743
PU70Fgl85BA	0	1	0	0	0	MG495908	108	0	0	0	1	0	MG495744
PU245Mta595BA	0	1	0	0	0	MG495909	112	0	0	0	1	0	MG495745
PU246Mta595BA	0	1	0	0	0	MG495910	116	0	0	0	1	0	MG495746
PU247Mta595BA	0	1	0	0	0	MG495911	122	0	0	0	1	0	MG495747
Ofanto_01-M115	0	0	0	1	0	MG495912	123	0	0	0	1	0	MG495748
Ofanto_02-M113	0	0	0	1	0	MG495913	124	0	0	0	1	0	MG495749
Ofanto_03-M118	0	0	0	1	0	MG495914	125	0	0	0	1	0	MG495750
Ofanto_04-M104	0	0	0	1	0	MG495915	PU136Bsc455BA	0	1	0	0	0	MG495751
Ofanto_05-M108	0	0	0	1	0	MG495916	PU186Bss305BA	0	1	0	0	0	MG495752
Ofanto_06-M119	0	0	0	1	0	MG495917	PU187Bss305BA	0	1	0	0	0	MG495753
Ofanto_07-M111	0	0	0	1	0	MG495918	PU102Crt435BA	0	1	0	0	0	MG495754

Ofanto_08-M106	0	0	0	1	0	MG495919
Ofanto_09-M114	0	0	0	1	0	MG495920
Ofanto_10-M116	0	0	0	1	0	MG495921
Ofanto_11-M117	0	0	0	1	0	MG495922

PU103Crt435BA	0	1	0	0	0	MG495755
PU68Fgl85BA	0	1	0	0	0	MG495756
PU69Fgl85BA	0	1	0	0	0	MG495757
PU70Fgl85BA	0	1	0	0	0	MG495758
PU245Mta595BA	0	1	0	0	0	MG495759
PU246Mta595BA	0	1	0	0	0	MG495760
PU247Mta595BA	0	1	0	0	0	MG495761
Ofanto_01-M115	0	0	0	1	0	MG495762
Ofanto_02-M113	0	0	0	1	0	MG495763
Ofanto_03-M118	0	0	0	1	0	MG495764
Ofanto_04-M104	0	0	0	1	0	MG495765
Ofanto_05-M108	0	0	0	1	0	MG495766
Ofanto_06-M119	0	0	0	1	0	MG495767
Ofanto_07-M111	0	0	0	1	0	MG495768
Ofanto_08-M106	0	0	0	1	0	MG495769
Ofanto_09-M114	0	0	0	1	0	MG495770
Ofanto_10-M116	0	0	0	1	0	MG495771
Ofanto_11-M117	0	0	0	1	0	MG495772
