# Supplementary material for: Mitochondrial phylogeny and taxonomic revision of Italian and Slovenian fluvio-lacustrine barbels, Barbus sp. (Cypriniformes, Cyprinidae)
Source: BMC Zool. 2021 Apr 21;6:8. doi: 10.1186/s40850-021-00073-x (PMC10127354; doi:10.1186/s40850-021-00073-x)
Supplement: Supplementary file 4 — Additional file 4. Dataset, taxonomy, river/watershed location, sampling coordinates in EPSG 32632 reference systems and GenBank Accession Numbers. [file 40850_2021_73_MOESM4_ESM.pdf]

Additional file 4. Dataset, taxonomy, river/watershed location, sampling coordinates in EPSG 32632 reference systems and GenBank Accession Numbers.

Specimen voucher	Species	Altitude (m a.s.l).	River	Main watershed	Ichthyogeographic district[a]	Country[b]	Coordinates		Accession Numbers	
							X	Y	*cytb*	*dloop*
AQ83Liri330BA	*Barbus oscensis*	330	Liri	Liri-Garigliano	AC	ITA	878260	4638622	MG495773	MG495623
AQ84Lir330BA	*Barbus tyberinus*	330	Liri	Liri-Garigliano	AC	ITA	878260	4638622	MG495774	MG495624
AQ90Lir330BA	*Barbus oscensis*	330	Liri	Liri-Garigliano	AC	ITA	878260	4638622	MG495775	MG495625
BavaG02	*Barbus plebejus*	20	Badaševica	Badaševica	PV	SLO	873044	5051768	MG495776	MG495626
BavaG04	*Barbus plebejus*	20	Badaševica	Badaševica	PV	SLO	873044	5051768	MG495777	MG495627
BO121Sil365BA	*Barbus plebejus*	365	Silla	Reno	PV	ITA	655947	4894173	MG495778	MG495628
BO128Sil365BA	*Barbus plebejus*	365	Silla	Reno	PV	ITA	655947	4894173	MG495779	MG495629
BO12Idc78BA	*Barbus plebejus*	78	Idice	Reno	PV	ITA	693073	4921730	MG495780	MG495630
BO14Idc78BA	*Barbus plebejus*	78	Idice	Reno	PV	ITA	693073	4921730	MG495781	MG495631
BO26Slr31BA	*Barbus plebejus*	31	Sillaro	Reno	PV	ITA	712916	4922006	MG495782	MG495632
BO30Snt14BA	*Barbus barbus*	14	Santerno	Reno	PV	ITA	724054	4919242	MG495783	MG495633
BO31Snt14BA	*Barbus barbus*	14	Santerno	Reno	PV	ITA	724054	4919242	MG495784	MG495634
BO56Ren250BA	*Barbus tyberinus*	250	Reno	Reno	PV	ITA	664231	4899478	MG495785	MG495635
BRE1	*Barbus balcanicus*	184	Brestanica	Danubio	DAN	SLO	1000905	5113340	MG495786	MG495636
BRE10	*Barbus balcanicus*	184	Brestanica	Danubio	DAN	SLO	1000905	5113340	MG495787	MG495637
BRE2	*Barbus balcanicus*	184	Brestanica	Danubio	DAN	SLO	1000905	5113340	MG495788	MG495638
BRE7	*Barbus balcanicus*	184	Brestanica	Danubio	DAN	SLO	1000905	5113340	MG495789	MG495639
FC202Svo350BA	*Barbus barbus*	350	Savio	Savio	PV	ITA	743013	4863685	MG495790	MG495640
FC203Svo350BA	*Barbus barbus*	350	Savio	Savio	PV	ITA	743013	4863685	MG495791	MG495641
FC530BdCe525BA	*Barbus caninus*	525	Bidente di Celle	Fiumi Uniti	PV	ITA	723187	4865182	MG495792	MG495642
FC533BdCa547BA	*Barbus caninus*	547	Bidente di Campigna	Fiumi Uniti	PV	ITA	723205	4864132	MG495793	MG495643

FC555Mon196BA	*Barbus barbus*	196	Montone	Fiumi Uniti	PV	ITA	727828	4884241	MG495794	MG495644
FC556Mon196BA	*Barbus barbus*	196	Montone	Fiumi Uniti	PV	ITA	727828	4884241	MG495795	MG495645
FC68Tva620BA	*Barbus tyberinus*	620	Teveriola	Tevere	TL	ITA	744847	4849645	MG495796	MG495646
FG10For197BA	*Barbus oscensis*	197	Fortore	Fortore	AC	ITA	997988	4614109	MG495797	MG495647
FG20Crv350BA	*Barbus oscensis*	350	Cervaro	Cervaro	AC	ITA	1022706	4583826	MG495798	MG495648
FG27Crv350BA	*Barbus oscensis*	350	Cervaro	Cervaro	AC	ITA	1022706	4583826	MG495799	MG495649
FG29Crv350BA	*Barbus oscensis*	350	Cervaro	Cervaro	AC	ITA	1022706	4583826	MG495800	MG495650
FG5For197BA	*Barbus oscensis*	197	Fortore	Fortore	AC	ITA	997988	4614109	MG495801	MG495651
FG74Ofn222BA	*Barbus oscensis*	222	Ofanto	Ofanto	AC	ITA	1049790	4566857	MG495802	MG495652
FG83Cal750BA	*Barbus oscensis*	750	Celone	Candelaro	AC	ITA	1017375	4594280	MG495803	MG495653
FI114Src295BA	*Barbus tyberinus*	295	Sorcella	Arno	TL	ITA	682344	4876625	MG495804	MG495654
FI21BoD520BA	*Barbus plebejus*	520	Borro Diaterna	Reno	PV	ITA	688554	4890518	MG495805	MG495655
FI339Siv83BA	*Barbus barbus*	83	Sieve	Arno	TL	ITA	696936	4850517	MG495806	MG495656
FI492Arn115BA	*Barbus barbus*	115	Arno	Arno	TL	ITA	700104	4832917	MG495807	MG495657
GR1203Acv99BT	*Barbus tyberinus*	99	Acquaviva	Ombrone	TL	ITA	675010	4748028	MG495808	MG495658
GR1210Acb78BA	*Barbus tyberinus*	78	Acquabona	Bruna	TL	ITA	658114	4760467	MG495809	MG495659
GR1211Acb78BA	*Barbus tyberinus*	78	Acquabona	Bruna	TL	ITA	658114	4760467	MG495810	MG495660
GR1229Frm340BT	*Barbus tyberinus*	340	Farma	Ombrone	TL	ITA	671785	4771211	MG495811	MG495661
GR1259Lnz116BA	*Barbus tyberinus*	115	Lanzo	Ombrone	TL	ITA	688004	4761650	MG495812	MG495662
GR1281Arn155BT	*Barbus tyberinus*	155	Aronna	Pecora	TL	ITA	651370	4763053	MG495813	MG495663
GR1282Arn155BT	*Barbus tyberinus*	155	Aronna	Pecora	TL	ITA	651370	4763053	MG495814	MG495664
GR1294Bnd49BT	*Barbus tyberinus*	49	Bandinelle	Bruna	TL	ITA	670369	4756645	MG495815	MG495665
GR1321Mle99BT	*Barbus tyberinus*	99	Melacciole	Ombrone	TL	ITA	693228	4745888	MG495816	MG495666
GR1322Mle99BT	*Barbus tyberinus*	99	Melacciole	Ombrone	TL	ITA	693228	4745888	MG495817	MG495667
GR1323Mle99BT	*Barbus plebejus*	99	Melacciole	Ombrone	TL	ITA	693228	4745888	MG495818	MG495668
GR1331Tra171BT	*Barbus tyberinus*	171	Trasubbie	Ombrone	TL	ITA	694426	4740600	MG495819	MG495669
GR1333Tra171BT	*Barbus tyberinus*	171	Trasubbie	Ombrone	TL	ITA	694426	4740600	MG495820	MG495670

GR1395Cls229BA	*Barbus barbus*	229	Calesine	Fiora	TL	ITA	716207	4726037	MG495821	MG495671
GR1488Alb298BT	*Barbus plebejus*	298	Albegna	Albegna	TL	ITA	705666	4734996	MG495822	MG495672
GR1489Alb298BT	*Barbus tyberinus*	298	Albegna	Albegna	TL	ITA	705666	4734996	MG495823	MG495673
GR1492Pcr181BT	*Barbus tyberinus*	181	Pecora	Pecora	TL	ITA	653024	4769165	MG495824	MG495674
GR1493Pcr181BT	*Barbus plebejus*	181	Pecora	Pecora	TL	ITA	653024	4769165	MG495825	MG495675
GR15Brn50BT	*Barbus tyberinus*	50	Bruna	Bruna	TL	ITA	660623	4759925	MG495826	MG495676
GR202Frm150BA	*Barbus plebejus*	150	Farma	Ombrone	TL	ITA	687216	4772189	MG495827	MG495677
GR210Frm150BT	*Barbus tyberinus*	150	Farma	Ombrone	TL	ITA	687216	4772189	MG495828	MG495678
GR277Cor40BT	*Barbus plebejus*	40	Cornia	Cornia	TL	ITA	639455	4770341	MG495829	MG495679
GR30Brn50BT	*Barbus tyberinus*	50	Bruna	Bruna	TL	ITA	660623	4759925	MG495830	MG495680
GR314Mrs109BT	*Barbus barbus*	109	Merse	Ombrone	TL	ITA	691433	4772402	MG495831	MG495681
GR365Tpa68BA	*Barbus tyberinus*	68	Patrignone	Albegna	TL	ITA	688479	4719804	MG495832	MG495682
GR436Els55BA	*Barbus barbus*	55	Elsa	Albegna	TL	ITA	699360	4711302	MG495833	MG495683
GR448Els55BA	*Barbus tyberinus*	55	Elsa	Albegna	TL	ITA	699360	4711302	MG495834	MG495684
GR460Fio525BA	*Barbus tyberinus*	525	Fiora	Fiora	TL	ITA	711855	4744366	MG495835	MG495685
GR490Fio200BA	*Barbus plebejus*	200	Fiora	Fiora	TL	ITA	714690	4726230	MG495836	MG495686
GR493Fio200BA	*Barbus tyberinus*	200	Fiora	Fiora	TL	ITA	714690	4726230	MG495837	MG495687
GR568Usi200BA	*Luciobarbus graellsii*	200	Fiascone	Albegna	TL	ITA	701038	4729924	MG495838	MG495688
GR570Usi200BA	*Luciobarbus graellsii*	200	Fiascone	Albegna	TL	ITA	701038	4729924	MG495839	MG495689
GR587Alb260BA	*Barbus tyberinus*	260	Albegna	Albegna	TL	ITA	704668	4733062	MG495840	MG495690
GR590Alb260BA	*Barbus tyberinus*	260	Albegna	Albegna	TL	ITA	704668	4733062	MG495841	MG495691
GR591Alb260BA	*Barbus plebejus*	260	Albegna	Albegna	TL	ITA	704668	4733062	MG495842	MG495692
GR633Omb11BA	*Luciobarbus graellsii*	11	Ombrone	Ombrone	TL	ITA	674483	4734243	MG495843	MG495693
GR676Omb30BA	*Barbus barbus*	30	Ombrone	Ombrone	TL	ITA	674483	4734243	MG495844	MG495694
GR689ASO31BT	*Barbus tyberinus*	31	Salica	Ombrone	TL	ITA	675148	4742883	MG495845	MG495695
GR692ASO31BT	*Barbus tyberinus*	31	Salica	Ombrone	TL	ITA	675148	4742883	MG495846	MG495696
GR726Brn7BA	*Barbus barbus*	7	Bruna	Bruna	TL	ITA	663772	4741069	MG495847	MG495697

GR727Brn7BS	*Barbus plebejus*	7	Bruna	Bruna	TL	ITA	663772	4741069	MG495848	MG495698
GR729Gre100BT	*Barbus tyberinus*	100	Gretano	Ombrone	TL	ITA	681558	4761328	MG495849	MG495699
GR730Gre100BT	*Barbus tyberinus*	100	Gretano	Ombrone	TL	ITA	681558	4761328	MG495850	MG495700
GR801Bai153BT	*Barbus tyberinus*	153	Bai	Bruna	TL	ITA	674283	4763541	MG495851	MG495701
GR802Bai153BT	*Barbus tyberinus*	153	Bai	Bruna	TL	ITA	674283	4763541	MG495852	MG495702
KrkaPO13	*Barbus barbus*	143	Krka	Danubio	DAN	Croatia	1001240	5099529	MG495853	MG495703
KrkaPO16	*Barbus barbus*	143	Krka	Danubio	DAN	Croatia	1001240	5099529	MG495854	MG495704
KrkaPO18	*Barbus barbus*	143	Krka	Danubio	DAN	Croatia	1001240	5099529	MG495855	MG495705
MO14Seca185BA	*Barbus plebejus*	185	Secchia	Po	PV	ITA	632835	4925147	MG495856	MG495706
MO1Pan113BA	*Barbus plebejus*	113	Panaro	Po	PV	ITA	659132	4926082	MG495857	MG495707
MO24Seco512BA	*Barbus plebejus*	512	Secchiello	Po	PV	ITA	617239	4912159	MG495858	MG495708
MO45Seca97BA	*Barbus plebejus*	97	Secchia	Po	PV	ITA	640781	4935633	MG495859	MG495709
MO5Pan113BA	*Barbus plebejus*	113	Panaro	Po	PV	ITA	659132	4926082	MG495860	MG495710
MoajG05	*Barbus plebejus*	104	Močilnik	Soča-Isonzo	PV	SLO	884423	5087658	MG495861	MG495711
MOAJG06	*Barbus plebejus*	104	Močilnik	Soča-Isonzo	PV	SLO	884423	5087658	MG495862	MG495712
MoajG07	*Barbus plebejus*	104	Močilnik	Soča-Isonzo	PV	SLO	884423	5087658	MG495863	MG495713
MoajG09	*Barbus plebejus*	104	Močilnik	Soča-Isonzo	PV	SLO	884423	5087658	MG495864	MG495714
MOAJM16	*Barbus balcanicus*	104	Močilnik	Soča-Isonzo	PV	SLO	884423	5087658	MG495865	MG495715
MS26CprBA	*Barbus tyberinus*	180	Caprio	Magra	TL	ITA	573781	4910462	MG495866	MG495716
PR15Bag780BABC	*Barbus caninus*	780	Baganza	Po	PV	ITA	579027	4928485	MG495867	MG495717
PR18Bag780BC	*Barbus caninus*	780	Baganza	Po	PV	ITA	579027	4928485	MG495868	MG495718
RA606Sin150BA	*Barbus plebejus*	150	Sintria	Reno	PV	ITA	715124	4900451	MG495869	MG495719
RN10Mar101BB	*Barbus barbus*	101	Marecchia	Marecchia	PV	ITA	773181	4875807	MG495870	MG495720
RN8Mar101BA	*Barbus plebejus*	101	Marecchia	Marecchia	PV	ITA	773181	4875807	MG495871	MG495721
RN9Mar101BA	*Barbus plebejus*	101	Marecchia	Marecchia	PV	ITA	773181	4875807	MG495872	MG495722
RoG06	*Barbus plebejus*	85	Rokava	Dragonja	PV	SLO	871672	5046745	MG495873	MG495723
SA11Sel70BA	*Barbus oscensis*	70	Sele	Sele	AC	ITA	1021369	4513421	MG495874	MG495724

SA144BstBA	*Barbus oscensis*	14	Bussento	Bussento	AC	ITA	1054157	4458347	MG495875	MG495725
SA199LmbBA	*Barbus oscensis*	15	Lambro	Lambro	AC	ITA	1036851	4451873	MG495876	MG495727
SA170MnrBA	*Barbus oscensis*	15	Mingardo	Mingardo	AC	ITA	1039731	4453245	MG495877	MG495726
SA54AlnBA	*Barbus oscensis*	12	Alento	Alento	AC	ITA	1024103	4468551	MG495878	MG495728
SACA65	*Barbus barbus*	134	Sava	Danubio	DAN	SLO	1012473	5103427	MG495879	MG495729
SAKR127	*Barbus barbus*	152	Sava	Danubio	DAN	SLO	1005445	5107313	MG495880	MG495730
SodeG06	*Barbus plebejus*	84	Soča	Soča-Isonzo	PV	SLO	856857	5110453	MG495881	MG495731
SODEG08	*Barbus plebejus*	84	Soča	Soča-Isonzo	PV	SLO	856857	5110453	MG495882	MG495732
SodeG09	*Barbus plebejus*	84	Soča	Soča-Isonzo	PV	SLO	856857	5110453	MG495883	MG495733
SodeG10	*Barbus plebejus*	84	Soča	Soča-Isonzo	PV	SLO	856857	5110453	MG495884	MG495734
SodeG13	*Barbus balcanicus*	84	Soča	Soča-Isonzo	PV	SLO	856857	5110453	MG495885	MG495735
SOTLA30	*Barbus barbus*	158	Sotla	Danubio	DAN	SLO	1019091	5117519	MG495886	MG495736
Sotla361	*Barbus balcanicus*	149	Sotla	Danubio	DAN	SLO	1019047	5114645	MG495887	MG495737
SOTLA4	*Barbus barbus*	158	Sotla	Danubio	DAN	SLO	1019091	5117519	MG495888	MG495738
TE1Tro40BA	*Barbus plebejus*	40	Tronto	Tronto	PV	ITA	887923	4755975	MG495889	MG495739
TE40Tor124BA	*Barbus plebejus*	124	Tordino	Tordino	PV	ITA	893143	4737787	MG495890	MG495740
TE41Tor124BA	*Barbus plebejus*	124	Tordino	Tordino	PV	ITA	893143	4737787	MG495891	MG495741
TE60Tor260BA	*Barbus plebejus*	260	Tordino	Tordino	PV	ITA	883688	4732232	MG495892	MG495742
106	*Barbus oscensis*	56	Picentino	Picentino	AC	ITA	996734	4519647	MG495893	MG495743
108	*Barbus oscensis*	56	Picentino	Picentino	AC	ITA	996734	4519647	MG495894	MG495744
112	*Barbus oscensis*	56	Picentino	Picentino	AC	ITA	996734	4519647	MG495895	MG495745
116	*Barbus oscensis*	56	Picentino	Picentino	AC	ITA	996734	4519647	MG495896	MG495746
122	*Barbus oscensis*	56	Picentino	Picentino	AC	ITA	996734	4519647	MG495897	MG495747
123	*Barbus oscensis*	56	Picentino	Picentino	AC	ITA	996734	4519647	MG495898	MG495748
124	*Barbus oscensis*	56	Picentino	Picentino	AC	ITA	996734	4519647	MG495899	MG495749
125	*Barbus oscensis*	56	Picentino	Picentino	AC	ITA	996734	4519647	MG495900	MG495750
PU136Bsc455BA	*Barbus plebejus*	455	Biscubio	Metauro	PV	ITA	777697	4829795	MG495901	MG495751

PU186Bss305BA	*Barbus plebejus*	305	Bosso	Metauro	PV	ITA	792225	4828182	MG495902	MG495752
PU187Bss305BA	*Barbus plebejus*	305	Bosso	Metauro	PV	ITA	792225	4828182	MG495903	MG495753
PU102Crt435BA	*Barbus plebejus*	435	Certano	Metauro	PV	ITA	785309	4822562	MG495904	MG495754
PU103Crt435BA	*Barbus plebejus*	435	Certano	Metauro	PV	ITA	785309	4822562	MG495905	MG495755
PU68Fgl85BA	*Barbus plebejus*	85	Foglia	Foglia	PV	ITA	793808	4855105	MG495906	MG495756
PU69Fgl85BA	*Barbus plebejus*	85	Foglia	Foglia	PV	ITA	793808	4855105	MG495907	MG495757
PU70Fgl85BA	*Barbus plebejus*	85	Foglia	Foglia	PV	ITA	793808	4855105	MG495908	MG495758
PU245Mta595BA	*Barbus plebejus*	595	Meta	Metauro	PV	ITA	762595	4834684	MG495909	MG495759
PU246Mta595BA	*Barbus plebejus*	595	Meta	Metauro	PV	ITA	762595	4834684	MG495910	MG495760
PU247Mta595BA	*Barbus plebejus*	595	Meta	Metauro	PV	ITA	762595	4834684	MG495911	MG495761
[Ω] Ofanto_01-M115	*Barbus oscensis*	340	Fiumara di Atella	Ofanto	AC	ITA	1052856	4546315	MG495912	MG495762
[ω] Ofanto_02-M113	*Barbus oscensis*	340	Fiumara di Atella	Ofanto	AC	ITA	1052856	4546315	MG495913	MG495763
[ω] Ofanto_03-M118	*Barbus oscensis*	340	Fiumara di Atella	Ofanto	AC	ITA	1052856	4546315	MG495914	MG495764
[ω] Ofanto_04-M104	*Barbus oscensis*	340	Fiumara di Atella	Ofanto	AC	ITA	1052856	4546315	MG495915	MG495765
Ofanto_05-M108	*Barbus oscensis*	340	Fiumara di Atella	Ofanto	AC	ITA	1052856	4546315	MG495916	MG495766
[ω] Ofanto_06-M119	*Barbus oscensis*	340	Fiumara di Atella	Ofanto	AC	ITA	1052856	4546315	MG495917	MG495767
[ω] Ofanto_07-M111	*Barbus oscensis*	340	Fiumara di Atella	Ofanto	AC	ITA	1052856	4546315	MG495918	MG495768
Ofanto_08-M106	*Barbus oscensis*	340	Fiumara di Atella	Ofanto	AC	ITA	1052856	4546315	MG495919	MG495769
Ofanto_09-M114	*Barbus oscensis*	340	Fiumara di Atella	Ofanto	AC	ITA	1052856	4546315	MG495920	MG495770
Ofanto_10-M116	*Barbus oscensis*	340	Fiumara di Atella	Ofanto	AC	ITA	1052856	4546315	MG495921	MG495771
Ofanto_11-M117	*Barbus oscensis*	340	Fiumara di Atella	Ofanto	AC	ITA	1052856	4546315	MG495922	MG495772
From [54]	*Barbus* cfr. *plebejus*				AC				MG718025	MK728797
	Barbus cfr. *plebejus*				AC				MK728816	MK728798
	Barbus cfr. *plebejus*				AC				MG718025	MK728799
	Barbus cfr. *plebejus*				AC				MG718026	MK728800
	Barbus cfr. *plebejus*				AC				MG718025	MK728801
	Barbus cfr. *plebejus*				AC				MK728817	MK728802

	Barbus cfr. *plebejus*	AC	MK728819	MK728808
	Barbus cfr. *plebejus*	AC	MK728821	MK728809
	Barbus cfr. *plebejus*	AC	MK728817	MK728810
	Barbus cfr. *plebejus*	AC	MK728817	MK728811
	Barbus cfr. *plebejus*	AC	MK728817	MK728812
	Barbus cfr. *plebejus*	AC	MK728817	MK728813
	Barbus cfr. *plebejus*	AC	MK728817	MK728814
	Barbus cfr. *plebejus*	AC	MK728817	MK728815
	Barbus cfr. *plebejus*	AC	MK728817	MK728803
	Barbus cfr. *plebejus*	AC	MK728820	MK728804
	Barbus cfr. *plebejus*	AC	MK728817	MK728805
	Barbus cfr. *plebejus*	AC	MK728819	MK728806
	Barbus cfr. *plebejus*	AC	MK728819	MK728807
Outgroups	*Luciobarbus graellsii*		JN049525	MG827110
	Cyprinus carpio		DQ868875	JN105352

[a] AC, Apulia-Campania district; PV, Padano-Venetian district; TL, Tuscany-Latium district; DAN, Danubian district.

[b] ITA, Italy; SLO, Slovenia.

[Ω] = holotype of *Barbus oscensis* sp. nov.

[ω] = paratypes of *Barbus oscensis* sp. nov.
