# Supplementary material for: Mitochondrial phylogeny and taxonomic revision of Italian and Slovenian fluvio-lacustrine barbels, Barbus sp. (Cypriniformes, Cyprinidae)
Source: BMC Zool. 2021 Apr 21;6:8. doi: 10.1186/s40850-021-00073-x (PMC10127354; doi:10.1186/s40850-021-00073-x)
Supplement: Supplementary file 5 — Additional file 5. Original phylogenetic trees in NEXUS format as computed by IQ-TREE, MrBayes, and RAxML, respectively. Each OTU is labeled with the resulting clade (see Fig. 2) and with GenBank Accession Numbers (Additional file 4). [file 40850_2021_73_MOESM5_ESM.doc]

**Additional file 5. Original phylogenetic trees in NEXUS format as computed by IQ-TREE, MrBayes, and RAxML, respectively.**

#NEXUS

begin taxa;

dimensions ntax=171;

taxlabels

Cyprinus_carpio_DQ868875_JN105352

Barbus_sp_TSAAC_clade_MG495773_MG495623

Barbus_tyberinus_TL_clade_MG495774_MG495624

Barbus_sp_TSAAC_clade_MG495775_MG495625

Barbus_plebejus_PV_clade_MG495776_MG495626

Barbus_plebejus_PV_clade_MG495777_MG495627

Barbus_plebejus_PV_clade_MG495778_MG495628

Barbus_plebejus_PV_clade_MG495779_MG495629

Barbus_plebejus_PV_clade_MG495780_MG495630

Barbus_plebejus_PV_clade_MG495781_MG495631

Barbus_plebejus_PV_clade_MG495782_MG495632

Barbus_barbus_MG495783_MG495633

Barbus_barbus_MG495784_MG495634

Barbus_tyberinus_TL_clade_MG495785_MG495635

Barbus_balcanicus_MG495786_MG495636

Barbus_balcanicus_MG495787_MG495637

Barbus_balcanicus_MG495788_MG495638

Barbus_balcanicus_MG495789_MG495639

Barbus_barbus_MG495790_MG495640

Barbus_barbus_MG495791_MG495641

Barbus_caninus_MG495792_MG495642

Barbus_caninus_MG495793_MG495643

Barbus_barbus_MG495794_MG495644

Barbus_barbus_MG495795_MG495645

Barbus_tyberinus_TL_clade_MG495796_MG495646

Barbus_sp_TSAAC_clade_MG495797_MG495647

Barbus_sp_TSAAC_clade_MG495798_MG495648

Barbus_sp_TSAAC_clade_MG495799_MG495649

Barbus_sp_TSAAC_clade_MG495800_MG495650

Barbus_sp_TSAAC_clade_MG495801_MG495651

Barbus_sp_TSAAC_clade_MG495802_MG495652

Barbus_sp_TSAAC_clade_MG495803_MG495653

Barbus_tyberinus_TL_clade_MG495804_MG495654

Barbus_plebejus_PV_clade_MG495805_MG495655

Barbus_barbus_MG495806_MG495656

Barbus_barbus_MG495807_MG495657

Barbus_tyberinus_TL_clade_MG495808_MG495658

Barbus_tyberinus_TL_clade_MG495809_MG495659

Barbus_tyberinus_TL_clade_MG495810_MG495660

Barbus_tyberinus_TL_clade_MG495811_MG495661

Barbus_tyberinus_TL_clade_MG495812_MG495662

Barbus_tyberinus_TL_clade_MG495813_MG495663

Barbus_tyberinus_TL_clade_MG495814_MG495664

Barbus_tyberinus_TL_clade_MG495815_MG495665

Barbus_tyberinus_TL_clade_MG495816_MG495666

Barbus_tyberinus_TL_clade_MG495817_MG495667

Barbus_plebejus_PV_clade_MG495818_MG495668

Barbus_tyberinus_TL_clade_MG495819_MG495669

Barbus_tyberinus_TL_clade_MG495820_MG495670

Barbus_barbus_MG495821_MG495671

Barbus_plebejus_PV_clade_MG495822_MG495672

Barbus_tyberinus_TL_clade_MG495823_MG495673

Barbus_tyberinus_TL_clade_MG495824_MG495674

Barbus_plebejus_PV_clade_MG495825_MG495675

Barbus_tyberinus_TL_clade_MG495826_MG495676

Barbus_plebejus_PV_clade_MG495827_MG495677

Barbus_tyberinus_TL_clade_MG495828_MG495678

Barbus_plebejus_PV_clade_MG495829_MG495679

Barbus_tyberinus_TL_clade_MG495830_MG495680

Barbus_barbus_MG495831_MG495681

Barbus_tyberinus_TL_clade_MG495832_MG495682

Barbus_barbus_MG495833_MG495683

Barbus_tyberinus_TL_clade_MG495834_MG495684

Barbus_tyberinus_TL_clade_MG495835_MG495685

Barbus_plebejus_PV_clade_MG495836_MG495686

Barbus_tyberinus_TL_clade_MG495837_MG495687

Luciobarbus_graellsii_MG495838_MG495688

Luciobarbus_graellsii_MG495839_MG495689

Barbus_tyberinus_TL_clade_MG495840_MG495690

Barbus_tyberinus_TL_clade_MG495841_MG495691

Barbus_plebejus_PV_clade_MG495842_MG495692

Luciobarbus_graellsii_MG495843_MG495693

Barbus_barbus_MG495844_MG495694

Barbus_tyberinus_TL_clade_MG495845_MG495695

Barbus_tyberinus_TL_clade_MG495846_MG495696

Barbus_barbus_MG495847_MG495697

Barbus_plebejus_PV_clade_MG495848_MG495698

Barbus_tyberinus_TL_clade_MG495849_MG495699

Barbus_tyberinus_TL_clade_MG495850_MG495700

Barbus_tyberinus_TL_clade_MG495851_MG495701

Barbus_tyberinus_TL_clade_MG495852_MG495702

Barbus_barbus_MG495853_MG495703

Barbus_barbus_MG495854_MG495704

Barbus_barbus_MG495855_MG495705

Barbus_plebejus_PV_clade_MG495856_MG495706

Barbus_plebejus_PV_clade_MG495857_MG495707

Barbus_plebejus_PV_clade_MG495858_MG495708

Barbus_plebejus_PV_clade_MG495859_MG495709

Barbus_plebejus_PV_clade_MG495860_MG495710

Barbus_plebejus_PV_clade_MG495861_MG495711

Barbus_plebejus_PV_clade_MG495862_MG495712

Barbus_plebejus_PV_clade_MG495863_MG495713

Barbus_plebejus_PV_clade_MG495864_MG495714

Barbus_balcanicus_MG495865_MG495715

Barbus_tyberinus_TL_clade_MG495866_MG495716

Barbus_caninus_MG495867_MG495717

Barbus_caninus_MG495868_MG495718

Barbus_plebejus_PV_clade_MG495869_MG495719

Barbus_barbus_MG495870_MG495720

Barbus_plebejus_PV_clade_MG495871_MG495721

Barbus_plebejus_PV_clade_MG495872_MG495722

Barbus_plebejus_PV_clade_MG495873_MG495723

Barbus_sp_TSAAC_clade_MG495874_MG495724

Barbus_sp_TSAAC_clade_MG495875_MG495725

Barbus_sp_TSAAC_clade_MG495876_MG495727

Barbus_sp_TSAAC_clade_MG495877_MG495726

Barbus_sp_TSAAC_clade_MG495878_MG495728

Barbus_barbus_MG495879_MG495729

Barbus_barbus_MG495880_MG495730

Barbus_plebejus_PV_clade_MG495881_MG495731

Barbus_plebejus_PV_clade_MG495882_MG495732

Barbus_plebejus_PV_clade_MG495883_MG495733

Barbus_plebejus_PV_clade_MG495884_MG495734

Barbus_balcanicus_MG495885_MG495735

Barbus_barbus_MG495886_MG495736

Barbus_balcanicus_MG495887_MG495737

Barbus_barbus_MG495888_MG495738

Barbus_plebejus_PV_clade_MG495889_MG495739

Barbus_plebejus_PV_clade_MG495890_MG495740

Barbus_plebejus_PV_clade_MG495891_MG495741

Barbus_plebejus_PV_clade_MG495892_MG495742

Barbus_sp_TSAAC_clade_MG495893_MG495743

Barbus_sp_TSAAC_clade_MG495894_MG495744

Barbus_sp_TSAAC_clade_MG495895_MG495745

Barbus_sp_TSAAC_clade_MG495896_MG495746

Barbus_sp_TSAAC_clade_MG495897_MG495747

Barbus_sp_TSAAC_clade_MG495898_MG495748

Barbus_sp_TSAAC_clade_MG495899_MG495749

Barbus_sp_TSAAC_clade_MG495900_MG495750

Barbus_plebejus_PV_clade_MG495901_MG495751

Barbus_plebejus_PV_clade_MG495902_MG495752

Barbus_plebejus_PV_clade_MG495903_MG495753

Barbus_plebejus_PV_clade_MG495904_MG495754

Barbus_plebejus_PV_clade_MG495905_MG495755

Barbus_plebejus_PV_clade_MG495906_MG495756

Barbus_plebejus_PV_clade_MG495907_MG495757

Barbus_plebejus_PV_clade_MG495908_MG495758

Barbus_plebejus_PV_clade_MG495909_MG495759

Barbus_plebejus_PV_clade_MG495910_MG495760

Barbus_plebejus_PV_clade_MG495911_MG495761

Barbus_sp_TSAAC_clade_MG495912_MG495762

Barbus_sp_TSAAC_clade_MG495913_MG495763

Barbus_sp_TSAAC_clade_MG495914_MG495764

Barbus_sp_TSAAC_clade_MG495915_MG495765

Barbus_sp_TSAAC_clade_MG495916_MG495766

Barbus_sp_TSAAC_clade_MG495917_MG495767

Barbus_sp_TSAAC_clade_MG495918_MG495768

Barbus_sp_TSAAC_clade_MG495919_MG495769

Barbus_sp_TSAAC_clade_MG495920_MG495770

Barbus_sp_TSAAC_clade_MG495921_MG495771

Barbus_sp_TSAAC_clade_MG495922_MG495772

Barbus_sp_TSAAC_clade_MG495912_MG495762

Barbus_sp_TSAAC_clade_MG495913_MG495763

Barbus_sp_TSAAC_clade_MG495914_MG495764

Barbus_sp_TSAAC_clade_MG495915_MG495765

Barbus_sp_TSAAC_clade_MG495916_MG495766

Barbus_sp_TSAAC_clade_MG495917_MG495767

Barbus_sp_TSAAC_clade_MG495918_MG495768

Barbus_sp_TSAAC_clade_MG495919_MG495769

Barbus_sp_TSAAC_clade_MG495920_MG495770

Barbus_sp_TSAAC_clade_MG495921_MG495771

Barbus_sp_TSAAC_clade_MG495922_MG495772

Barbus_sp_clade_4_MG718025_MK728797

Barbus_sp_clade_4_MK728816_MK728798

Barbus_sp_clade_4_MG718025_MK728799

Barbus_sp_clade_4_MG718026_MK728800

Barbus_sp_clade_4_MG718025_MK728801

Barbus_sp_clade_4_MK728817_MK728802

Barbus_sp_TSAAC_clade_MK728819_MK728808

Barbus_sp_TSAAC_clade_MK728821_MK728809

Barbus_sp_clade_4_MK728817_MK728810

Barbus_sp_clade_4_MK728817_MK728811

Barbus_sp_clade_4_MK728817_MK728812

Barbus_sp_clade_4_MK728817_MK728813

Barbus_sp_clade_4_MK728817_MK728814

Barbus_sp_clade_4_MK728817_MK728815

Barbus_sp_clade_4_MK728817_MK728803

Barbus_sp_TSAAC_clade_MK728820_MK728804

Barbus_sp_clade_4_MK728817_MK728805

Barbus_sp_TSAAC_clade_MK728819_MK728806

Barbus_sp_TSAAC_clade_MK728819_MK728807

Luciobarbus_graellsii_JN049525_MG827110

;

tree IQ-TREE_tree = (Cyprinus_carpio_DQ868875_JN105352:0.20267,(Luciobarbus_graellsii_JN049525_MG827110:0.0243417,(Luciobarbus_graellsii_MG495843_MG495693:2.5445e-06,((Luciobarbus_graellsii_MG495838_MG495688:0.00114008,Luciobarbus_graellsii_MG495839_MG495689:2.1128e-06)100:0.18589,(((((Barbus_balcanicus_MG495786_MG495636:0.000562079,(Barbus_balcanicus_MG495787_MG495637:0.000840641,Barbus_balcanicus_MG495865_MG495715:0.00284268)84:0.00115857)81:0.000187515,(Barbus_balcanicus_MG495788_MG495638:0.00498259,Barbus_balcanicus_MG495885_MG495735:0.00445592)83:0.00298872)100:0.0390291,(Barbus_balcanicus_MG495789_MG495639:2.1128e-06,Barbus_balcanicus_MG495887_MG495737:0.000792264)100:0.00758305)100:0.0314603,(((Barbus_caninus_MG495792_MG495642:0.00087137,Barbus_caninus_MG495868_MG495718:0.00131783)98:0.00602243,Barbus_caninus_MG495793_MG495643:0.0131144)100:0.0236446,Barbus_caninus_MG495867_MG495717:0.00194441)100:0.025526)93:0.0161906,(((((((Barbus_barbus_MG495783_MG495633:0.00117621,(Barbus_barbus_MG495807_MG495657:2.1128e-06,Barbus_barbus_MG495821_MG495671:0.00273977)74:0.000687199)91:0.0011396,(Barbus_barbus_MG495795_MG495645:0.00113729,Barbus_barbus_MG495831_MG495681:0.00113897)76:0.000575522)92:0.00171031,Barbus_barbus_MG495888_MG495738:0.00170696)25:2.1128e-06,(Barbus_barbus_MG495833_MG495683:0.00940811,Barbus_barbus_MG495844_MG495694:0.00056733)16:2.1128e-06)41:2.1128e-06,(((((Barbus_barbus_MG495790_MG495640:0.000804415,((Barbus_barbus_MG495847_MG495697:0.00113871,Barbus_barbus_MG495870_MG495720:0.000563416)76:0.000558137,Barbus_barbus_MG495854_MG495704:0.00496655)19:2.1128e-06)8:2.1128e-06,Barbus_barbus_MG495853_MG495703:2.1128e-06)2:2.1128e-06,((Barbus_barbus_MG495791_MG495641:0.00492269,Barbus_barbus_MG495855_MG495705:2.1128e-06)45:0.00080343,(Barbus_barbus_MG495794_MG495644:0.00161059,Barbus_barbus_MG495886_MG495736:0.000802092)6:2.1128e-06)2:2.1128e-06)3:2.1128e-06,Barbus_barbus_MG495879_MG495729:0.000805142)10:2.1128e-06,Barbus_barbus_MG495806_MG495656:0.000700263)34:2.1128e-06)52:0.000756582,(Barbus_barbus_MG495784_MG495634:0.00161219,Barbus_barbus_MG495880_MG495730:0.000800361)42:2.1128e-06)100:0.0262881,(((((Barbus_tyberinus_TL_clade_MG495774_MG495624:0.00249647,Barbus_tyberinus_TL_clade_MG495796_MG495646:0.00114469)75:2.3917e-06,((((((((Barbus_tyberinus_TL_clade_MG495785_MG495635:0.00114071,Barbus_tyberinus_TL_clade_MG495808_MG495658:0.00228913)24:2.1128e-06,Barbus_tyberinus_TL_clade_MG495852_MG495702:0.00057019)14:2.1128e-06,(Barbus_tyberinus_TL_clade_MG495804_MG495654:2.1128e-06,Barbus_tyberinus_TL_clade_MG495846_MG495696:2.1128e-06)14:2.1128e-06)3:2.1128e-06,(Barbus_tyberinus_TL_clade_MG495828_MG495678:0.000569974,((Barbus_tyberinus_TL_clade_MG495830_MG495680:2.1128e-06,Barbus_tyberinus_TL_clade_MG495851_MG495701:2.1128e-06)92:0.000568405,Barbus_tyberinus_TL_clade_MG495850_MG495700:0.00113807)14:2.1128e-06)5:2.1128e-06)5:2.1128e-06,((((Barbus_tyberinus_TL_clade_MG495809_MG495659:2.1128e-06,Barbus_tyberinus_TL_clade_MG495810_MG495660:0.00113883)100:0.00461006,(Barbus_tyberinus_TL_clade_MG495811_MG495661:0.00114736,Barbus_tyberinus_TL_clade_MG495820_MG495670:0.000573205)70:0.000567734)19:2.1128e-06,((Barbus_tyberinus_TL_clade_MG495812_MG495662:0.00114001,Barbus_tyberinus_TL_clade_MG495816_MG495666:0.000568527)35:2.1128e-06,((((Barbus_tyberinus_TL_clade_MG495814_MG495664:0,Barbus_tyberinus_TL_clade_MG495819_MG495669:0):0,Barbus_tyberinus_TL_clade_MG495824_MG495674:0):0,Barbus_tyberinus_TL_clade_MG495817_MG495667:0):2.1128e-06,Barbus_tyberinus_TL_clade_MG495815_MG495665:2.1128e-06)30:2.1128e-06)27:2.1128e-06)21:2.1128e-06,Barbus_tyberinus_TL_clade_MG495845_MG495695:0.000570238)9:2.1128e-06)4:2.1128e-06,(Barbus_tyberinus_TL_clade_MG495826_MG495676:0.000569703,Barbus_tyberinus_TL_clade_MG495849_MG495699:0.000569636)9:2.1128e-06)21:2.1128e-06,Barbus_tyberinus_TL_clade_MG495813_MG495663:0.00286911)78:0.00114244,((Barbus_tyberinus_TL_clade_MG495823_MG495673:2.1128e-06,Barbus_tyberinus_TL_clade_MG495837_MG495687:0.000573724)61:0.000574124,((Barbus_tyberinus_TL_clade_MG495832_MG495682:2.1128e-06,Barbus_tyberinus_TL_clade_MG495840_MG495690:2.1128e-06)92:2.1128e-06,Barbus_tyberinus_TL_clade_MG495841_MG495691:0.000574678)51:2.1128e-06)51:0.000572887)25:2.1128e-06)36:0.000570846,(Barbus_tyberinus_TL_clade_MG495834_MG495684:2.1128e-06,Barbus_tyberinus_TL_clade_MG495835_MG495685:0.000568762)60:2.1128e-06)57:0.00127929,Barbus_tyberinus_TL_clade_MG495866_MG495716:2.1128e-06)100:0.00802839,((((((Barbus_sp_clade_4_MG718025_MK728797:2.1128e-06,Barbus_sp_clade_4_MG718025_MK728799:2.1128e-06)67:2.1128e-06,Barbus_sp_clade_4_MG718025_MK728801:0.000759554)38:2.1128e-06,Barbus_sp_clade_4_MG718026_MK728800:0.000759336)68:0.000725622,(((((((Barbus_sp_clade_4_MK728817_MK728802:0,Barbus_sp_clade_4_MK728817_MK728813:0):0,Barbus_sp_clade_4_MK728817_MK728803:0):2.1128e-06,Barbus_sp_clade_4_MK728817_MK728811:2.1128e-06)91:2.1128e-06,Barbus_sp_clade_4_MK728817_MK728815:0.000582114)19:2.1128e-06,(Barbus_sp_clade_4_MK728817_MK728812:2.1128e-06,Barbus_sp_clade_4_MK728817_MK728814:2.1128e-06)94:0.000581468)31:2.1128e-06,Barbus_sp_clade_4_MK728817_MK728805:0.000581049)88:0.00117152,Barbus_sp_clade_4_MK728817_MK728810:2.1128e-06)100:0.00584315)90:0.00119916,Barbus_sp_clade_4_MK728816_MK728798:0.00523594)98:0.00525139,((((((((((((Barbus_plebejus_PV_clade_MG495776_MG495626:2.1128e-06,(Barbus_plebejus_PV_clade_MG495777_MG495627:0.000819104,Barbus_plebejus_PV_clade_MG495891_MG495741:0.000818358)36:2.1128e-06)43:2.1128e-06,(Barbus_plebejus_PV_clade_MG495889_MG495739:0.000575992,((Barbus_plebejus_PV_clade_MG495892_MG495742:0.000575696,Barbus_plebejus_PV_clade_MG495905_MG495755:0.00176243)16:2.1128e-06,Barbus_plebejus_PV_clade_MG495902_MG495752:0.000576456)22:2.1128e-06)56:0.000575905)23:2.1128e-06,(((Barbus_plebejus_PV_clade_MG495805_MG495655:2.1128e-06,Barbus_plebejus_PV_clade_MG495842_MG495692:2.1128e-06)95:2.1128e-06,(((Barbus_plebejus_PV_clade_MG495858_MG495708:0.00142283,Barbus_plebejus_PV_clade_MG495906_MG495756:2.1128e-06)40:2.1128e-06,Barbus_plebejus_PV_clade_MG495873_MG495723:0.00331948)58:0.00115311,(Barbus_plebejus_PV_clade_MG495861_MG495711:0.000821789,Barbus_plebejus_PV_clade_MG495881_MG495731:0.000822301)29:2.1128e-06)40:2.1128e-06)34:2.1128e-06,Barbus_plebejus_PV_clade_MG495903_MG495753:0.00232908)35:0.000576619)38:0.000576622,Barbus_plebejus_PV_clade_MG495836_MG495686:0.00057747)8:2.1128e-06,(((Barbus_plebejus_PV_clade_MG495778_MG495628:0.00154678,(Barbus_plebejus_PV_clade_MG495779_MG495629:2.1128e-06,(Barbus_plebejus_PV_clade_MG495856_MG495706:2.1128e-06,Barbus_plebejus_PV_clade_MG495859_MG495709:0.000709617)39:2.1128e-06)33:2.1128e-06)64:0.00076859,Barbus_plebejus_PV_clade_MG495860_MG495710:2.1128e-06)32:2.1128e-06,(((Barbus_plebejus_PV_clade_MG495818_MG495668:0.000577698,(Barbus_plebejus_PV_clade_MG495904_MG495754:0.00057773,Barbus_plebejus_PV_clade_MG495907_MG495757:0.000577491)28:2.1128e-06)19:2.1128e-06,Barbus_plebejus_PV_clade_MG495822_MG495672:2.1128e-06)19:2.1128e-06,(((Barbus_plebejus_PV_clade_MG495869_MG495719:0.0127949,(Barbus_plebejus_PV_clade_MG495909_MG495759:0.00278247,Barbus_plebejus_PV_clade_MG495910_MG495760:0.00432943)62:0.0017386)100:0.00339781,Barbus_plebejus_PV_clade_MG495871_MG495721:0.000577005)86:0.0011663,Barbus_plebejus_PV_clade_MG495872_MG495722:2.1128e-06)54:0.000577691)26:2.1128e-06)3:2.1128e-06)7:2.1128e-06,(((((((Barbus_plebejus_PV_clade_MG495780_MG495630:0.000825798,Barbus_plebejus_PV_clade_MG495882_MG495732:2.1128e-06)43:2.1128e-06,Barbus_plebejus_PV_clade_MG495864_MG495714:0.00165065)64:0.000823167,Barbus_plebejus_PV_clade_MG495827_MG495677:0.0011533)19:2.1128e-06,(Barbus_plebejus_PV_clade_MG495883_MG495733:2.1128e-06,(Barbus_plebejus_PV_clade_MG495884_MG495734:0.00417408,Barbus_plebejus_PV_clade_MG495890_MG495740:0.00668882)45:2.1128e-06)61:0.000823326)14:2.1128e-06,Barbus_plebejus_PV_clade_MG495863_MG495713:0.00165032)25:2.1128e-06,(Barbus_plebejus_PV_clade_MG495825_MG495675:0.00115976,Barbus_plebejus_PV_clade_MG495911_MG495761:0.00174076)16:2.1128e-06)8:2.1128e-06,Barbus_plebejus_PV_clade_MG495862_MG495712:0.000578024)26:0.000577593)28:2.1128e-06,Barbus_plebejus_PV_clade_MG495782_MG495632:0.000819381)27:0.000576009,(Barbus_plebejus_PV_clade_MG495848_MG495698:0.000580054,Barbus_plebejus_PV_clade_MG495857_MG495707:2.1128e-06)19:2.1128e-06)19:0.000579368,(Barbus_plebejus_PV_clade_MG495901_MG495751:0.00116273,Barbus_plebejus_PV_clade_MG495908_MG495758:0.00174773)19:2.0302e-06)26:0.000579897,Barbus_plebejus_PV_clade_MG495829_MG495679:2.0712e-06)62:0.00233465,Barbus_plebejus_PV_clade_MG495781_MG495631:2.1128e-06)88:0.00172054,(Barbus_sp_TSAAC_clade_MG495893_MG495743:2.1128e-06,(Barbus_sp_TSAAC_clade_MG495898_MG495748:0.0011473,(Barbus_sp_TSAAC_clade_MG495899_MG495749:0.00114953,((Barbus_sp_TSAAC_clade_MG495878_MG495728:0.00116313,((((Barbus_sp_TSAAC_clade_MG495894_MG495744:0,Barbus_sp_TSAAC_clade_MG495896_MG495746:0):0,Barbus_sp_TSAAC_clade_MG495900_MG495750:0):2.1128e-06,Barbus_sp_TSAAC_clade_MG495895_MG495745:2.1128e-06)74:2.1128e-06,(Barbus_sp_TSAAC_clade_MG495897_MG495747:0.000579238,(Barbus_sp_TSAAC_clade_MG495917_MG495767:2.1128e-06,Barbus_sp_TSAAC_clade_MG495920_MG495770:2.1128e-06)93:2.1128e-06)35:2.1128e-06)62:2.1128e-06)87:0.000580744,(((((Barbus_sp_TSAAC_clade_MG495798_MG495648:2.1128e-06,(Barbus_sp_TSAAC_clade_MG495799_MG495649:0.00289536,Barbus_sp_TSAAC_clade_MG495875_MG495725:0.00115013)56:2.1128e-06)26:2.1128e-06,Barbus_sp_TSAAC_clade_MG495800_MG495650:2.1128e-06)41:2.1128e-06,Barbus_sp_TSAAC_clade_MG495803_MG495653:2.1128e-06)45:2.1128e-06,Barbus_sp_TSAAC_clade_MG495802_MG495652:2.1128e-06)75:2.1128e-06,((((Barbus_sp_TSAAC_clade_MG495874_MG495724:0.0011762,Barbus_sp_TSAAC_clade_MG495876_MG495727:0.00355028)88:0.00115163,Barbus_sp_TSAAC_clade_MG495877_MG495726:0.00116027)22:2.1128e-06,(((((((((Barbus_sp_TSAAC_clade_MG495912_MG495762:0,Barbus_sp_TSAAC_clade_MG495922_MG495772:0):0,Ofanto_08-MBarbus_sp_TSAAC_clade_MG495893_MG495743:0):0,Ofanto_05-MBarbus_sp_TSAAC_clade_MG495894_MG495744:0):0,Barbus_sp_TSAAC_clade_MG495915_MG495765:0):0,Barbus_sp_TSAAC_clade_MG495918_MG495768:0):0,Ofanto_10-MBarbus_sp_TSAAC_clade_MG495896_MG495746:0):2.1128e-06,Barbus_sp_TSAAC_clade_MG495914_MG495764:2.1128e-06)95:2.1128e-06,Barbus_sp_TSAAC_clade_MK728820_MK728804:0.000583927)59:2.1128e-06,Barbus_sp_TSAAC_clade_MG495913_MG495763:2.1128e-06)62:2.1128e-06)16:2.1128e-06,(((Barbus_sp_TSAAC_clade_MG495797_MG495647:2.1128e-06,(Barbus_sp_TSAAC_clade_MK728819_MK728808:2.1128e-06,Barbus_sp_TSAAC_clade_MK728819_MK728806:2.1128e-06)93:2.1128e-06)63:2.1128e-06,(Barbus_sp_TSAAC_clade_MG495801_MG495651:2.1128e-06,Barbus_sp_TSAAC_clade_MK728819_MK728807:2.1128e-06)64:0.000576943)95:0.00232448,(Barbus_sp_TSAAC_clade_MK728821_MK728809:2.1128e-06,(Barbus_sp_TSAAC_clade_MG495773_MG495623:0.000818475,Barbus_sp_TSAAC_clade_MG495775_MG495625:2.1128e-06)64:0.000577316)88:0.00116479)19:2.1128e-06)8:2.1128e-06)41:0.000580452)95:0.00772489)40:2.1128e-06)53:0.00057509)97:0.00718879)55:0.00226486)61:0.00325017)95:0.0082343)99:0.01746)97:0.0294301)83:0.0370405)83:0.0338364):0.0106668);

tree MrBayes_tree = (Cyprinus_carpio_DQ868875_JN105352:0.103858,((Luciobarbus_graellsii_MG495838_MG495688:0.00119007,Luciobarbus_graellsii_MG495839_MG495689:0.000380831)1.000:0.0933278,(((((((((((((((((((((((((((((Barbus_sp_TSAAC_clade_MG495773_MG495623:0.00111326,Barbus_sp_TSAAC_clade_MG495775_MG495625:0.000331694)0.985:0.000813323,Barbus_sp_TSAAC_clade_MK728821_MK728809:0.00035619)0.993:0.00123299,(((Barbus_sp_TSAAC_clade_MG495797_MG495647:0.000392188,(Barbus_sp_TSAAC_clade_MG495801_MG495651:0.000400172,Barbus_sp_TSAAC_clade_MK728819_MK728807:0.000338287)0.976:0.000795131):0,Barbus_sp_TSAAC_clade_MK728819_MK728808:0.000349508):0,Barbus_sp_TSAAC_clade_MK728819_MK728806:0.00032735)1.000:0.00216925):0,Barbus_sp_TSAAC_clade_MG495798_MG495648:0.000402166):0,Barbus_sp_TSAAC_clade_MG495799_MG495649:0.00271559):0,Barbus_sp_TSAAC_clade_MG495800_MG495650:0.000398958):0,Barbus_sp_TSAAC_clade_MG495802_MG495652:0.00039896):0,Barbus_sp_TSAAC_clade_MG495803_MG495653:0.000402003):0,(Barbus_sp_TSAAC_clade_MG495874_MG495724:0.0014121,Barbus_sp_TSAAC_clade_MG495876_MG495727:0.00336342)0.956:0.0012709):0,Barbus_sp_TSAAC_clade_MG495875_MG495725:0.00110882):0,Barbus_sp_TSAAC_clade_MG495877_MG495726:0.00130875):0,(((((((Barbus_sp_TSAAC_clade_MG495878_MG495728:0.00130455,Barbus_sp_TSAAC_clade_MG495894_MG495744:0.000329059):0,Barbus_sp_TSAAC_clade_MG495895_MG495745:0.000334411):0,Barbus_sp_TSAAC_clade_MG495896_MG495746:0.000349134):0,Barbus_sp_TSAAC_clade_MG495897_MG495747:0.000806773):0,Barbus_sp_TSAAC_clade_MG495900_MG495750:0.000341192):0,Barbus_sp_TSAAC_clade_MG495917_MG495767:0.000349186):0,Barbus_sp_TSAAC_clade_MG495920_MG495770:0.00035647)0.964:0.00123435):0,Barbus_sp_TSAAC_clade_MG495912_MG495762:0.000336745):0,Barbus_sp_TSAAC_clade_MG495913_MG495763:0.000329381):0,Barbus_sp_TSAAC_clade_MG495914_MG495764:0.000339863):0,Barbus_sp_TSAAC_clade_MG495915_MG495765:0.000342517):0,MBarbus_sp_TSAAC_clade_MG495894_MG495744:0.000354665):0,Barbus_sp_TSAAC_clade_MG495918_MG495768:0.000344186):0,MBarbus_sp_TSAAC_clade_MG495893_MG495743:0.000331893):0,MBarbus_sp_TSAAC_clade_MG495896_MG495746:0.000333659):0,Barbus_sp_TSAAC_clade_MG495922_MG495772:0.000331675):0,Barbus_sp_TSAAC_clade_MK728820_MK728804:0.000814252)1.000:0.00584568,((Barbus_sp_TSAAC_clade_MG495893_MG495743:0.000576298,Barbus_sp_TSAAC_clade_MG495898_MG495748:0.0012934):0,Barbus_sp_TSAAC_clade_MG495899_MG495749:0.00130439)0.730:0.00210879)1.000:0.00583371,(((((((((((((((((((((((((((((Barbus_plebejus_PV_clade_MG495776_MG495626:0.000448957,Barbus_plebejus_PV_clade_MG495777_MG495627:0.00115057):0,(((Barbus_plebejus_PV_clade_MG495778_MG495628:0.00155002,Barbus_plebejus_PV_clade_MG495779_MG495629:0.000408141):0,Barbus_plebejus_PV_clade_MG495856_MG495706:0.00040148):0,Barbus_plebejus_PV_clade_MG495859_MG495709:0.00100318)0.683:0.000961174):0,Barbus_plebejus_PV_clade_MG495782_MG495632:0.00111225):0,((((Barbus_plebejus_PV_clade_MG495805_MG495655:0.000342608,Barbus_plebejus_PV_clade_MG495842_MG495692:0.000347134):0,Barbus_plebejus_PV_clade_MG495861_MG495711:0.00110436):0,Barbus_plebejus_PV_clade_MG495881_MG495731:0.00113857):0,Barbus_plebejus_PV_clade_MG495903_MG495753:0.00225713)0.659:0.00080017):0,Barbus_plebejus_PV_clade_MG495818_MG495668:0.000808852):0,Barbus_plebejus_PV_clade_MG495822_MG495672:0.00034495):0,Barbus_plebejus_PV_clade_MG495829_MG495679:0.000738446):0,Barbus_plebejus_PV_clade_MG495836_MG495686:0.000816248):0,Barbus_plebejus_PV_clade_MG495848_MG495698:0.000834574):0,Barbus_plebejus_PV_clade_MG495857_MG495707:0.00043341):0,Barbus_plebejus_PV_clade_MG495858_MG495708:0.00154354):0,Barbus_plebejus_PV_clade_MG495860_MG495710:0.00041031):0,(((Barbus_plebejus_PV_clade_MG495869_MG495719:0.0107479,(Barbus_plebejus_PV_clade_MG495909_MG495759:0.00246932,Barbus_plebejus_PV_clade_MG495910_MG495760:0.00405781)0.944:0.00175554)1.000:0.00337135,Barbus_plebejus_PV_clade_MG495871_MG495721:0.000748768)1.000:0.00138093,Barbus_plebejus_PV_clade_MG495872_MG495722:0.00041979)0.703:0.000774945):0,Barbus_plebejus_PV_clade_MG495873_MG495723:0.00305748):0,(((Barbus_plebejus_PV_clade_MG495889_MG495739:0.000818465,Barbus_plebejus_PV_clade_MG495892_MG495742:0.000780018):0,Barbus_plebejus_PV_clade_MG495902_MG495752:0.000788267):0,Barbus_plebejus_PV_clade_MG495905_MG495755:0.00178743)0.754:0.000813907):0,Barbus_plebejus_PV_clade_MG495891_MG495741:0.000981194):0,Barbus_plebejus_PV_clade_MG495901_MG495751:0.00127093):0,Barbus_plebejus_PV_clade_MG495904_MG495754:0.000811386):0,Barbus_plebejus_PV_clade_MG495906_MG495756:0.000622673):0,Barbus_plebejus_PV_clade_MG495907_MG495757:0.000795011):0,Barbus_plebejus_PV_clade_MG495908_MG495758:0.00171064)0.657:0.000821925,((Barbus_plebejus_PV_clade_MG495780_MG495630:0.00110748,Barbus_plebejus_PV_clade_MG495864_MG495714:0.00179229):0,Barbus_plebejus_PV_clade_MG495882_MG495732:0.000469463)0.748:0.00102706):0,Barbus_plebejus_PV_clade_MG495825_MG495675:0.00124511):0,Barbus_plebejus_PV_clade_MG495827_MG495677:0.00120346):0,Barbus_plebejus_PV_clade_MG495862_MG495712:0.000825409):0,Barbus_plebejus_PV_clade_MG495863_MG495713:0.00180672):0,((Barbus_plebejus_PV_clade_MG495883_MG495733:0.00044593,Barbus_plebejus_PV_clade_MG495884_MG495734:0.0037123):0,Barbus_plebejus_PV_clade_MG495890_MG495740:0.00569113)0.868:0.00111538):0,Barbus_plebejus_PV_clade_MG495911_MG495761:0.00183257)0.803:0.00133559,Barbus_plebejus_PV_clade_MG495781_MG495631:0.000561647)1.000:0.00292155)0.615:0.00204158,(((((Barbus_sp_clade_4_MG718025_MK728797:0.00041834,Barbus_sp_clade_4_MG718025_MK728799:0.000412812):0,Barbus_sp_clade_4_MG718026_MK728800:0.00101237):0,Barbus_sp_clade_4_MG718025_MK728801:0.00103397)0.825:0.00102628,(((((((Barbus_sp_clade_4_MK728817_MK728802:0.000338591,Barbus_sp_clade_4_MK728817_MK728811:0.000339903):0,(Barbus_sp_clade_4_MK728817_MK728812:0.00034797,Barbus_sp_clade_4_MK728817_MK728814:0.000349779)0.983:0.000820818):0,Barbus_sp_clade_4_MK728817_MK728813:0.000340902):0,Barbus_sp_clade_4_MK728817_MK728815:0.000826634):0,Barbus_sp_clade_4_MK728817_MK728803:0.00033767):0,Barbus_sp_clade_4_MK728817_MK728805:0.000801894)0.999:0.00129759,Barbus_sp_clade_4_MK728817_MK728810:0.000346825)1.000:0.00497276)0.960:0.00171561,Barbus_sp_clade_4_MK728816_MK728798:0.00459904)1.000:0.00481467)0.954:0.00305807,((((((Barbus_tyberinus_TL_clade_MG495774_MG495624:0.00244587,((((((((((((((((((((Barbus_tyberinus_TL_clade_MG495785_MG495635:0.0012869,Barbus_tyberinus_TL_clade_MG495804_MG495654:0.000330856):0,Barbus_tyberinus_TL_clade_MG495808_MG495658:0.00229517):0,(Barbus_tyberinus_TL_clade_MG495809_MG495659:0.000338392,Barbus_tyberinus_TL_clade_MG495810_MG495660:0.00129927)0.998:0.00423739):0,(Barbus_tyberinus_TL_clade_MG495811_MG495661:0.00145443,Barbus_tyberinus_TL_clade_MG495820_MG495670:0.000945226)0.690:0.000802745):0,Barbus_tyberinus_TL_clade_MG495812_MG495662:0.00128166):0,Barbus_tyberinus_TL_clade_MG495813_MG495663:0.00274364):0,Barbus_tyberinus_TL_clade_MG495814_MG495664:0.000329944):0,Barbus_tyberinus_TL_clade_MG495815_MG495665:0.000328011):0,Barbus_tyberinus_TL_clade_MG495816_MG495666:0.000794241):0,Barbus_tyberinus_TL_clade_MG495817_MG495667:0.000328587):0,Barbus_tyberinus_TL_clade_MG495819_MG495669:0.000332271):0,Barbus_tyberinus_TL_clade_MG495824_MG495674:0.000334261):0,Barbus_tyberinus_TL_clade_MG495826_MG495676:0.000815465):0,Barbus_tyberinus_TL_clade_MG495828_MG495678:0.000809349):0,(Barbus_tyberinus_TL_clade_MG495830_MG495680:0.000337436,Barbus_tyberinus_TL_clade_MG495851_MG495701:0.000355166)0.963:0.000806179):0,Barbus_tyberinus_TL_clade_MG495845_MG495695:0.000805194):0,Barbus_tyberinus_TL_clade_MG495846_MG495696:0.000329459):0,Barbus_tyberinus_TL_clade_MG495849_MG495699:0.000856592):0,Barbus_tyberinus_TL_clade_MG495850_MG495700:0.00130887):0,Barbus_tyberinus_TL_clade_MG495852_MG495702:0.000803238)0.855:0.00117015):0,Barbus_tyberinus_TL_clade_MG495796_MG495646:0.00106742):0,((((Barbus_tyberinus_TL_clade_MG495823_MG495673:0.000340862,Barbus_tyberinus_TL_clade_MG495837_MG495687:0.000814243)0.718:0.000739202,Barbus_tyberinus_TL_clade_MG495832_MG495682:0.000349779):0,Barbus_tyberinus_TL_clade_MG495840_MG495690:0.00033476):0,Barbus_tyberinus_TL_clade_MG495841_MG495691:0.000806951)0.521:0.000822597)0.644:0.000835897,Barbus_tyberinus_TL_clade_MG495834_MG495684:0.000333568):0,Barbus_tyberinus_TL_clade_MG495835_MG495685:0.000802716)0.685:0.00145311,Barbus_tyberinus_TL_clade_MG495866_MG495716:0.000691553)1.000:0.00726906)1.000:0.00687935,((((((((((((((((Barbus_barbus_MG495783_MG495633:0.00133611,(Barbus_barbus_MG495807_MG495657:0.0004152,Barbus_barbus_MG495821_MG495671:0.00262675)0.951:0.000929245)0.995:0.00132425,(Barbus_barbus_MG495795_MG495645:0.00131162,Barbus_barbus_MG495831_MG495681:0.00128278)0.974:0.00082088)0.986:0.00169099,(Barbus_barbus_MG495784_MG495634:0.00179653,Barbus_barbus_MG495880_MG495730:0.00120199)0.533:0.000981007):0,Barbus_barbus_MG495790_MG495640:0.000938525):0,(Barbus_barbus_MG495791_MG495641:0.00445221,Barbus_barbus_MG495855_MG495705:0.000669597)0.581:0.00110501):0,Barbus_barbus_MG495794_MG495644:0.00166342):0,Barbus_barbus_MG495806_MG495656:0.000979925):0,Barbus_barbus_MG495833_MG495683:0.00801887):0,Barbus_barbus_MG495844_MG495694:0.000842699):0,Barbus_barbus_MG495847_MG495697:0.00129309):0,Barbus_barbus_MG495853_MG495703:0.000450939):0,Barbus_barbus_MG495854_MG495704:0.00429877):0,Barbus_barbus_MG495870_MG495720:0.000789867):0,Barbus_barbus_MG495879_MG495729:0.00111567):0,Barbus_barbus_MG495886_MG495736:0.00111032):0,Barbus_barbus_MG495888_MG495738:0.00165752)1.000:0.0204966)1.000:0.0115965,((((Barbus_balcanicus_MG495786_MG495636:0.000811327,(Barbus_balcanicus_MG495787_MG495637:0.00114407,Barbus_balcanicus_MG495865_MG495715:0.00274487)0.979:0.00131604)0.795:0.00123466,(Barbus_balcanicus_MG495788_MG495638:0.00450255,Barbus_balcanicus_MG495885_MG495735:0.0040159)0.954:0.00242227)1.000:0.0302469,(Barbus_balcanicus_MG495789_MG495639:0.000469584,Barbus_balcanicus_MG495887_MG495737:0.00115159)1.000:0.00752469)1.000:0.0228445,(((Barbus_caninus_MG495792_MG495642:0.00130946,Barbus_caninus_MG495868_MG495718:0.00137985)1.000:0.00554815,Barbus_caninus_MG495793_MG495643:0.0114327)1.000:0.0177382,Barbus_caninus_MG495867_MG495717:0.00394172)1.000:0.0198989)1.000:0.0133463)1.000:0.021215,(Luciobarbus_graellsii_MG495843_MG495693:0.000536983,Luciobarbus_graellsii_JN049525_MG827110:0.0434958)0.913:0.0263204)0.913:0.0219546):0.00546621);

tree RAxML_tree = ((Luciobarbus_graellsii_JN049525_MG827110:0.0233099,(Luciobarbus_graellsii_MG495843_MG495693:1e-06,((Luciobarbus_graellsii_MG495838_MG495688:0.00121848,Luciobarbus_graellsii_MG495839_MG495689:1e-06)100:0.162195,((((Barbus_balcanicus_MG495789_MG495639:1e-06,Barbus_balcanicus_MG495887_MG495737:0.000905281)100:0.0102597,((Barbus_balcanicus_MG495885_MG495735:0.00504257,Barbus_balcanicus_MG495788_MG495638:0.00562508)76:0.00261203,(Barbus_balcanicus_MG495786_MG495636:0.000617127,(Barbus_balcanicus_MG495865_MG495715:0.00308844,Barbus_balcanicus_MG495787_MG495637:0.000947801)67:0.00126366)60:0.000826183)100:0.0391964)100:0.0305669,(Barbus_caninus_MG495867_MG495717:0.00475396,(Barbus_caninus_MG495793_MG495643:0.0142245,(Barbus_caninus_MG495792_MG495642:0.0011268,Barbus_caninus_MG495868_MG495718:0.00136928)99:0.00694865)100:0.0225912)100:0.0246805)98:0.0166268,(((Barbus_tyberinus_TL_clade_MG495866_MG495716:1e-06,((((Barbus_tyberinus_TL_clade_MG495841_MG495691:0.000610375,(((Barbus_tyberinus_TL_clade_MG495823_MG495673:1e-06,Barbus_tyberinus_TL_clade_MG495837_MG495687:0.000611904)46:0.00060996,Barbus_tyberinus_TL_clade_MG495832_MG495682:1e-06)3:1e-06,Barbus_tyberinus_TL_clade_MG495840_MG495690:1e-06)12:1e-06)42:0.000609582,(((((((Barbus_tyberinus_TL_clade_MG495846_MG495696:1e-06,Barbus_tyberinus_TL_clade_MG495804_MG495654:1e-06)8:1e-06,Barbus_tyberinus_TL_clade_MG495845_MG495695:0.000609011)1:1e-06,(Barbus_tyberinus_TL_clade_MG495828_MG495678:0.000608711,(((Barbus_tyberinus_TL_clade_MG495819_MG495669:1e-06,Barbus_tyberinus_TL_clade_MG495817_MG495667:1e-06)9:1e-06,Barbus_tyberinus_TL_clade_MG495816_MG495666:0.000608641)2:1e-06,(Barbus_tyberinus_TL_clade_MG495815_MG495665:1e-06,Barbus_tyberinus_TL_clade_MG495814_MG495664:1e-06)13:1e-06)2:1e-06)0:1e-06)0:1e-06,Barbus_tyberinus_TL_clade_MG495826_MG495676:0.000608377)0:1e-06,(Barbus_tyberinus_TL_clade_MG495824_MG495674:1e-06,Barbus_tyberinus_TL_clade_MG495849_MG495699:0.00060806)1:1e-06)0:1e-06,(((Barbus_tyberinus_TL_clade_MG495813_MG495663:0.00306459,Barbus_tyberinus_TL_clade_MG495812_MG495662:0.00121935)55:1e-06,((Barbus_tyberinus_TL_clade_MG495820_MG495670:0.00061432,Barbus_tyberinus_TL_clade_MG495811_MG495661:0.00122988)66:0.000606423,Barbus_tyberinus_TL_clade_MG495850_MG495700:0.00121687)5:1e-06)0:1e-06,((Barbus_tyberinus_TL_clade_MG495808_MG495658:0.00244586,Barbus_tyberinus_TL_clade_MG495785_MG495635:0.00121872)6:1e-06,((Barbus_tyberinus_TL_clade_MG495830_MG495680:1e-06,Barbus_tyberinus_TL_clade_MG495851_MG495701:1e-06)65:0.00060789,(Barbus_tyberinus_TL_clade_MG495809_MG495659:1e-06,Barbus_tyberinus_TL_clade_MG495810_MG495660:0.00121643)97:0.00492169)16:1e-06)0:1e-06)0:1e-06)2:1e-06,Barbus_tyberinus_TL_clade_MG495852_MG495702:0.000608732)64:0.00121773)17:1e-06,(Barbus_tyberinus_TL_clade_MG495774_MG495624:0.00275997,Barbus_tyberinus_TL_clade_MG495796_MG495646:0.00121035)54:1.00809e-05)29:0.000608704,(Barbus_tyberinus_TL_clade_MG495835_MG495685:0.000609612,Barbus_tyberinus_TL_clade_MG495834_MG495684:1e-06)41:1e-06)53:0.00137261)100:0.0101925,(((Barbus_sp_clade_4_MK728816_MK728798:0.00605815,((Barbus_sp_clade_4_MG718026_MK728800:0.000825528,(Barbus_sp_clade_4_MG718025_MK728797:1e-06,Barbus_sp_clade_4_MG718025_MK728799:1e-06)47:1e-06)41:1e-06,Barbus_sp_clade_4_MG718025_MK728801:0.000825238)81:0.00161067)34:0.000328792,((Barbus_sp_clade_4_MK728817_MK728810:1e-06,((Barbus_sp_clade_4_MK728817_MK728815:0.000614427,(Barbus_sp_clade_4_MK728817_MK728811:1e-06,((Barbus_sp_clade_4_MK728817_MK728805:0.000614109,(Barbus_sp_clade_4_MK728817_MK728813:1e-06,Barbus_sp_clade_4_MK728817_MK728802:1e-06)13:1e-06)2:1e-06,Barbus_sp_clade_4_MK728817_MK728803:1e-06)2:1e-06)14:1e-06)19:1e-06,(Barbus_sp_clade_4_MK728817_MK728814:1e-06,Barbus_sp_clade_4_MK728817_MK728812:1e-06)66:0.000614415)77:0.00123186)89:0.00353385,((Barbus_sp_TSAAC_clade_MK728821_MK728809:1e-06,((((Barbus_sp_TSAAC_clade_MG495913_MG495763:1e-06,(Ofanto_10-MBarbus_sp_TSAAC_clade_MG495896_MG495746:1e-06,(((Ofanto_08-MBarbus_sp_TSAAC_clade_MG495893_MG495743:1e-06,Ofanto_05-MBarbus_sp_TSAAC_clade_MG495894_MG495744:1e-06)8:1e-06,((Barbus_sp_TSAAC_clade_MG495914_MG495764:1e-06,Barbus_sp_TSAAC_clade_MG495912_MG495762:1e-06)11:1e-06,Barbus_sp_TSAAC_clade_MG495915_MG495765:1e-06)3:1e-06)2:1e-06,(Barbus_sp_TSAAC_clade_MG495922_MG495772:1e-06,Barbus_sp_TSAAC_clade_MG495918_MG495768:1e-06)7:1e-06)5:1e-06)81:1e-06)45:1e-06,(((Barbus_sp_TSAAC_clade_MG495798_MG495648:1e-06,(((Barbus_sp_TSAAC_clade_MG495800_MG495650:1e-06,Barbus_sp_TSAAC_clade_MG495875_MG495725:0.00121576)8:1e-06,Barbus_sp_TSAAC_clade_MG495802_MG495652:1e-06)2:1e-06,Barbus_sp_TSAAC_clade_MG495803_MG495653:1e-06)5:1e-06)7:1e-06,Barbus_sp_TSAAC_clade_MG495799_MG495649:0.00310906)76:1e-06,(Barbus_sp_TSAAC_clade_MG495874_MG495724:0.00124054,Barbus_sp_TSAAC_clade_MG495876_MG495727:0.00372985)76:0.00120569)6:1e-06)0:1e-06,(((((Barbus_sp_TSAAC_clade_MK728819_MK728808:1e-06,Barbus_sp_TSAAC_clade_MK728819_MK728806:1e-06)75:1e-06,Barbus_sp_TSAAC_clade_MG495797_MG495647:1e-06)57:1e-06,(Barbus_sp_TSAAC_clade_MG495801_MG495651:1e-06,Barbus_sp_TSAAC_clade_MK728819_MK728807:1e-06)61:0.000608443)92:0.00244163,((Barbus_sp_TSAAC_clade_MG495899_MG495749:0.00121891,(Barbus_sp_TSAAC_clade_MG495898_MG495748:0.00122156,Barbus_sp_TSAAC_clade_MG495893_MG495743:0.00061052)27:1e-06)78:0.0080735,((Barbus_sp_TSAAC_clade_MG495878_MG495728:0.00122114,((Barbus_sp_TSAAC_clade_MG495896_MG495746:1e-06,Barbus_sp_TSAAC_clade_MG495894_MG495744:1e-06)17:1e-06,((Barbus_sp_TSAAC_clade_MG495917_MG495767:1e-06,Barbus_sp_TSAAC_clade_MG495920_MG495770:1e-06)86:1e-06,(Barbus_sp_TSAAC_clade_MG495900_MG495750:1e-06,Barbus_sp_TSAAC_clade_MG495895_MG495745:1e-06)16:1e-06)1:1e-06)21:1e-06)12:1e-06,Barbus_sp_TSAAC_clade_MG495897_MG495747:0.000609333)70:0.000611435)12:0.000607791)4:1e-06,Barbus_sp_TSAAC_clade_MG495877_MG495726:0.00121968)0:1e-06)2:1e-06,Barbus_sp_TSAAC_clade_MK728820_MK728804:0.000615097)7:0.00121971)1:1e-06,(Barbus_sp_TSAAC_clade_MG495773_MG495623:0.000908716,Barbus_sp_TSAAC_clade_MG495775_MG495625:1e-06)64:0.000607937)65:0.00891714)18:0.00389396)42:0.00536229,((((((((((Barbus_plebejus_PV_clade_MG495860_MG495710:1e-06,(Barbus_plebejus_PV_clade_MG495782_MG495632:0.000918479,(((Barbus_plebejus_PV_clade_MG495856_MG495706:1e-06,Barbus_plebejus_PV_clade_MG495778_MG495628:0.00163445)5:1e-06,Barbus_plebejus_PV_clade_MG495779_MG495629:1e-06)7:1e-06,Barbus_plebejus_PV_clade_MG495859_MG495709:0.0007632)51:0.000772493)22:3.86703e-05)6:1e-06,(((Barbus_plebejus_PV_clade_MG495872_MG495722:1e-06,(Barbus_plebejus_PV_clade_MG495871_MG495721:0.000603365,((Barbus_plebejus_PV_clade_MG495910_MG495760:0.00470691,Barbus_plebejus_PV_clade_MG495909_MG495759:0.00277867)63:0.00180941,Barbus_plebejus_PV_clade_MG495869_MG495719:0.0133488)98:0.003652)70:0.00123186)26:0.000608673,Barbus_plebejus_PV_clade_MG495836_MG495686:0.000608368)1:1e-06,Barbus_plebejus_PV_clade_MG495904_MG495754:0.000608753)0:1e-06)0:1e-06,(((Barbus_plebejus_PV_clade_MG495903_MG495753:0.00245519,(((Barbus_plebejus_PV_clade_MG495861_MG495711:0.000910576,Barbus_plebejus_PV_clade_MG495881_MG495731:0.00091113)17:1e-06,(Barbus_plebejus_PV_clade_MG495858_MG495708:0.00152671,Barbus_plebejus_PV_clade_MG495906_MG495756:1e-06)26:0.00122072)3:1e-06,(Barbus_plebejus_PV_clade_MG495842_MG495692:1e-06,Barbus_plebejus_PV_clade_MG495805_MG495655:1e-06)72:1e-06)3:1e-06)3:0.000608038,(((Barbus_plebejus_PV_clade_MG495889_MG495739:0.000610647,(Barbus_plebejus_PV_clade_MG495892_MG495742:0.000608561,Barbus_plebejus_PV_clade_MG495902_MG495752:0.000611073)10:1e-06)15:1e-06,Barbus_plebejus_PV_clade_MG495905_MG495755:0.0018453)28:0.000608185,((Barbus_plebejus_PV_clade_MG495777_MG495627:0.000919412,Barbus_plebejus_PV_clade_MG495891_MG495741:0.000919417)6:1e-06,Barbus_plebejus_PV_clade_MG495776_MG495626:1e-06)14:1e-06)5:1e-06)1:0.000608041,Barbus_plebejus_PV_clade_MG495818_MG495668:0.000608801)0:1e-06)0:1e-06,(Barbus_plebejus_PV_clade_MG495822_MG495672:1e-06,Barbus_plebejus_PV_clade_MG495907_MG495757:0.000608464)8:1e-06)0:1e-06,((Barbus_plebejus_PV_clade_MG495848_MG495698:0.000609281,Barbus_plebejus_PV_clade_MG495857_MG495707:1e-06)16:1e-06,(Barbus_plebejus_PV_clade_MG495908_MG495758:0.00183401,(Barbus_plebejus_PV_clade_MG495901_MG495751:0.00122451,(Barbus_plebejus_PV_clade_MG495873_MG495723:0.00366491,Barbus_plebejus_PV_clade_MG495829_MG495679:0.00060184)7:8.62217e-06)3:1e-06)10:0.000608946)3:0.000608552)15:0.000608176,Barbus_plebejus_PV_clade_MG495911_MG495761:0.00183279)2:1e-06,Barbus_plebejus_PV_clade_MG495862_MG495712:0.000608173)0:1e-06,(((Barbus_plebejus_PV_clade_MG495864_MG495714:0.00184755,(Barbus_plebejus_PV_clade_MG495780_MG495630:0.000911262,Barbus_plebejus_PV_clade_MG495882_MG495732:1e-06)29:1e-06)39:0.000753652,((Barbus_plebejus_PV_clade_MG495890_MG495740:0.00745131,Barbus_plebejus_PV_clade_MG495884_MG495734:0.00465378)30:1e-06,Barbus_plebejus_PV_clade_MG495883_MG495733:1e-06)51:0.000885133)20:0.000202715,Barbus_plebejus_PV_clade_MG495825_MG495675:0.00121864)7:1e-06)0:1e-06,(Barbus_plebejus_PV_clade_MG495827_MG495677:0.00121572,Barbus_plebejus_PV_clade_MG495863_MG495713:0.00184763)4:1e-06)49:0.000940213,Barbus_plebejus_PV_clade_MG495781_MG495631:1e-06)76:0.00552647)36:0.00266918)98:0.0088906,(Barbus_barbus_MG495784_MG495634:0.00183695,(Barbus_barbus_MG495880_MG495730:0.000915704,(((Barbus_barbus_MG495888_MG495738:0.00182909,((Barbus_barbus_MG495783_MG495633:0.00127026,(Barbus_barbus_MG495821_MG495671:0.00294306,Barbus_barbus_MG495807_MG495657:1e-06)63:0.000731934)81:0.00122947,(Barbus_barbus_MG495795_MG495645:0.0012238,Barbus_barbus_MG495831_MG495681:0.0012234)70:0.0006119)76:0.00183513)7:1e-06,Barbus_barbus_MG495844_MG495694:0.000608105)12:1e-06,(((((((Barbus_barbus_MG495854_MG495704:0.00554979,(Barbus_barbus_MG495794_MG495644:0.00181876,(Barbus_barbus_MG495847_MG495697:0.00122239,(Barbus_barbus_MG495833_MG495683:0.00999594,Barbus_barbus_MG495870_MG495720:1e-06)30:0.000609335)12:0.000601893)1:1e-06)1:1e-06,Barbus_barbus_MG495886_MG495736:0.000907146)1:1e-06,Barbus_barbus_MG495879_MG495729:0.000919057)0:1e-06,Barbus_barbus_MG495790_MG495640:0.000918368)0:1e-06,Barbus_barbus_MG495853_MG495703:1e-06)0:1e-06,(Barbus_barbus_MG495791_MG495641:0.00559342,Barbus_barbus_MG495855_MG495705:1e-06)41:0.000917537)1:1e-06,Barbus_barbus_MG495806_MG495656:0.000769349)2:1e-06)27:0.000800284)8:1e-06)100:0.0259503)98:0.0155351)99:0.0283066)31:0.0347787)26:0.0346128):0.184928,Cyprinus_carpio_DQ868875_JN105352:0.184928);

end;
