# Supplementary material for: Mitochondrial phylogeny and taxonomic revision of Italian and Slovenian fluvio-lacustrine barbels, Barbus sp. (Cypriniformes, Cyprinidae)
Source: BMC Zool. 2021 Apr 21;6:8. doi: 10.1186/s40850-021-00073-x (PMC10127354; doi:10.1186/s40850-021-00073-x)
Supplement: Supplementary file 6 — Additional file 6. Tests for chance occurrence of reciprocal monophyly. All analyses were carried out using the Species Delimitation Plugin of Geneious Prime® 2021.0.3. [file 40850_2021_73_MOESM6_ESM.pdf]

Additional file 6. Tests for chance occurrence of reciprocal monophyly.

Tree	Taxon	Closest taxon	Monophyletic?	Intra Dist[a]	Inter Dist-Closest[b]	Intra/Inter[c]	P ID(Strict)[d]	P ID(Liberal)[e]	Av(MRCA-tips)[f]	Rosenberg's P_{AB}[g]
RAxML	NAAC	TSAAC	no	0.007	0.018	0.38	0.85 (0.78, 0.92)	0.95 (0.91, 1.00)	0.0047	n/a
	PV	NAAC	yes	0.005	0.022	0.24	0.91 (0.86, 0.97)	0.97 (0.94, 1.00)	0.0040	2.10E-31
	TL	PV	yes	0.003	0.026	0.11	0.95 (0.90, 1.00)	0.98 (0.96, 1.00)	0.0039	2.00E-34
	TSAAC	NAAC	yes	0.004	0.018	0.20	0.92 (0.87, 0.98)	0.97 (0.95, 1.00)	0.0030	3.10E-11
MrBayes	NAAC	PV	yes	0.006	0.021	0.27	0.88 (0.82, 0.95)	0.96 (0.92, 1.00)	0.0068	4.50E-19
	PV	TSAAC	yes	0.005	0.020	0.24	0.91 (0.86, 0.97)	0.97 (0.94, 1.00)	0.0042	6.30E-27
	TL	PV	yes	0.003	0.024	0.14	0.94 (0.89, 1.00)	0.98 (0.95, 1.00)	0.0044	2.00E-34
	TSAAC	PV	yes	0.004	0.020	0.20	0.93 (0.87, 0.98)	0.97 (0.95, 1.00)	0.0070	6.30E-27
IQ-TREE	NAAC	PV	yes	0.005	0.022	0.24	0.90 (0.83, 0.96)	0.97 (0.92, 1.00)	0.0064	4.50E-19
	PV	NAAC	yes	0.005	0.022	0.23	0.92 (0.86, 0.97)	0.97 (0.94, 1.00)	0.0063	6.30E-27
	TL	PV	yes	0.003	0.025	0.11	0.95 (0.90, 1.00)	0.99 (0.96, 1.00)	0.0036	2.00E-34
	TSAAC	PV	yes	0.003	0.024	0.14	0.94 (0.89, 1.00)	0.98 (0.95, 1.00)	0.0091	6.30E-27

[a] Intra-taxon average pairwise tree distance.

[b] Average pairwise tree distance between members of the taxon and members of the next closest taxon.

[c] The ratio of Intra-Dist to Inter Dist-Closest.

[d] The mean probability, with the 95% confidence interval (CI) for the prediction, of making a correct identification of an unknown specimen of the focal taxon using placement on a tree and the criterion that it must fall within, but not sister to, the taxon clade.

[e] The mean probability, with the 95% confidence interval (CI) for the prediction, of making a correct identification of an unknown specimen of the focal taxon using best sequence alignment, closest genetic distance or placement on a tree, with the criterion that it falls sister to or within a monophyletic taxon clade.

[f] The mean distance between the most recent common ancestor of a taxon and its members.

[g] The probability that taxon A represented by *a* sequences, in a clade of *a* + *b* sequences, will be reciprocally monophyletic with the remaining *b* sequences under the null model of random coalescence.
