# Supplementary figures and images for: Mitochondrial phylogeny and taxonomic revision of Italian and Slovenian fluvio-lacustrine barbels, Barbus sp. (Cypriniformes, Cyprinidae)
Source: BMC Zool. 2021 Apr 21;6:8. doi: 10.1186/s40850-021-00073-x (PMC10127354; doi:10.1186/s40850-021-00073-x)

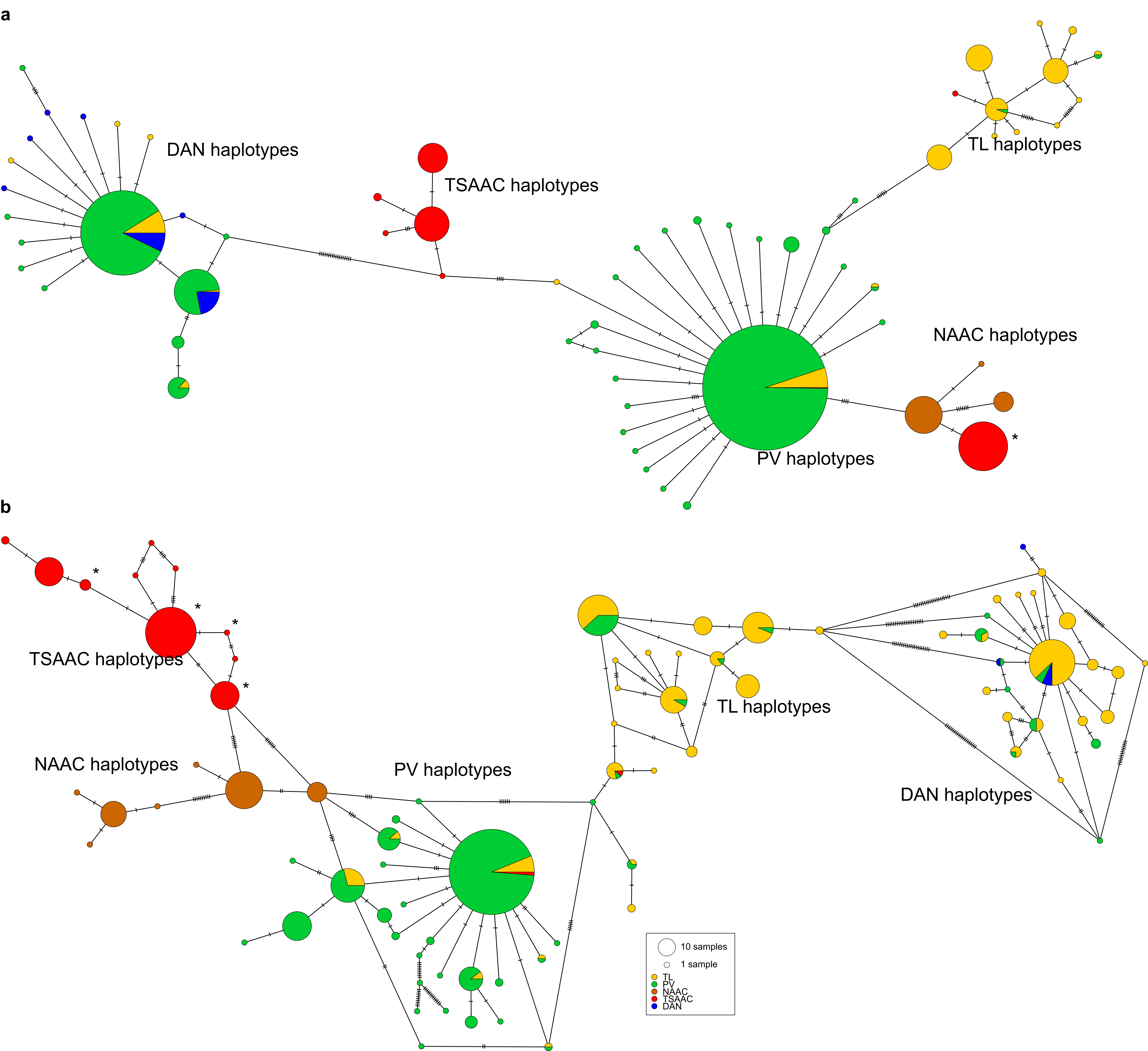

Supplement: Supplementary file 7 — Additional file 7. Single-marker minimum spanning networks computed from sequences and geographical abundancies listed in S4. a, cytb; b, D-loop. Asterisks mark samples that was not possible to unambiguously assign to an ichthyogeographical district (see also Fig. 4). [file 40850_2021_73_MOESM7_ESM.pdf]
