# Supplementary material for: Mitochondrial phylogeny and taxonomic revision of Italian and Slovenian fluvio-lacustrine barbels, Barbus sp. (Cypriniformes, Cyprinidae)
Source: BMC Zool. 2021 Apr 21;6:8. doi: 10.1186/s40850-021-00073-x (PMC10127354; doi:10.1186/s40850-021-00073-x)
Supplement: Supplementary file 8 — Additional file 8. Meristic data of Barbus plebejus and B. tyberinus from [28, 33, 34] and morphometric and meristic data of B. oscensis (i.e., the B. tyberinus TSAAC clade). [file 40850_2021_73_MOESM8_ESM.pdf]

Additional file 8. Meristic data of *Barbus plebejus* and *B. tyberinus* from Bianco [28,33-34] and morphometric and meristic data of *B. oscensis* sp. nov. (i.e., the *Barbus* sp. TSAAC clade).

	B. plebejus (N=153)			***B. tyberinus*** (N=168)			***B. oscensis* sp. nov.** (N=6)	
	Lectotype	Paralectotypes	Range[a,b]	Lectotype	Paralectotypes	Range[a,b]	Holotype	Paratypes[c]
Number of specimens	1	5	153	1	33	167	1	5
Standard length (mm)	268	111-194	42-330	122	50-188	38-373	160	131-156 (143.95±10.23)
Meristic characters								
Number of scales								
lateral line	69	66-72	62[66-72]78	55	47-61	47[51-58]63	55	51-57 (53.90±2.04)
above lateral line (+0.5)[d]	15	13-15	12[15]16	15	11-15	10[12-14]15	14	12-14 (13.10±0.89)
below lateral line (+0.5)[d]	8	8-10	8[10-11]12	9	7-9	7[8-9]10	11	8-12 (9.60±1.47)
circumpeduncolar	28	28-32	26[28]32	26	22-28	22[24-26]28	26	24-28 (25.80±1.64)
Number of branched rays								
dorsal fin	8	8	[8]	8	8	[8]	8	8
anal fin	5	5	[5]	5	5	[5]	5	5
pelvic fin	8	8	7[8]	8	8	[8]	8	8
Number of gill rakers								
total	10	10-11	9[10-12]15	8	8-10	7[8-10]13	11	9-11 (10.40±0.89)

Lower arch	7	7-9	7[8-10]12	6	6-8	5[6-8]10	8	7-9 (8.00±0.71)
Upper arch	3	2-3	[2]3	2	1-3	0[2-3]4	3	2-3 (2.40±0.55)
Pharyngeal teeth								
Left side	-		4.3.2.[5.3.2.]	-		4.3.2.[5.3.2.]	5.3.2	5.3.2-4.3.2
Right side	-		4.3.2.[5.3.2.]	5.3.2		4.3.2.[5.3.2.]	5.3.2	5.3.2-4.3.2
Serrae on ossified ray of fin	30	25-34	0-38	18		0-34	0	0
Morphometric characters (mm)								
Eye diameter							6.57	5.73-7.2 (6.35±0.58)
Preorbital distance							15.66	13.51-15.93 (14.57±0.98)
Mouth-operculum distance							43.35	35.44-41.3 (39.15±2.62)
Length of pectoral fin							32.65	23.14-32.97 (28.61±3.77)
Length of ventral fin							26.03	21.63-26.99 (23.99±2.39)
Length of anal fin							38.54	25.98-36.67 (31.19±3.97)
Height of the third dorsal fin ossified ray							31.33	24.62-31.43 (27.84±2.72)

[a] Range includes also non-type specimens.

[b] Usual values are included in brackets.

[c] Mean ±standard deviation is included in parentheses.

[d] Tabulated values must be conventionally added of +0.5.
