# Supplementary material for: Mitochondrial phylogeny and taxonomic revision of Italian and Slovenian fluvio-lacustrine barbels, Barbus sp. (Cypriniformes, Cyprinidae)
Source: BMC Zool. 2021 Apr 21;6:8. doi: 10.1186/s40850-021-00073-x (PMC10127354; doi:10.1186/s40850-021-00073-x)
Supplement: Supplementary file 9 — Additional file 9. Meristic data of Barbus plebejus (N = 153) and Barbus tyberinus (N = 168) from [28, 33, 34] and Barbus oscensis (i.e., the B. tyberinus TSAAC clade; N = 6) from original counts. Vertical lines: observed range; dithered boxes and horizontal lines: usual values. Characters with no evident variability between subspecies are not shown. [file 40850_2021_73_MOESM9_ESM.pdf]

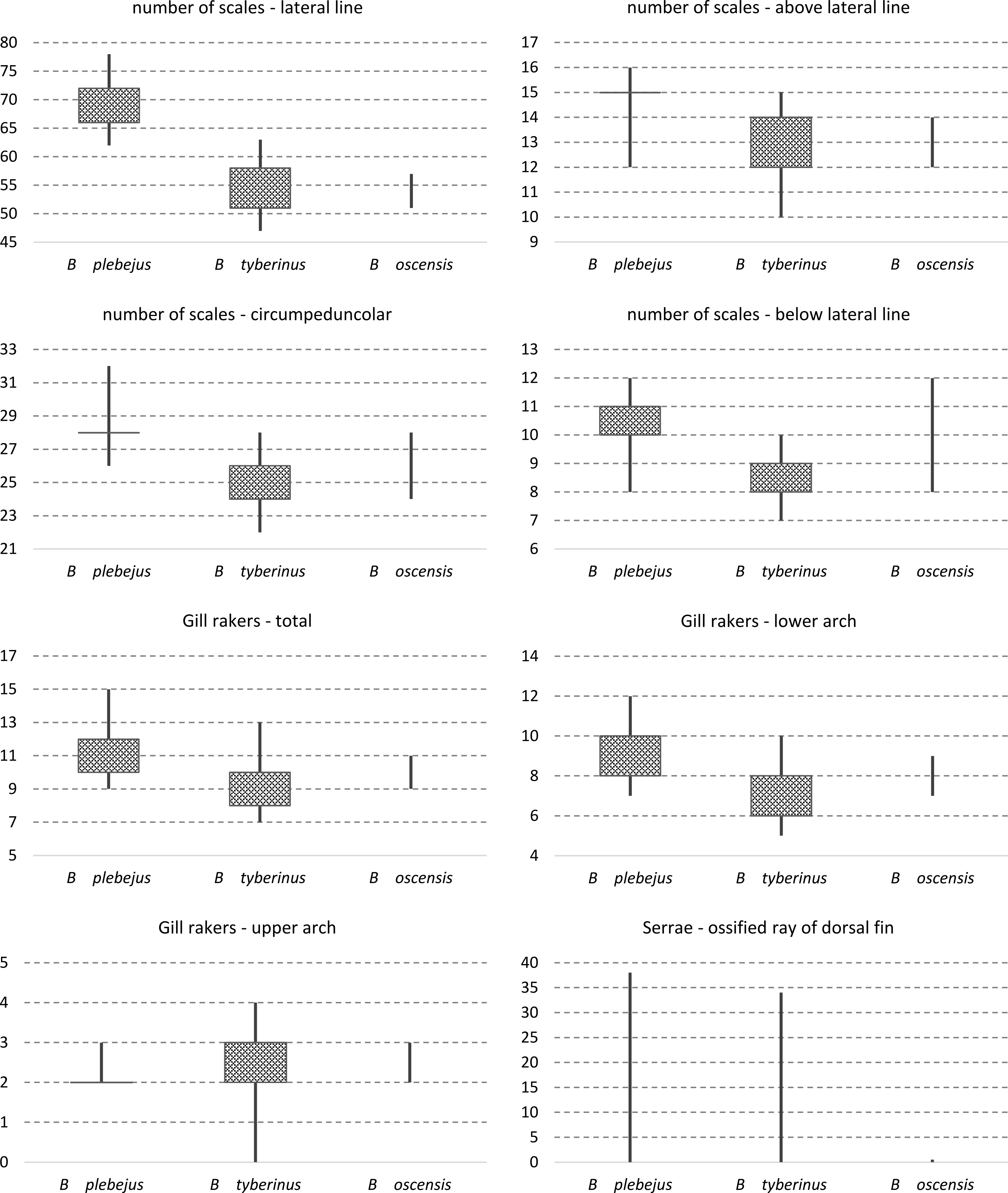
number of scales - lateral line
80
75
70
65
60
55
50
45
B plebejus
B tyberinus
B oscensis
number of scales - above lateral line
17
16
15
14
13
12
11
10
9
B plebejus
B tyberinus
B oscensis
number of scales - circumpeduncolar
33
31
29
27
25
23
21
B plebejus
B tyberinus
B oscensis
number of scales - below lateral line
13
12
11
10
9
8
7
6
B plebejus
B tyberinus
B oscensis
Gill rakers - total
17
15
13
11
9
7
5
B plebejus
B tyberinus
B oscensis
Gill rakers - lower arch
14
12
10
8
6
4
B plebejus
B tyberinus
B oscensis
Gill rakers - upper arch
5
4
3
2
1
0
B plebejus
B tyberinus
B oscensis
Serrae - ossified ray of dorsal fin
40
35
30
25
20
15
10
5
0
B plebejus
B tyberinus
B oscensis
