# Supplementary material for: Mitochondrial phylogeny and taxonomic revision of Italian and Slovenian fluvio-lacustrine barbels, Barbus sp. (Cypriniformes, Cyprinidae)
Source: BMC Zool. 2021 Apr 21;6:8. doi: 10.1186/s40850-021-00073-x (PMC10127354; doi:10.1186/s40850-021-00073-x)
Supplement: Supplementary file 10 — Additional file 10. Tukey post-hoc test results. The table lists the Q value; the p-value is shown in parentheses. ***, p < 0.001; **, p < 0.01. NAAC, Barbus sp. clade 4; TSAAC, Barbus sp. TSAAC clade; TL, B. tyberinus TL clade; PV, B. plebejus PV clade; DAN, B. barbus clade. [file 40850_2021_73_MOESM10_ESM.pdf]

Additional file 10. Tukey post-hoc test results.

	TSAAC	TL	PV	DAN
Number of dorsal fin branched rays				
NAAC	2.7695 (p=0.2881)	5.539 (p=0.001**)	2.7695 (p=0.2881)	5.539 (p=0.001**)
TSAAC		2.7695 (p=0.2881)	5.539 (p=0.001**)	2.7695 (p=0.2881)
TL			8.3085 (p=0***)	0 (p=1)
PV				8.3085 (p=0***)
Number of scales above the lateral line				
NAAC	5.9921 (p=0.0003***)	9.7905 (p=0***)	20.4711 (p=0***)	9.7905 (p=0***)
TSAAC		3.7985 (p=0.0574**)	14.4791 (p=0***)	3.7985 (p=0.0574**)
TL			10.6806 (p=0***)	0 (p=1)
PV				10.6806 (p=0***)
Number of scales on the lateral line				
NAAC	1.5933 (p=0.7924)	0.5716 (p=0.9944)	19.4353 (p=0***)	3.1439 (p=0.1728)
TSAAC		2.165 (p=0.5428)	21.0286 (p=0***)	4.7373 (p=0.0077**)
TL			18.8636 (p=0***)	2.5723 (p=0.3637)
PV				16.2913 (p=0***)
Number of scales under the lateral line				
NAAC	9.3217 (p=0***)	6.5527 (p=0***)	15.2896 (p=0***)	5.4606 (p=0.0012**)
TSAAC		2.769 (p=0.2883)	5.9679 (p=0.0003***)	3.8611 (p=0.051**)
TL			8.7369 (p=0***)	1.0921 (p=0.9385)
PV				9.829 (p=0***)
